# Transforming Growth Factor β Drives Hemogenic Endothelium Programming and the Transition to Hematopoietic Stem Cells

**DOI:** 10.1016/j.devcel.2016.06.024

**Published:** 2016-08-22

**Authors:** Rui Monteiro, Philip Pinheiro, Nicola Joseph, Tessa Peterkin, Jana Koth, Emmanouela Repapi, Florian Bonkhofer, Arif Kirmizitas, Roger Patient

**Affiliations:** 1 Molecular Haematology Unit, Weatherall Institute of Molecular Medicine, John Radcliffe Hospital, University of Oxford, Oxford OX3 9DS, UK; 2 BHF Centre of Research Excellence, Oxford, UK; 3 Computational Biology Research Group, Weatherall Institute of Molecular Medicine, John Radcliffe Hospital, University of Oxford, Oxford OX3 9DS, UK

**Keywords:** TGFβ, notch, hematopoietic stem cell, EHT, zebrafish, jag1a

## Abstract

Hematopoietic stem cells (HSCs) are self-renewing multipotent stem cells that generate mature blood lineages throughout life. They, together with hematopoietic progenitor cells (collectively known as HSPCs), emerge from hemogenic endothelium in the floor of the embryonic dorsal aorta by an endothelial-to-hematopoietic transition (EHT). Here we demonstrate that transforming growth factor β (TGFβ) is required for HSPC specification and that it regulates the expression of the Notch ligand Jagged1a in endothelial cells prior to EHT, in a striking parallel with the epithelial-to-mesenchymal transition (EMT). The requirement for TGFβ is two fold and sequential: autocrine via Tgfβ1a and Tgfβ1b produced in the endothelial cells themselves, followed by a paracrine input of Tgfβ3 from the notochord, suggesting that the former programs the hemogenic endothelium and the latter drives EHT. Our findings have important implications for the generation of HSPCs from pluripotent cells in vitro.

## Highlights

• TGFβ signaling is required for hematopoietic stem cell (HSC) emergence in embryos
• TGFβ regulates *jag1a* expression and programs endothelium to become hemogenic endothelium (HE)
• Tgfb1a/Tgfb1b and Tgfb3 act sequentially to program HE and give rise to HSCs

## Graphical Abstract

**Figure:**
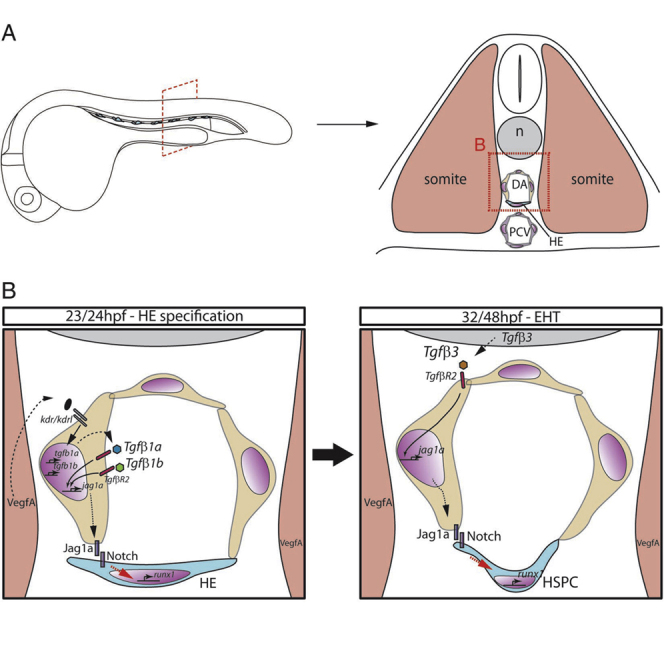

## Teaser

Hematopoietic stem cells (HSCs) arise in the embryo from hemogenic endothelium in the dorsal aorta. Here, Monteiro et al. reveal that sequential activity by two TGFβ ligands and crosstalk between TGFβ and Notch signaling are required to program arterial endothelial cells to become hemogenic and give rise to HSCs.

## Introduction

Hematopoietic stem cells (HSCs) are specified during embryonic development from a subset of arterial endothelial cells located in the floor of the dorsal aorta (DA). HSCs emerge by a process termed the endothelial-to-hematopoietic transition (EHT) (Bertrand et al., 2010, Boisset et al., 2010, Kissa and Herbomel, 2010). In zebrafish, the hematopoietic stem and progenitor cells (HSPCs) generated by EHT migrate to the caudal hematopoietic tissue (CHT), where they proliferate and undergo differentiation into erythroid and myeloid lineages (Kissa et al., 2008, Murayama et al., 2006). Some will exit the CHT and migrate to the thymus to give rise to T cells, and others move to the kidney, the adult site of hematopoiesis in the zebrafish, equivalent to the bone marrow in mammals (Ciau-Uitz et al., 2014).

The transcription factor Runx1 is required for EHT in mice and zebrafish (Chen et al., 2009, Kissa and Herbomel, 2010). Its expression in the floor of the DA is initiated by 23 hpf in zebrafish (Wilkinson et al., 2009) and marks a cell population committed to the hemogenic fate, the hemogenic endothelium (HE). Several signaling pathways including Hedgehog, VEGF, Notch and BMP are required sequentially to regulate programming of the arterial endothelium and HSPC emergence (Burns et al., 2005, Gering and Patient, 2005, Kim et al., 2014, Wilkinson et al., 2009). The Notch receptor Notch1 is the main driver of HSPC emergence from HE, likely downstream of its ligand Jagged1 (Gama-Norton et al., 2015, Hadland et al., 2015, Jang et al., 2015) and is thought to drive *runx1* expression via Gata2 (Robert-Moreno et al., 2005). Jagged1 is dispensable for arterial programming but required in the endothelium for the specification of HSPCs (Espin-Palazon et al., 2014, Gama-Norton et al., 2015, Robert-Moreno et al., 2008).

In humans, defective transforming growth factor β (TGFβ) signaling is associated with proliferative disorders of HSPCs such as acute myeloid leukemia and T cell acute lymphoblastic leukemia (Kim and Letterio, 2003). More recently, it has been shown that paracrine TGFβ signaling in the bone marrow niche maintains quiescence of the resident HSC pool (Zhao et al., 2014) and may also direct differentiation of lineage-biased HSC subtypes (Challen et al., 2010), positioning TGFβ as a critical regulator of proliferation and differentiation of adult HSCs. Whether TGFβ plays a role in the formation of HSCs is however not known. Mutants for the ligand TGFβ1 or its receptor TGFβR2, including endothelial-specific conditional knockout mice, die between E9.5 and E10.5 due to defective recruitment of mural cells to the yolk sac vasculature and the subsequent loss of vessel integrity (Carvalho et al., 2004, Dickson et al., 1995, Oshima et al., 1996). This is before the emergence of HSPCs in the embryo proper (de Bruijn et al., 2002), effectively precluding the analysis of the role of TGFβ signaling in HSPC specification in mice. Zebrafish, however, develop externally and do not depend on extraembryonic tissues for survival. In addition, recruitment of mural cells to the endothelium does not happen until 72 hpf (Santoro et al., 2009), 2 days after the HSPCs are specified in the DA. Thus, we can address the role of TGFβ in HSPC emergence in zebrafish without the inherent limitations of the mouse models.

The TGFβ superfamily comprises BMPs, Activins, Nodals, and TGFβs. There are three TGFβ ligands in the mouse: TGFβ1, TGFβ2, and TGFβ3 (Goumans and Mummery, 2000), and they all signal through a single type II serine-threonine kinase receptor (TGFβR2) that recruits the type I receptors Activin-like kinase 1 (Alk1) or Alk5 (Shi and Massague, 2003). Alk1 expression is essentially restricted to endothelial cells (ECs) (Oh et al., 2000), whereas Alk5 is more broadly expressed (Goumans et al., 2002) but also present in ECs. Activated Alk1 phosphorylates Smad1, Smad5, and Smad9, whereas activated Alk5 phosphorylates Smad2 and Smad3 (Shi and Massague, 2003). Activated Smads migrate to the nucleus together with the co-Smad Smad4 and regulate transcription together with co-activators or co-repressors (Shi and Massague, 2003). In addition, TGFβ can signal through the non-canonical Erk, JNK, and p38 MAPK kinase pathways to instigate transcriptional responses (Derynck and Zhang, 2003). Thus, to circumvent the complexity of the intracellular signaling elicited by TGFβ, we focused our attention on TgfβR2, the type II receptor for TGFβ. Abrogation of TGFβR2 activity revealed that TGFβ signaling plays a key role in the formation of HSPCs. TGFβ is required for the correct programming of the HE downstream of Vegf and independently of arterial programming. We demonstrate that Jag1a is a target of TGFβ signaling, and *jag1a* overexpression in endothelium rescues the loss of HSPCs in *tgfbR2*-depleted embryos. Finally, we identified two independent sources of ligand: TGFβ1a and TGFβ1b in the endothelium and TGFβ3 in the nearby notochord. Both inputs contribute to the regulation of *jag1a* in endothelium through the TgfβR2 receptor and thus enable Notch signaling to program the HE prior to specification of HSPCs.

## Results

### TGFβ Signaling Components Are Expressed in or around the Embryonic Site of HSC Emergence

**Figure 1:**
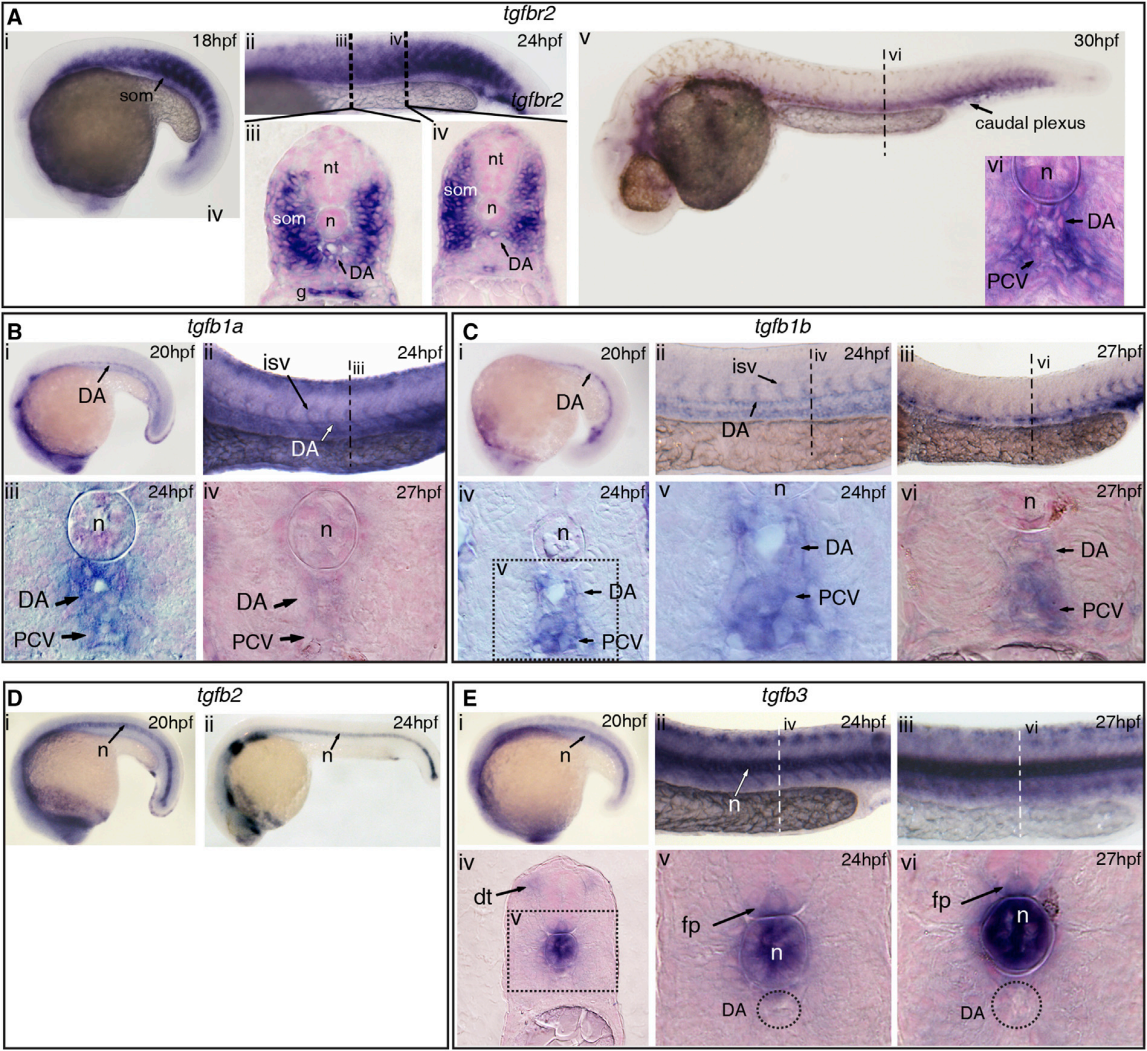
TGFβ Signaling Components Are Expressed in and around the Embryonic Dorsal Aorta (A) Expression of *tgfbR2* at (i) 18 hpf and (ii–iv) 24 hpf, including the somites, dorsal aorta (DA), and gut. (v–vi) At 30 hpf, expression was confined to the DA, notochord, posterior cardinal vein (PCV), and some of the surrounding mesenchyme. (B) Expression of *tgfb1a* in the DA at (i) 20 hpf and (ii, iii) in the DA, PCV, and intersomitic vessels (ISVs) at 24 hpf. At 27 hpf, there was very little expression of *tgfb1a* remaining in the DA. (C) *tgfb1b* is also expressed in the DA (i) at 20 hpf and in the DA and PCV at (ii) 24 hpf and (iii) 27 hpf. (iv, v) Transversal sections show *tgfb1b* expression at 24 hpf in the DA and PCV. (vi) *Tgfb1b* was still apparent in the DA and PCV by 27 hpf. (D) Expression of *tgfb2* at (i) 20 hpf and (ii) 24 hpf. Notochord-specific expression was found throughout all the stages analyzed. (E) Expression of *tgfb3* at (i) 20 hpf, (ii) 24 hpf, and (iii) 27 hpf. (iv, v) Transversal section at 24 hpf, showing expression in the dorsal tip of the somites, notochord, and floorplate. (vi) Expression in the notochord and floorplate was maintained at 27 hpf. Note that *tgfb3* is absent from the DA. g, gut; dt, dorsal tip of the somite; fp, floorplate; isv, intersomitic vessel; n, notochord; nt, neural tube; som, somite. See also Figure S1.

To investigate whether TGFβ signaling could play a role in HSPC specification in zebrafish, we first carried out expression analysis. *tgfβR2* is expressed in the head vasculature and in the somites at 15 hpf and in the DA and the somites from at least 18 hpf up to 24 hpf (Figures 1A and S1A), prior to the onset of *runx1* expression in the HE (Wilkinson et al., 2009). At 30 hpf, *tgfbR2* becomes essentially endothelial, with higher expression in the posterior cardinal vein (PCV) and in the caudal plexus (Figure 1A). TGFβ ligands are also expressed in the region at the onset of HE formation: *tgfb1a* and *tgfb1b* are expressed in the endothelium, including the DA at 15 hpf, 24 hpf, and 27 hpf (Figures 1B, 1C, S1B, and S1C). At 27 hpf, *tgfb1a* expression is downregulated in the DA and PCV, whereas *tgfb1b* is still clearly present (Figures 1B and 1C). T*gfb2* is expressed in the notochord at 12 hpf, 20 hpf, and 24 hpf (Figures 1D and S1D), and *tgfb3* is expressed in the notochord and in the 3–4 anterior-most somites from 12 hpf to 20 hpf and also in ECs in the head (Figures 1E and S1E). From 20 hpf onward, *tgfb3* was found in the dorsal tip of the somites, the floorplate, and in the notochord (Figure 1E).

### TGFβ Signaling through TgfβR2 Is Required for the Specification of HSPCs

**Figure 2:**
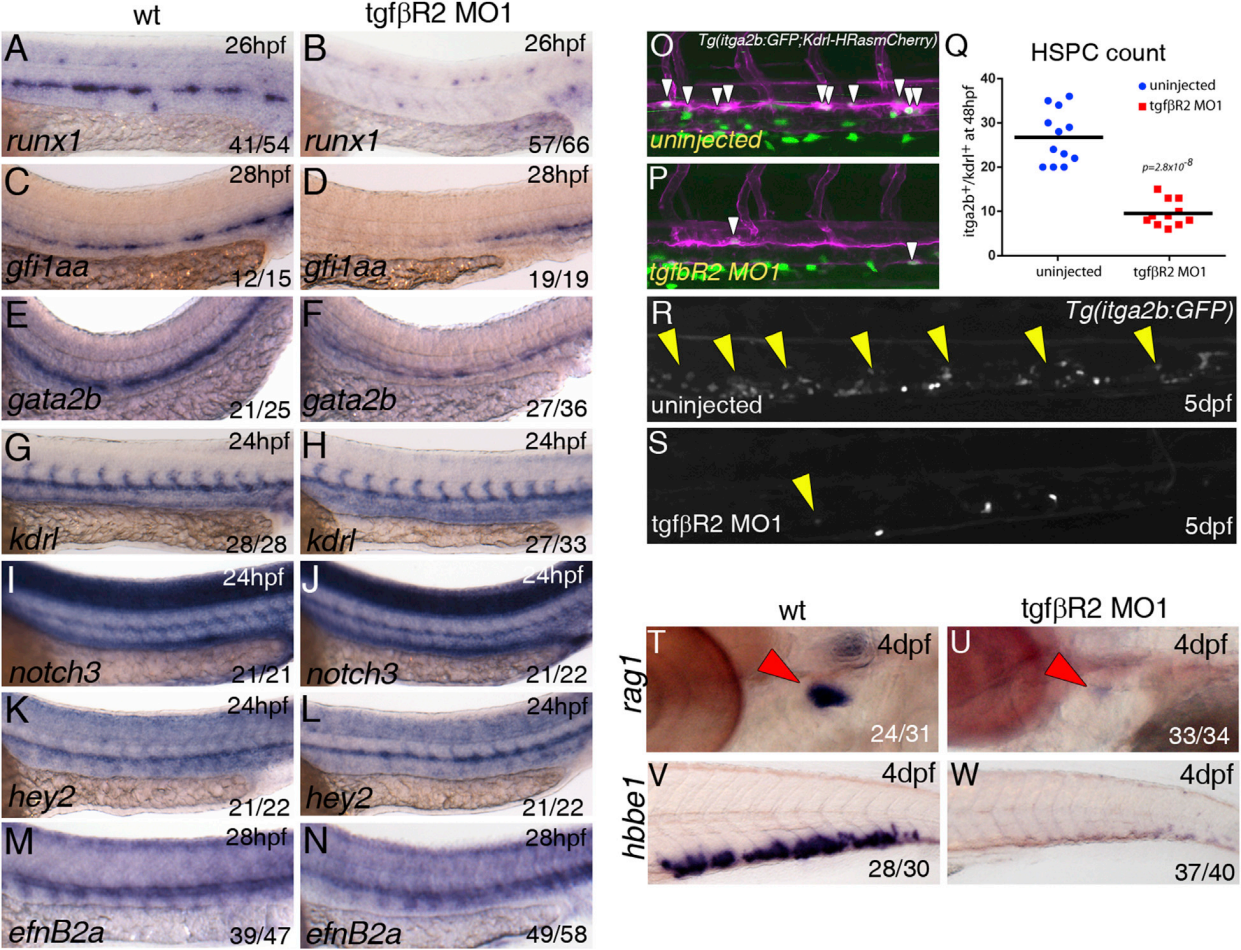
TGFβ Signaling Is Required for Specification of HSCs Expression of *runx1* in (A) wild-type (wt) and (B) *tgfbr2* morphants at 26 hpf. Expression of *gfi1aa* in (C) wild-type or (D) *tgfbr2* morphants at 26 hpf. Expression of *gata2b* in (E) wild-type or (F) *tgfbr2* morphants at 26 hpf. Expression of the vascular marker *kdrl* (G, H) and the arterial markers *notch3* (I, J), *hey2* (K, L), and *efnB2A* (M, N) is unaffected in *tgfbR2* morphants. Maximum projections of itga2b:GFP; Kdrl:HRas-mCherry transgenic embryos at 48 hpf in (O) uninjected and (P) *tgfbr2* MO1-injected embryos. Region shown includes part of the DA and white arrowheads denote itga2b:GFP^+^(green), kdrl:HRas-mCherry^+^ (magenta) HSPCs. (Q) HSPC counts in the entire trunk region of uninjected and *tgfbR2* morphant itga2b:GFP; Kdrl:HRas-mCherry transgenic embryos at 48 hpf (p value is indicated on the graph, n = 12 (wild-type) and n = 11 (*tgfbR2*^MO1^). itga2b-GFP^+^ cells are present in the CHT of itga2b:GFP embryos (R, yellow arrowheads) and greatly reduced in the CHT of *tgfbR2* morphants at 5 dpf (S). Expression of *rag1* in the thymus (red arrowheads) at 4 dpf in (T) wild-type and (U) *tgfbR2* morphants. Expression of *hbbe1* in the CHT at 4 dpf in (V) wild-type and (W) *tgfbR2* morphants. The numbers of embryos are shown in each panel as the number of embryos with phenotype/total number analyzed. See also Figure S2.

To investigate whether TGFβ signaling is required for the specification of HSPCs, we designed an antisense morpholino oligonucleotide (MO) targeting the start site of tgfbR2 translation (*tgfβR2*^MO1^; Figure S2A) and verified that it decreased TgfβR2 protein levels at 26 hpf (Figure S2B). *TgfbR2* morphants showed a severe decrease in expression of *runx1*, *gfi1aa*, and *gata2b*, two other HE markers (Butko et al., 2015, Cooney et al., 2013), at 26–28 hpf (Figures 2A–2F), suggesting that HSPC emergence is impaired. Specification of the arterial program in the endothelium of the DA by Notch signaling is required for HSPC emergence (Burns et al., 2005, Gering and Patient, 2005). Therefore, we asked if either the endothelial or the arterial programs are affected by loss of TGFβ signaling. We found that the pan-endothelial *kdrl* and the arterial markers *notch3, hey2*, and *efnb2a* were unaffected in *tgfβR2* morphants (Figures 2G–2N).

HSPCs emerging from the DA express *kdrl* and low levels of *itga2b* (also known as CD41) in itga2b:GFP;Kdrl:HsRas-mCherry transgenic embryos (Kissa et al., 2008). To quantitate the loss of HSPCs in *tgfbR2* morphants, we counted the number of Kdrl-mCherry^+^;itga2b-GFP^low^ HSPCs in the DA of live itga2b:GFP;Kdrl:HsRas-mCherry transgenic embryos by confocal microscopy at 48 hpf (Figures 2O–2Q). In *tgfbR2* morphants, the number of Kdrl-mCherry^+^;itga2b-GFP^low^ HSPCs was reduced more than 2-fold compared with uninjected embryos (Figure 2Q). The loss of itga2b-GFP^low^ cells in *tgfbR2* morphants was still evident at 5 dpf (Figures 2R and 2S) and, consistent with this, we found that the expression of the HSPC markers *runx1*, *cmyb*, and *ikzf1* was severely downregulated in *tgfbR2* morphants at 48 hpf (Figure S2D). *cmyb*, *ikzf1*, and *l-plastin* (pan-leukocyte marker) were severely reduced in the CHT and in the thymus at 4 dpf (Figures S2E and S2F). Expression of the early T cell marker *rag1* in the thymus (Figures 2T and 2U) and the erythroid marker *hbbe1* in the CHT (Figures 2V and 2W) were also severely reduced. Taken together, these experiments indicate that TGFβ signaling is required for the specification of HSPCs.

We designed a second MO, *tgfbR2*^MO2^ that blocks splicing of exon 4 of *tgfβR2* (Figure S2A), and confirmed the results obtained with *tgfbR2*^*MO1*^ (Figure S2G). Neither the pan-endothelial marker *fli1* nor the arterial markers *dll4* and *dlC* were affected in *tgfbR2*^MO1^ and *tgfbR2*^MO2^ morphants (Figure S2G). To examine whether TGFβ signaling is also required for primitive hematopoiesis, we performed in situ hybridization for *scl*, *gata1*, and *pu.1* at 20 hpf (Figure S2H) and *gata1* and *pu.1* at 24 hpf (Figure S2I). *TgfbR2* morphants showed no significant change in expression of these markers, suggesting that specification of primitive hematopoietic cells does not require TGFβ signaling through TgfβR2. However, maturation of primitive erythrocytes was slightly impaired, as suggested by a small decrease in *o*-dianisidine staining in *tgfbR2* morphants at 36 hpf (Figure S2J).

Taken together, our data show that TGFβ signaling through TgfβR2 is required for the specification of HSPCs independently of arterial programming.

### TGFβ1 in the Arterial Endothelium and TGFβ3 from the Notochord Are Required for HSPC Emergence

**Figure 3:**
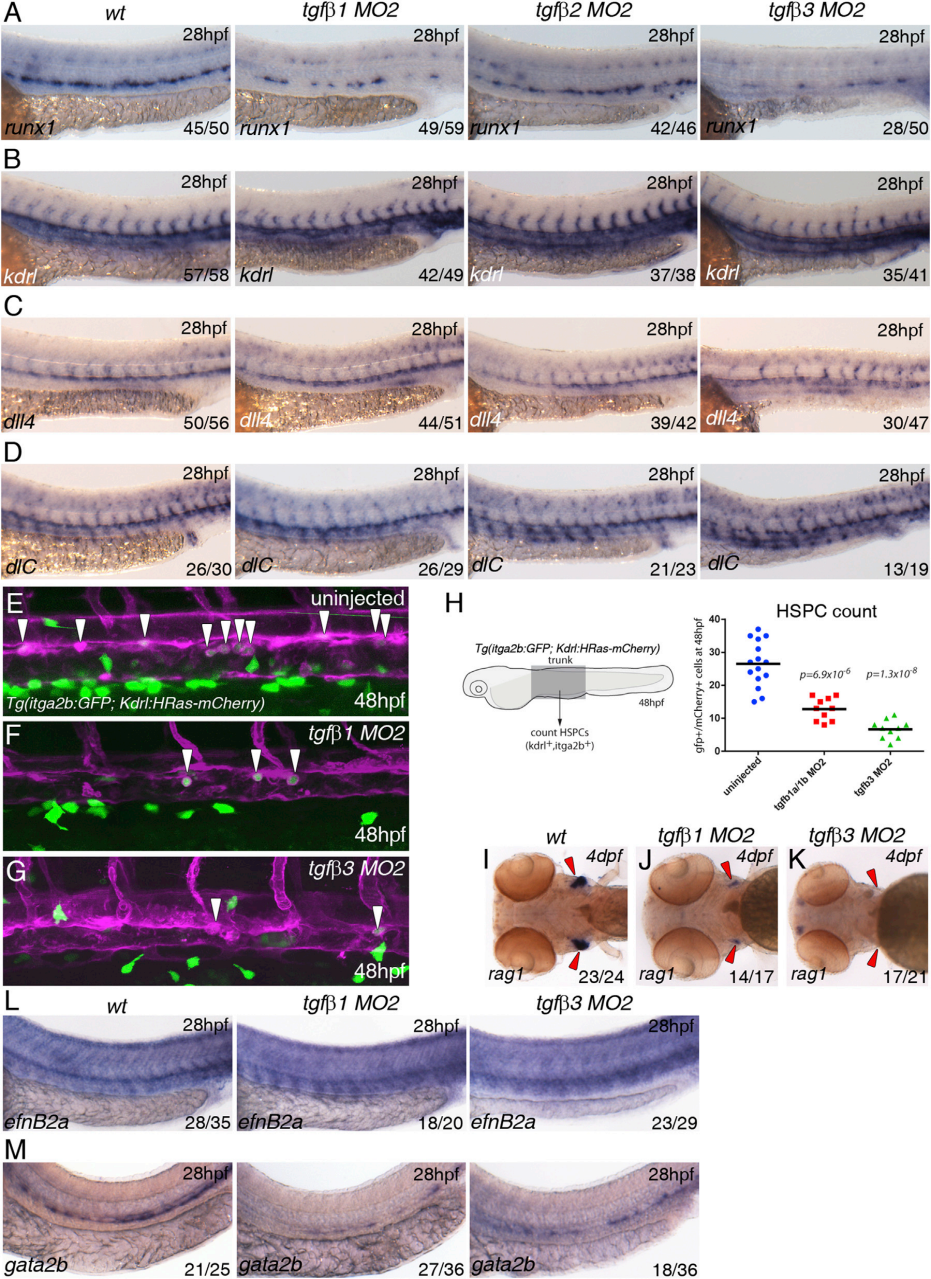
TGFβ1 and TGFβ3 Are Required for Specification of HSCs (A) Expression of *runx1* in wild-type, *tgfb1, tgfb2*, and *tgfb3* morphants. (B) Expression of *kdrl* in wild-type, *tgfb1, tgfb2*, and *tgfb3* morphants. (C) Expression of *dll4* in wild-type, *tgfb1, tgfb2*, and *tgfb3* morphants. (D) Expression of *dlC* in wild-type, *tgfb1, tgfb2*, and *tgfb3* morphants. All samples were analyzed at 28 hpf. (E–G) Maximum projections of itga2b:GFP; Kdrl:HRas-mCherry transgenic embryos in (E) uninjected, (F) *tgfb1* morphants, and (G) *tgfb3* morphants at 48 hpf. The images show part of the trunk DA and white arrowheads denote itga2b:GFP^+^ (green), kdrl:HRas-mCherry^+^ (magenta) HSPCs. (H) HSPCs counts in the entire trunk region of uninjected, *tgfb1*, and *tgfb3* morphant itga2b:GFP; Kdrl:HRas-mCherry transgenic embryos at 48 hpf (p value is indicated on the graph, n = 10 for each of the conditions). (I–K) Expression of *rag1* in the thymus at 4 dpf (red arrowheads) in (I) wild-type, (J) *tgfb1* morphants, and (K) *tgfb3* morphants. (L) Expression of *efnB2a* in wild-type, *tgfb1*, and *tgfb3* morphants. (M) Expression of *gata2b* in wild-type, *tgfb1*, and *tgfb3* morphants. The numbers of embryos are shown in each panel as the number of embryos with phenotype/total number analyzed. See also Figure S3.

Tgfβ1^−/−^ and TgfβR2^−/−^ mouse mutants share a similar vasculogenic phenotype in the yolk sac (Dickson et al., 1995, Oshima et al., 1996); thus we reasoned that TGFβ1 was the likeliest ligand for TgfbR2 in HSPC emergence. To test this hypothesis, we knocked down *tgfb1a* or *tgfb1b* with at least two splice-blocking morpholinos for each (Figures S3A–S3F) and found partial loss of *runx1* and *cmyb* in the DA without affecting expression of the arterial marker *dll4* (Figure S3F and data not shown). Co-injection of half the amounts of *tgfb1a*^*MO2*^ and *tgfb1b*^*MO2*^ (7.5 + 10 ng, respectively, referred to as *tgfb1*^*MO2*^) induced a severe loss of *runx1* and *cmyb* expression in a higher proportion of embryos at 28 hpf when compared with single *tgfb1a* or *tgfb1b* morphants (Figures 3A, S3E, and S3F; and results not shown), suggesting that, in single morphants, the TGFβ1 ligands can partially compensate for the other's absence and that both are required for HSPC emergence. Knocking down *tgfb2* with a splice-blocking morpholino (*tgfb2*^*MO3*^; Figure S3G) had very little effect on *runx1* expression (Figure 3A), whereas over half (28/50) of the *tgfb3* morphants (*tgfb3*^*MO2*^, Figure S3H) showed a severe decrease in *runx1* in the DA at 28 hpf (Figure 3A). Expression of *kdrl* in the endothelium and *dll4* and *dlC* in the arterial endothelium was unaffected in *tgfb1*^*MO2*^, *tgfb2*^*MO3*^, or *tgfb3*^*MO2*^ morphant embryos (Figures 3B–3D), consistent with TGFβ signaling being required for HSPC specification but not for arterial programming. To quantitate the effect, we counted the number of Kdrl-mCherry^+^;itga2b-GFP^low^ HSPCs at 48 hpf (Figures 3E–3H). Both *tgfb1*^*MO2*^ and *tgfb3*^*MO2*^ morphants showed severely reduced numbers of HSPCs when compared with uninjected embryos. *tgfb3*^*MO2*^ morphants had fewer HSPCs than *tgfb1*^*MO2*^ morphants at 48 hpf (Figure 3H), which correlated with a stronger decrease in *rag1* expression in the thymus of *tgfb3*^*MO2*^ morphants at 4 dpf (Figures 3I–3K). Further analysis revealed that expression of the arterial marker *efnB2a* was unaffected, whereas that of the HE marker *gata2b* was reduced in *tgfb1*^*MO2*^ and in half of the *tgfb3*^*MO2*^ embryos (Figures 3L and 3M). Next we investigated whether the milder phenotype in *tgfb3* morphants was due to upregulation of Tgfb1. We found that *tgfb3* was essentially absent in tgfb3^MO2^ morphants but *tgfb1a* or *tgfb1b* expression was unaffected (Figure S3I). Conversely, *tgfb1* morphants showed increased *tgfb3* expression in the notochord (Figure S3I). Knocking down *tgfb1* in *tgfb3* morphants increased the percentage of embryos with reduced *runx1* expression from 50% to 85% (Figures S3J and S3K), suggesting that Tgfβ1 and Tgfβ3 have an additive effect on HSPC specification. Taken together, we conclude that Tgfβ1a/1b produced by the ECs of the DA are required for HSPC formation by programming the HE downstream or in parallel to arterial programming. In addition, there is a significant paracrine contribution by Tgfβ3, which becomes a more important regulator of HSPC generation between 28 and 48 hpf.

### Vegf Signaling Regulates Expression of the *tgfb1a* and *tgfb1b* Ligands in the Dorsal Aorta

**Figure 4:**
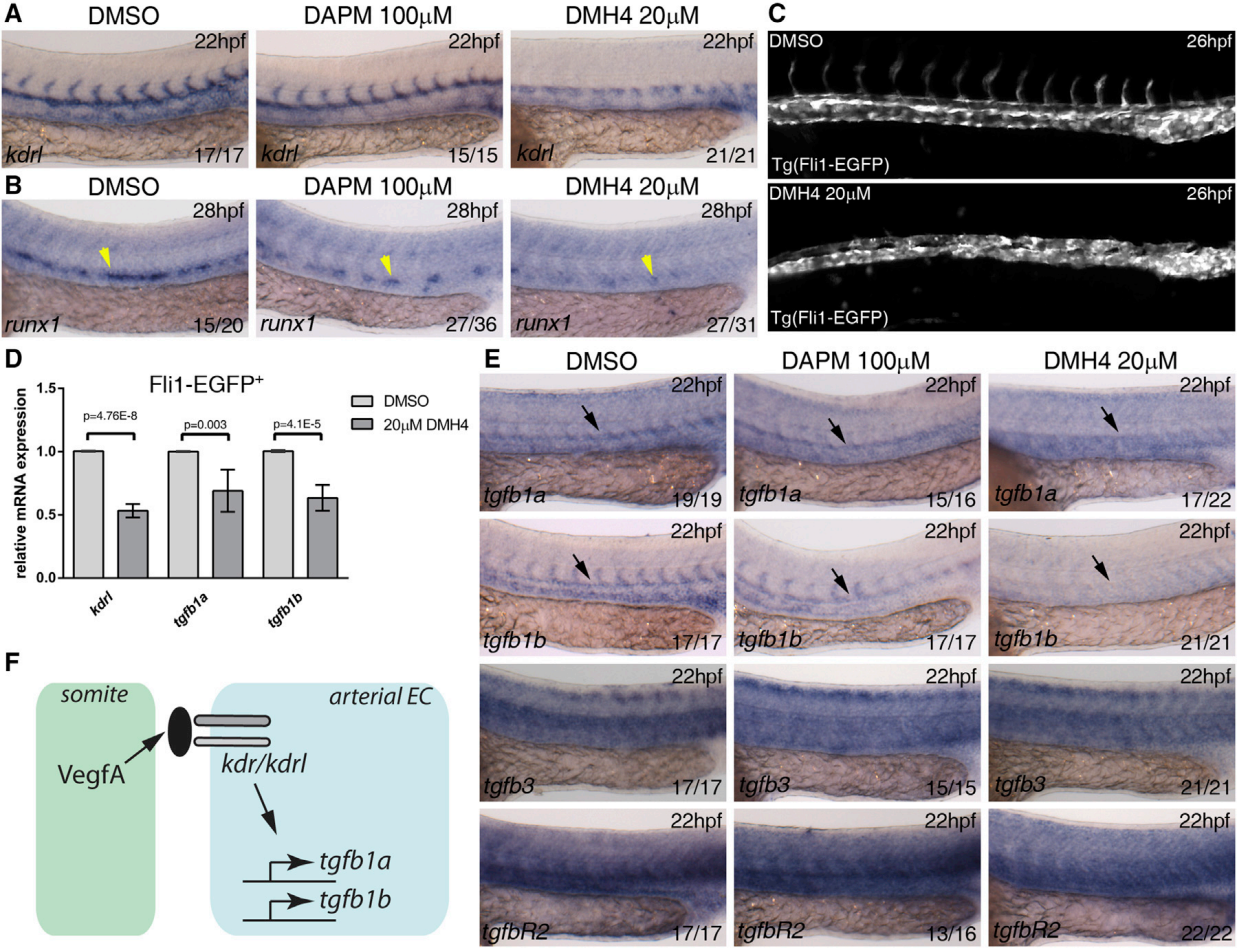
Vegf Signaling Is Required for *tgfb1a* and *tgfb1b* Expression in the Dorsal Aorta (A and B) Wild-type embryos were treated from 10 hpf with DMSO (control), Vegf inhibitor DMH4 (20 μM), and Notch inhibitor DAPM (100 μM) and collected at 22 hpf or 28 hpf. Embryos were collected and analyzed for (A) *kdrl* expression at 22 hpf and (B) *runx1* expression at 28 hpf. (C) Tg(Fli1:EGFP) embryos were treated from 10 to 26 hpf with DMSO or DMH4 (20 μM). DMH4-treated embryos showed a severe loss of intersomitic vessels but ECs are still present in the trunk, and circulation was detected in a majority of embryos at 48 hpf (data not shown). (D) Expression of *kdrl*, *tgfb1a*, *tgfb1b* by qPCR in 26 hpf sorted Fli1:EGFP^+^ ECs. All three genes were downregulated after DMH4 treatment. Results are shown as averages ± SD of 4–5 biological replicates. (E) Wild-type embryos were treated from 10 hpf with DMSO (control), Vegf inhibitor DMH4 (20 μM), and Notch inhibitor DAPM (100 μM) and collected at 22 hpf for analysis of *tgfb1a*, *tgfb1b*, *tgfb3*, and *tgfbR2* by in situ hybridization at 22 hpf. (F) Schematic representation of the experimental results. Black arrows indicate the location of the DA; yellow arrowheads indicate the location of *runx1* expression in the floor of the DA. The numbers of embryos are shown in each panel as the number of embryos with phenotype/total number analyzed. Arterial EC, arterial endothelial cell. See also Figure S4.

The sequential activity of VegfA and Notch is required for programming the DA endothelium to become arterial and give rise to HSPCs (Burns et al., 2005, Gering and Patient, 2005, Leung et al., 2013). Because our data suggest that the requirement for TGFβ lies downstream or parallel to arterial programming by Notch signaling (Figures 1, 2, and 3), we asked whether Vegf or Notch signaling might act as upstream transcriptional regulators of TGFβ ligands. To address this, we treated wild-type embryos after gastrulation with selective inhibitors for Vegf (DMH4, 20 μM) and Notch signaling (DAPM, 100 μM) (Hao et al., 2010, Walsh et al., 2002) and examined the expression of *tgfb1a*, *tgfb1b*, *tgfb3*, and *tgfbR2* (Figure 4). DMH4-treated embryos failed to form intersomitic vessels as expected (Bahary et al., 2007) and showed diminished *kdrl* expression in the trunk vasculature when compared with DMSO-treated controls (Figure 4A). The DAPM treatment had no effect on *kdrl* expression (Figure 4A). Blocking either Vegf or Notch signaling led to loss of *runx1* from the floor of the DA by 28 hpf, as described (Burns et al., 2005, Gering and Patient, 2005, Lam et al., 2010) (Figure 4B). To ask whether the loss of *kdrl* expression upon inhibition by DMH4 was due to transcriptional regulation by Vegf, we repeated the experiment in Tg(Fli1:EGFP) embryos. We confirmed that intersomitic vessels were absent but trunk ECs were still present in DMH4-treated embryos (Figure 4C). Analysis by qPCR showed that *kdrl* was decreased in DMH4-treated Fli-EGFP^+^ ECs (Figure 4D). Strikingly, inhibition by DMH4 led to decreased *tgfb1a* and *tgfb1b* in the endothelium, whereas DAPM treatment had no obvious effect (Figures 4D and 4E). *Tgfb3* and *tgfbR2* were unaffected by either treatment, suggesting that only *tgfb1a* and *tgfb1b* are Vegf-dependent. These results were confirmed by morpholino knockdown of the Vegf receptors, *kdr* and *kdrl* (Figure S4A). Next we asked whether Wnt16 and BMP4, which are required for HSPC formation independently of Vegf or Notch signaling in the endothelium (Clements et al., 2011, Wilkinson et al., 2009), could be upstream regulators of TGFβ. Knocking down either Wnt16 or BMP4 had no effect on TGFβ ligand or receptor expression (Figure S4B). Thus, we conclude that Vegf signaling is an upstream regulator of TGFβ signaling by positively regulating expression of *tgfb1a* and *tgfb1b* in ECs (Figure 4F) before HSPC specification.

### The Notch Ligand Jag1a Is a Downstream Target of TGFβ Signaling in Endothelial Cells

**Figure 5:**
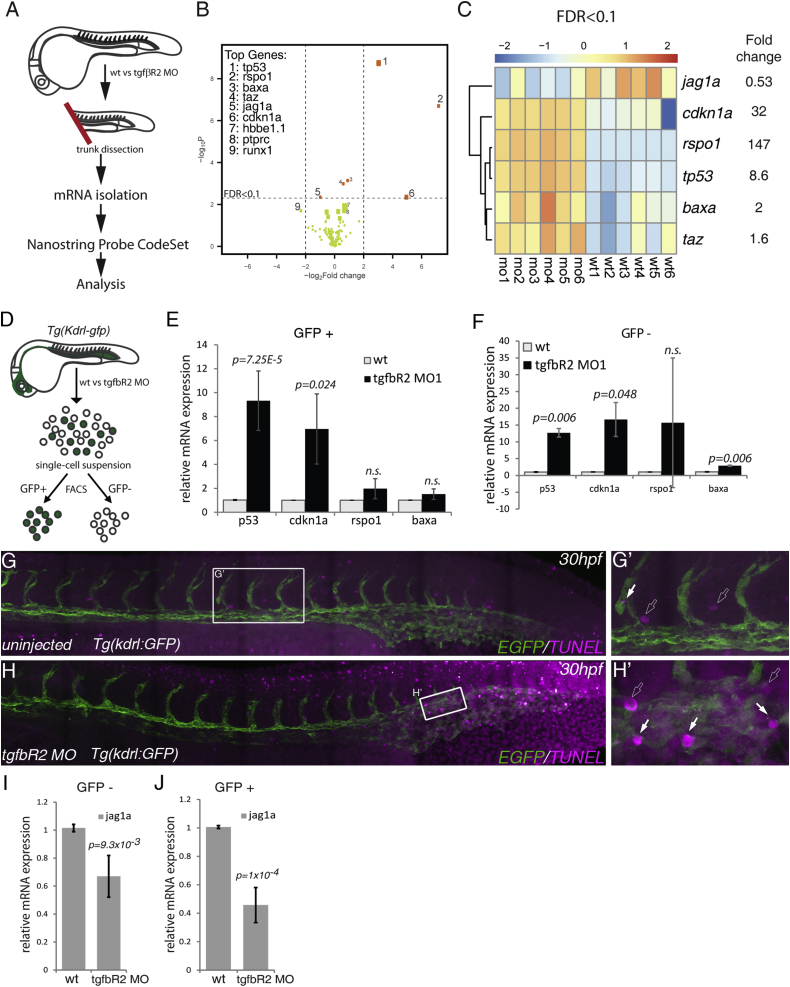
Multiplex Analysis of Gene Expression Shows that *jag1a* Is a Downstream Target of TGFβ Signaling (A) Schematic representation of the trunk dissection experiment for isolation of mRNA for hybridization with the NanoString Probe CodeSet. Six groups of independent wild-type (wt1–6) and *tgfbR2*^*MO1*^-injected embryos (mo1-6) were used in this analysis. (B) Volcano plot depicting differential gene expression between wild-type and *tgfbR2* morphants in log2-fold change with a significance level of p < 0.05. Vertical broken lines limit the absolute logFC larger than 0.5-fold change range, whereas the horizontal broken line represents the false discovery rate (FDR) threshold set at FDR < 0.1. The genes where FDR < 0.1 are shown as orange dots. The size of the dots is proportional to mRNA expression levels. (C) Hierarchical clustering of genes expressed with FDR < 0.1 in each of the six biological replicates analyzed (wild-type, wt1 to wt6; *tgfbR2*^*MO1*^, mo1 to mo6). Results are normalized and presented as *Z* scores from −2 (downregulated) to 2 (upregulated). (D) Schematic representation of the sorting of kdrl:GFP^+^ cells in wild-type and *tgfbR2* morphants (MO) by fluorescence-activated cell sorting to isolate mRNA and validate the NanoString results by qPCR. (E and F) qPCR of *p53*, *cdkn1a*, *rspo1*, and *bax* in (E) kdrl:GFP^+^ cells and (F) kdrl:GFP^−^ cells of wild-type and *tgfbR2* morphants (MO) at 28 hpf. *Taz* was omitted from the analysis as its fold induction <2. (G and G′) TUNEL-stained apoptotic cells in uninjected (control) kdrl:GFP embryos at 30 hpf. (H and H′) Apoptotic cells in *tgfbR2*^*MO1*^-injected kdrl:GFP embryos at 30 hpf. White arrows, apoptotic endothelial cells; outline arrows, apoptotic non-endothelial cells. (I and J) qPCR for *jag1a* in (I) kdrl:GFP^−^ cells and (J) in kdrl:GFP^+^ cells at 28 hpf. See also Figure S5 and Table S1.

Formation of HSPCs requires many cell extrinsic and intrinsic factors (ligands, receptors, transcription factors, and chromatin modifiers). Thus, to investigate whether any of the known pathways required to specify HSPCs are regulated by TGFβ signaling, we used the NanoString gene quantitation system (Geiss et al., 2008). We designed a custom panel of 132 NanoString probes that included Vegf, Notch, BMP, Wnt, Hh, and TGFβ signaling pathway components or targets. The probe set also contained known blood and endothelial genes, cell-cycle and apoptosis genes, mediators of EMT, and six housekeeping genes for data normalization. To assess expression changes in the somites as well as in the endothelium, we dissected the trunks of wild-type and *tgfbR2* morphant embryos at 26 hpf and isolated total RNA to hybridize against the NanoString probe set (Figure 5A). Only nine of the genes probed showed statistically significant differences in expression (p < 0.05 and an absolute logFC >0.5) between wild-type and *tgfbR2* morphants, importantly including decreased *runx1* expression in the morphants (Figure 5B and Table S1). Applying a more stringent filtering (false discovery rate <0.1) yielded a smaller high-confidence subset of differentially expressed genes in *tgfbR2* morphants (Figure 5C). Five of six genes in this subset were upregulated and three of those, *p53*, *cdkn1a*, *bax*, are associated with apoptosis and cell-cycle arrest (Menendez et al., 2009). A fourth gene, *rspo1*, is an agonist of Wnt signaling that is required for sprouting angiogenesis (Gore et al., 2011) and is expressed at very low levels in wild-type embryos (Table S1). *Taz*, a Wnt signaling mediator (Azzolin et al., 2012), was also upregulated in our assay, suggesting a link between TGFβ and Wnt signaling. However, when we sorted kdrl:GFP^+^ ECs versus kdrl:GFP^−^ cells from control embryos and *tgfbR2* morphants (Figure 5D), we found no significant difference in *rspo1* expression by qPCR in either population (Figures 5E and 5F). Analysis of *p53*, *cdkn1a*, and *bax* expression by qPCR showed that only *p53* and *cdkn1a* were significantly upregulated in ECs (Figure 5E), whereas all three were upregulated in kdrl:GFP^−^ cells (Figure 5F). These results suggested that *tgfbR2* morphants might show increased apoptosis. Thus we performed a TUNEL assay for apoptotic cells in Kdrl:GFP transgenic embryos and found a marked increase in TUNEL^+^ (apoptotic) cells in the trunk and tail regions of 30 hpf *tgfbR2* morphants compared with control embryos (Figures 5G and 5H). We found increased apoptosis in ECs in the tail vascular plexus (Figure 5H′, white arrows) but not in the trunk vasculature (Figure 5H) where HSPCs arise. Thus, if *p53* and its targets *cdkn1a* and *bax* play a role in HSC specification downstream of TGFβ, it appears to be independent of their pro-apoptotic activity. The increase in *p53* could have been non-specific due to the injection of MOs, as previously reported (Robu et al., 2007). However, knocking down *runx1* also led to an increase in *p53*, *cdkn1a*, and *baxa* (Figure S5K). This raises the possibility that the increase in pro-apoptotic gene expression in *tgfbR2* morphants could be indirect, acting downstream of Runx1.

Strikingly, *jag1a* was the only gene besides *runx1* that was significantly downregulated in *tgfbR2* morphants (Figures 5B and 5C). Neither its paralog *jag1b* nor any of the other Notch ligands or receptors in the probe set were significantly affected by loss of TGFβ signaling (Figure S5A). To confirm that *jag1a* was downregulated in the absence of TGFβ signaling, we injected *tgfbR2*^*MO1*^ into Kdrl:GFP embryos, sorted GFP^+^ ECs and GFP^−^ cells, and assayed *jag1a* expression by qPCR. *jag1a* was downregulated in both populations (Figures 5I and 5J). *dll4* and *gata2a* expression was unaltered in ECs from *tgfbR2* morphants (Figure S5L), confirming the NanoString results. To determine which TGFβ ligand regulates *jag1a*, we assayed its expression in *tgfb1*^*MO2*^ and *tgfb3*^*MO2*^ morphants compared with wild-type embryos at 26 hpf. *Jag1a* was downregulated in both t*gfb1*^*MO2*^ and *tgfb3*^*MO2*^ morphants (Figure S5M). Moreover, t*gfb1*^*MO2*^ and *tgfb3*^*MO2*^ morphants showed increased *p53* and no effect on *gata2a* or *dll4* expression (Figure S5N). Thus, our data indicate that *jag1a* is a TGFβ target in the endothelium at the onset of HSPC specification and suggest that both TGFβ1 and TGFβ3 contribute to the expression of *jag1a*.

### Jag1a Is Required Downstream of TGFβ to Program the HE

**Figure 6:**
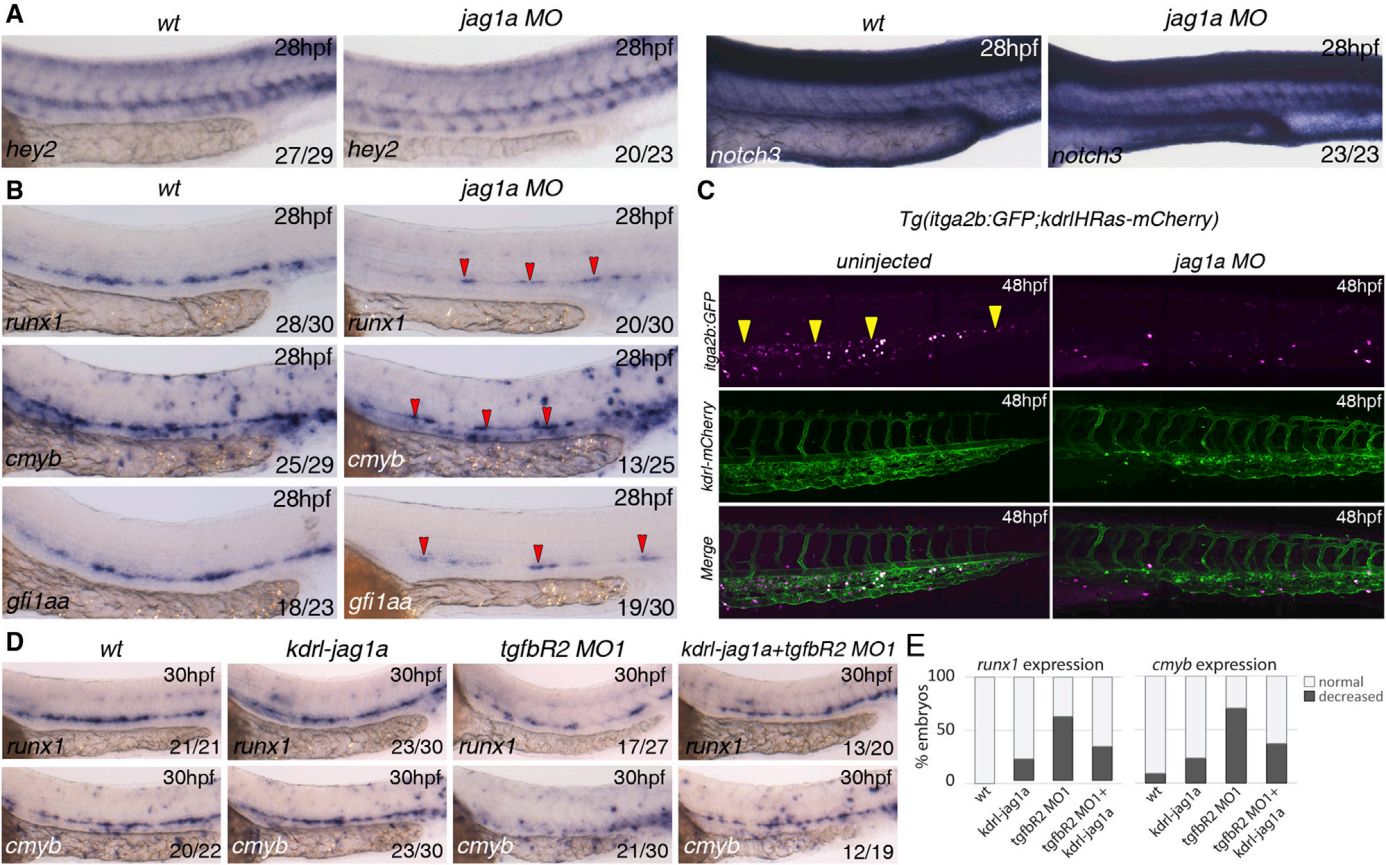
Jag1a Is Required Downstream of TGFβ Signaling for HSC Specification (A) Expression of arterial markers in *jag1a*^*MO*^ is unaffected when compared to wild-type embryos. (B) Expression of *runx1*, *cmyb*, and *gfi1aa* in wild-type and *jag1a* morphants (MO) at 28 hpf. All the markers analyzed are reduced or absent in *jag1a* morphants. Red arrowheads indicate the remaining gene expression in the floor of the DA of *jag1a* morphants. (C) HSPCs (yellow arrowheads) are severely reduced in the CHT of itga2b:GFP;Kdrl:HsRas-mCherry transgenic embryos at 48 hpf injected with the *jag1a*^*MO*^. Itga2b:GFP^+^ cells, magenta; Kdrl-HsRas:mCherry^+^ cells, green. (D) Overexpression of *jag1a* with a Kdrl:jag1-V5 construct partially rescues the loss of *runx1* and *cmyb* in the floor of the DA. 15 pg of the construct was used for this experiment. The numbers of embryos are shown in each panel as the number of embryos with phenotype/total number analyzed. (E) Quantitation of the rescue effect observed in (D). See also Figure S6.

To determine if Jag1a is required for arterial programming or HSPC specification in zebrafish, we knocked down *jag1a* with a specific morpholino (Yamamoto et al., 2010) and found no obvious defects in arterial programming compared with wild-type embryos (Figure 6A). However, expression of the HSPC markers *runx1*, *cmyb*, and *gfi1aa* was severely downregulated in *jag1a* morphants at 28 hpf (Figure 6B). Furthermore, itga2b:GFP^+^ HSPCs were nearly absent in the CHT of *jag1a* morphants by 48 hpf (Figure 6C) suggesting that the HE was mis-programmed and failed to give rise to HSPCs in the absence of Jag1a. A recent study showed that *jag1a* is regulated by TNFα through the TnfR2 receptor in ECs (Espin-Palazon et al., 2014). Expression of tnfR2 in ECs was unaffected in *tgfbR2* morphants (Figure S6), suggesting that TGFβ does not regulate *jag1a* indirectly via regulation of *tnfr2*. To determine if Jag1a is the main target of TGFβ signaling in HSPC specification, we restored Jag1a expression specifically in the endothelium of *tgfbr2*^*MO1*^ morphants using a Kdrl:jag1a construct. Wild-type embryos overexpressing *jag1a* in ECs showed little effect on expression of *runx1* or *cmyb* at 28 hpf (Figures 6D and 6E). However, forced expression of *jag1a* in the endothelium of *tgfbR2* morphants rescued the loss of *runx1* and *cmyb* expression (Figures 6D and 6E), confirming that the hematopoietic defects in *tgfbR2* morphants are mainly due to loss of *jag1a*. We conclude that autocrine TGFβ1 and paracrine TGFβ3 signal to the endothelium through TgfβR2, inducing *jag1a* expression, which in turn induces HE programming and HSPC emergence.

## Discussion

### TGFβ Is a Regulator of HSPC Specification in the Embryo

We have demonstrated a critical role for TGFβ signaling in the specification of HSPCs. Our data show that knockdown of the type II receptor for TGFβ leads to the loss of HSPCs and their differentiated progeny. Cell-autonomous Notch signaling is required for the programming of arterial identity in the endothelium (Quillien et al., 2014) and failure to acquire this identity, through the absence of Notch signaling or Hey2, leads to loss of HSPCs (Gering and Patient, 2010, Kim et al., 2014, Rowlinson and Gering, 2010). However, recent publications suggest that arterial identity is not an absolute requirement for HE specification and HSPC emergence (Ditadi et al., 2015, Jang et al., 2015). Here we show that neither Hey2 nor the Notch pathway components that program the artery are affected by the absence of TGFβ signaling, whereas the HE markers *gata2b*, *runx1*, and *gfi1aa* are strongly downregulated. Thus, we propose that TGFβ functions independently of arterial development to program the arterial ECs to become hemogenic.

### Parallel Activation of Notch and TGFβ Signaling by Vegf Programs the HE

Vegf and TGFβ are important regulators of vasculogenesis and angiogenesis in both embryonic development and cancer progression (Holderfield and Hughes, 2008), and crosstalk between them has been demonstrated, mainly through regulation of *vegfA* by TGFβ (Massague and Gomis, 2006). Here we show the opposite: expression of *tgfb1a* and *tgfb1b* ligands is dependent on VegfA signaling through its receptors Kdr and Kdrl. Vegf also regulates the expression of *hey2, notch3*, and *notch1b*, which are required for arterial programming (Gering and Patient, 2005, Lawson et al., 2002, Rowlinson and Gering, 2010). Thus, we propose that HSPC emergence requires parallel activation of both pathways by Vegf, where Notch signaling provides the arterial identity and TGFβ programs the endothelium to become hemogenic.

### TGFβ and Notch Crosstalk in EHT: Similarities to Epithelial-to-Mesenchymal Transition

Because *tgfbR2* is expressed in the DA prior to HSC specification (Figures S1 and S2), we propose that TGFβ ligands act directly on ECs, resulting in *jag1a* activation. Jag1a then activates the Notch receptor, presumably Notch1a (Espin-Palazon et al., 2014), and the signal-receiving cell becomes hemogenic by expressing specific markers such as *gata2b*, *runx1*, and *gfi1aa*. Loss of TGFβ signaling would therefore prevent HE from being specified by the Jag1a/Notch1a interaction. Thus, the concerted activities of TGFβ and Notch signaling explain how only some of the ECs in the floor of the aorta are programmed to become hemogenic. Interestingly, *jag1* expression is also induced by TGFβ prior to EMT and is required for epithelial cells to progress to the mesenchymal fate in oncogenic transformation (Zavadil et al., 2004). In development, the cardiac cushion arises by an endothelial-to-mesenchymal transition (EndoMT) and this is also dependent on crosstalk between TGFβ and Notch signaling (Lamouille et al., 2014). Thus, this crosstalk between TGFβ and Notch signaling is a shared feature between EMT, EndoMT, and EHT. The similarity between these processes may guide future studies on the molecular and cellular basis of EHT.

### TGFβ1a, TGFβ1b, and TGFβ3 Are Required Sequentially to Generate HSPCs

Genetic studies in mice suggested that paracrine TGFβ is primarily required to recruit smooth muscle cells to the endothelium (Pardali et al., 2010). In addition, autocrine signaling in ECs is important to regulate proliferation and migration (Pardali et al., 2010). Thus, TGFβ acts both in an autocrine and paracrine fashion in vivo. Similarly, here we describe two independent sources of TGFβ ligands that are required for HSPC specification: TGFβ1a and TGFβ1b in the endothelium, and TGFβ3 from the neighboring notochord. Our data suggest that TGFβ3 is less important for programming of HE but may instead play a more important role in the EHT process. In agreement with this, in situ hybridization for HSPC derivatives at 4 dpf showed a more severe phenotype in *tgfb3* morphants than in *tgfb1* morphants. That TGFβ3 has a role in hematopoiesis was surprising because mouse TGFβ3 mutants have no described hematopoietic phenotypes (Goumans and Mummery, 2000). However, TGFβ3^−/−^ mouse embryos show loss of palatal fusion due to defective EMT (Kaartinen et al., 1995, Proetzel et al., 1995). TGFβ3 induces EMT in palate epithelial cells by downregulating E-cadherin and upregulating fibronectin and vimentin (Nawshad et al., 2007). This raises the possibility that TGFβ may be required sequentially to generate HSPCs: TGFβ1 is required for the initial HE programming, and then TGFβ3 modulates expression of extracellular matrix components to allow HE cells to undergo EHT.

Knowledge of how ECs are programmed to become HSCs is critical to inform attempts to generate these cells in vitro for therapeutic purposes. Our findings show that TGFβ signaling is required to program the HE that will give rise to HSPCs. By contrast, we have previously shown that in *Xenopus laevis* excessive TGFβ signaling blocks specification of the hemangioblast population that precedes the formation of HE (Nimmo et al., 2013). Similarly, adding TGFβ2 to Pre-HPCs, a population of primitive hematopoietic precursor cells (Ve-Cad^+^, CD41^+^), impairs the EHT process in vitro (Vargel et al., 2016). This suggests that primitive hematopoiesis is sensitive to elevated levels of TGFβ signaling. Whether excessive TGFβ hinders EHT from the embryonic HE that gives rise to definitive HSPCs remains to be determined. Our work highlights the importance of identifying the different spatial and temporal requirements for TGFβ signaling in the formation of HSCs and will help to realize the goal of generating HSCs in vitro for regenerative medicine.

## Experimental Procedures

### Ethics Statement

All animal experiments were performed under a Home Office Licence according to the Animals Scientific Procedures Act 1986, UK, and approved by the local ethics committee.

### Fish Breeding and Maintenance

Wild-type, Tg(kdrl:GFP)^s843^ (Jin et al., 2005), Tg(itga2b:GFP)^la2^ (Lin et al., 2005), Tg(Kdrl-HsRas-mCherry)^s896^ (Bertrand et al., 2010), Tg(Fli1-GFP)y1Tg (Lawson and Weinstein, 2002), and Tg(cmyb:GFP)^zf169Tg^ (North et al., 2007) fish were bred, maintained, and staged as described (Westerfield, 2000). Tg(itga2b:gfp; Kdrl-HsRas-mCherry) animals were generated by natural mating.

### Morpholinos and RNA and DNA Injections

Antisense MOs (GeneTools) were used to target *runx1* (Gering and Patient, 2005), *tgfβ3* (*tgfb3*^*MO2*^) (Cheah et al., 2010), *kdr* + *kdrl* (Bahary et al., 2007), and *jag1a* (Yamamoto et al., 2010) at the amounts specified. The MOs selected for this study were *tgfbR2*^MO1^, *tgfb1a*^*MO2*^ *+ tgfb1b*^*MO2*^ (referred to as *tgfb1*^MO2^), *tgfb2*^*MO2*^, and *tgfb3*^*MO2*^ at the amounts indicated (see Supplemental Experimental Procedures). Typically, 1 nl total volume of MO was injected in 1–4 cell stage embryos. MO design and validation is described in the Supplemental Experimental Procedures.

To rescue the loss of HSC markers in *tgfbR2* morphants, we transiently expressed *jag1a* in ECs under the control of the *Kdrl* promoter (Jin et al., 2005) (see Supplemental Experimental Procedures). The amount of DNA used for the rescue experiment is shown in the figure legends.

### Western Blotting

Protein extracts were prepare as described (Link et al., 2006). TgfβR2 protein was detected by a primary anti-tgfβR2 antibody (diluted 1:250 in blocking solution, sc-17792; Santa Cruz Biotechnology) followed by a goat anti-mouse horseradish peroxidase (HRP)-conjugated secondary antibody (1:1,000 in blocking solution, P044701-2, DAKO). An anti β-actin-HRP-conjugated antibody (1:35,000, A3854; Sigma) was used for loading control.

### NanoString Expression Analysis

To quantitate the effects of *tgfβR2* loss of function in and around the embryonic DA, trunks from anesthetized 26–28 hpf embryos were microdissected with a straight stab knife. Total RNA was isolated with the RNEasy Micro kit (QIAGEN) following the manufacturer's instructions and quantified in a Nanodrop spectrometer. We interrogated expression of a panel of 132 probes (see Supplemental Experimental Procedures) using the NanoString nCounter gene expression system.

### mRNA Extraction, Flow Cytometry, cDNA Synthesis, and qPCR

Total RNA was isolated from wild-type or morpholino-injected embryos using TRI reagent (Sigma) and cleaned using the RNEasy Micro kit (QIAGEN) following the manufacturer's instructions. To interrogate gene expression in ECs of tgfβR2 morphants, uninjected and tgfβR2 MO1-injected Tg(kdrl:gfp) embryos were dissociated, and kdrl-GFP^+^ cells were isolated and processed for mRNA extraction with the RNEasy Micro kit (QIAGEN) as described (Monteiro et al., 2011). cDNA was synthesized from total RNA using a Superscript III RT-PCR enzyme (Invitrogen) following the manufacturer's instructions. The primers used for quantitative real-time PCR (qPCR) are shown in the Supplemental Experimental Procedures. Fold changes in gene expression were calculated using the 2^−ΔΔCτ^ method (Livak and Schmittgen, 2001) and normalized to a geometric mean of *bactin2* and *ef1a*.

### In Situ Hybridization, Sections, and Image Acquisition

Whole-mount in situ hybridization was carried out as described (Jowett and Yan, 1996). cDNA fragments for *tgfβR2*, *tgfβ1a*, *tgfβ1b*, and *tgfβ3* were PCR-amplified from 24 hpf embryo cDNA, cloned into pGEMT-Easy, and used as templates to generate in situ hybridization probes (see Supplemental Experimental Procedures). After in situ hybridization, embryos were processed and imaged as described (Gering and Patient, 2005, Monteiro et al., 2011).

### Fluorescence Imaging and Image Processing

HSPCs express low levels of a GFP transgene under the control of the itga2b promoter (Kissa et al., 2008). Itga2b:GFP^low^, kdrl:HsRas-mCherry^+^ HSPCs were imaged in uninjected and morpholino-injected Tg(itga2b:GFP; Kdrl:HsRas-mCherry) embryos at 48 hpf on a Zeiss LSM780 confocal microscope (Zen software). HSPCs were enumerated in maximum intensity projection images. GraphPad Prism software was used to generate scatterplots of cell counts and for statistical analysis. Alternatively, Tg(itga2b:GFP) embryos were imaged on a Zeiss Lumar V.12 stereomicroscope with an AxioCam MRm (Zeiss) and AxioVision software.

Apoptosis staining was performed with the Click-IT TUNEL Alexa 594 kit (C10246; Life Technologies) followed by immunostaining against GFP (see Supplemental Experimental Procedures).

Images were processed and figures and schemes were assembled in Adobe Photoshop CS5 and Adobe Illustrator CS5.

## Author Contributions

R.M. performed most experiments and analyzed the data; T.P., P.P., J.K., N.J., F.B., and A.K. performed experiments and analyzed the data; E.R. analyzed the NanoString data and performed statistical analysis; R.M. and R.P. conceived experiments, wrote the manuscript and secured funding.

## Supplemental Information

Document S1. Supplemental Experimental Procedures and Figures S1–S6

Table S1. Mean Expression Values of the Data Analyzed with the NanoStringNorm Package Sorted by Increasing p Value, Related to Figure 5

Document S2. Article plus Supplemental Information
