# Supplementary material for: Transforming Growth Factor β Drives Hemogenic Endothelium Programming and the Transition to Hematopoietic Stem Cells
Source: Dev Cell. 2016 Aug 22;38(4):358–70. doi: 10.1016/j.devcel.2016.06.024 (PMC4998007; doi:10.1016/j.devcel.2016.06.024)
Supplement: Document S1. Supplemental Experimental Procedures and Figures S1–S6 [file mmc1.pdf]

Developmental Cell, Volume *38*

Supplemental Information

Transforming Growth Factor β Drives Hemogenic Endothelium Programming and the Transition to Hematopoietic Stem Cells

Rui Monteiro, Philip Pinheiro, Nicola Joseph, Tessa Peterkin, Jana Koth, Emmanouela Repapi, Florian Bonkhofer, Arif Kirmizitas, and Roger Patient

Supplemental Information Inventory

Supplemental Figures S1-S6

Figure S1, related to Figure 1

Figure S2, related to Figure 2

Figure S3, related to Figure 3

Figure S4, related to Figure 4

Figure S5, related to Figure 5

Figure S6, related to Figure 6

Supplemental Table S1 (excel file), related to Figure 5

Supplemental Experimental Procedures, related to Figures 1-6, S1-S6

Supplemental References, related to Supplemental Experimental Procedures and to Supplemental Table S1

Supplemental Figures and Figure Legends

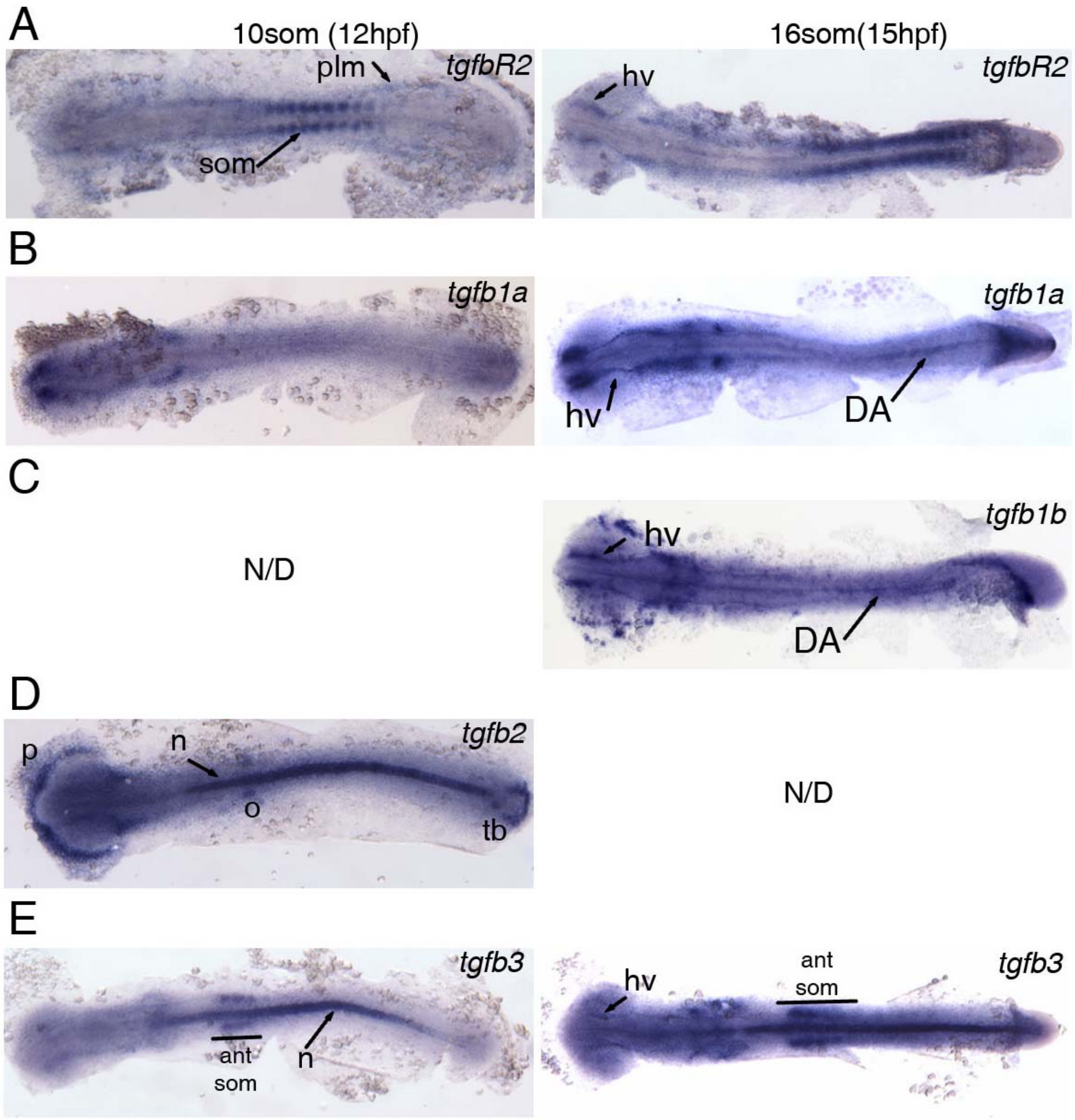

Figure S1, related to Figure 1 – Expression of TGFβ signalling components at 12hpf (10-somite stage) and 15hpf (16-somite stage). All embryos were flatmounted for imaging. (A) Expression of *tgfbR2* in the somites (som) and posterior lateral mesoderm (plm) at 12hpf. Expression of *tgfbR2* at 15hpf in head vasculature (hv) and somites. (B) *tgfb1a* is not expressed in the posterior at 12hpf but is present in head vasculature from 12hpf and in the embryonic dorsal aorta (DA) at 15hpf. (C) *tgfb1b* is also present in the head vasculture and DA at 15hpf. Note that there is anterior expression of *tgfb1b* in cells that are likely myeloid. (D) *tgfb2* is expressed in the polster (p), otic vesicles (o), notochord (n) and in the tailbud (tb) at 12hpf. (E) Expression of *tgfb3* in the notochord and in the 4 anterior-most somites (ant som) at 12hpf. At 15hpf, the expression in the somites and notchord is maintained and weak expression in the head vasculature is observed.

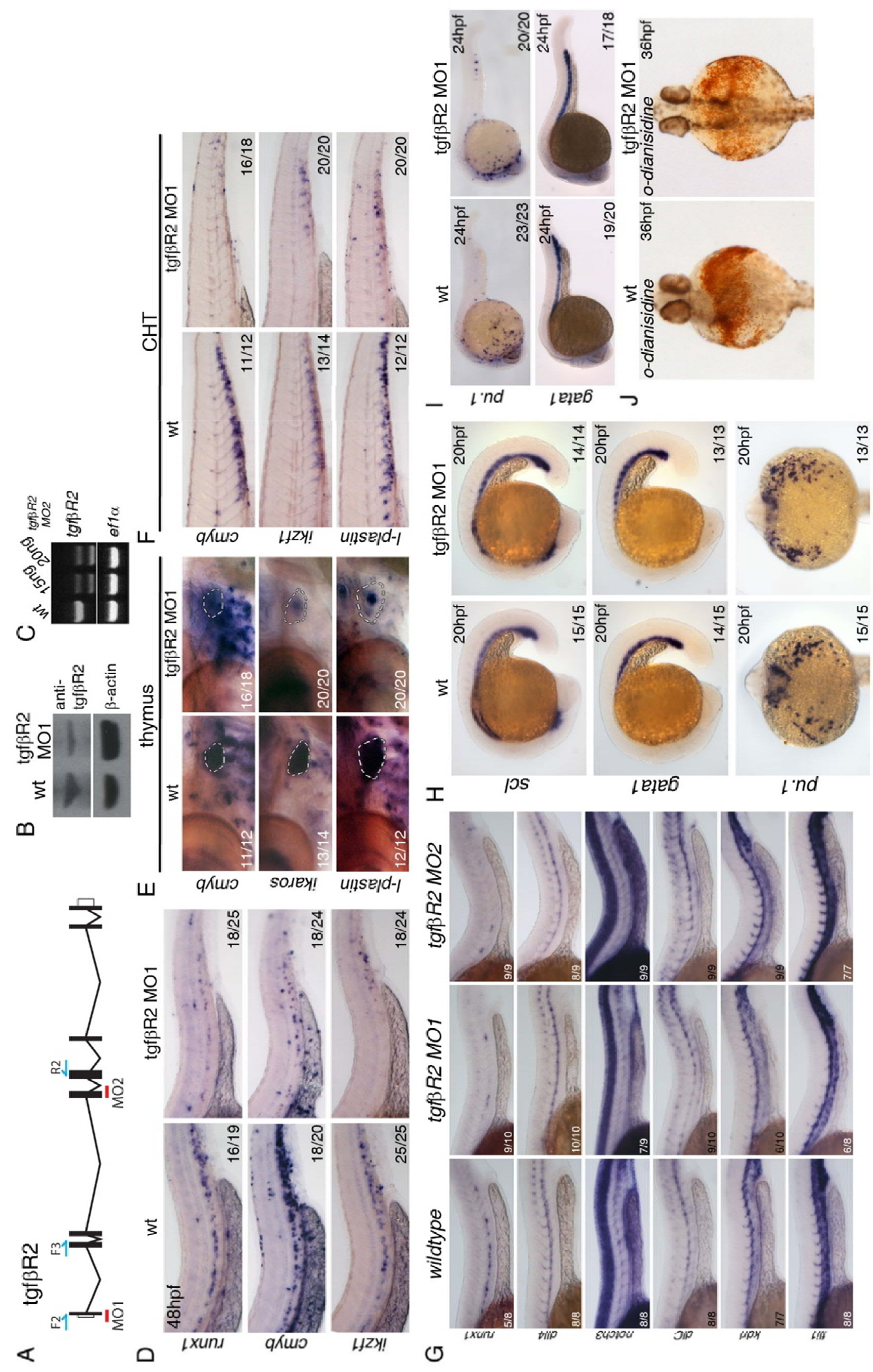

Figure S2, related to Figure 2 – Further characterization and validation of two independent morpholinos targeting tgfβR2 and analysis of primitive haematopoiesis in tgfβR2 morphants. (A) Schematic representation of the genomic organization of the tgfbR2 gene, location of morpholinos

and primers used to validate the morpholinos. (B) Western blot against TgfβR2 showing a decrease of WT protein induced by the *tgfbR2*^{MO1} morpholino. (C) Validation of the *tgfbR2*^{MO2} by qPCR on 24hpf cDNA with tgfbR2 F2/R2 primers (see below). Ef1a PCR was used as a control for the PCR. (D) Expression of *runx1*, *cmyb* and *ikzf1* at 48hpf is reduced in the trunk and CHT of *tgfbR2* morphants. Expression of *cmyb*, *ikzf1* and *l-plastin* is severely reduced (E) in the thymus and (F) in the CHT of *tgfbR2* morphants at 4dpf. (G) Characterisation of the tgfbR2 MO1 and MO2 morpholinos. *Runx1* expression is decreased upon injection of either morpholino, whereas the arterial markers dll4, notch3 and dlC and the vascular markers kdrl and fli1 are grossly normal. (H) Expression of *scl*, *gata1* and *pu.1* is indistinguishable between wildtype and *tgfbR2* morphant embryos at 20hpf. (I) Expression of the primitive hematopoietic markers *gata1* and *pu.1* is unaffected in *tgfbR2* morphants at 24hpf. (J) *tgfbR2* morphants show a slight reduction in o-dianisidine staining at 36hpf. Numbers of embryos analysed are shown in each panel as number of affected embryos/total observed.

Figure S3

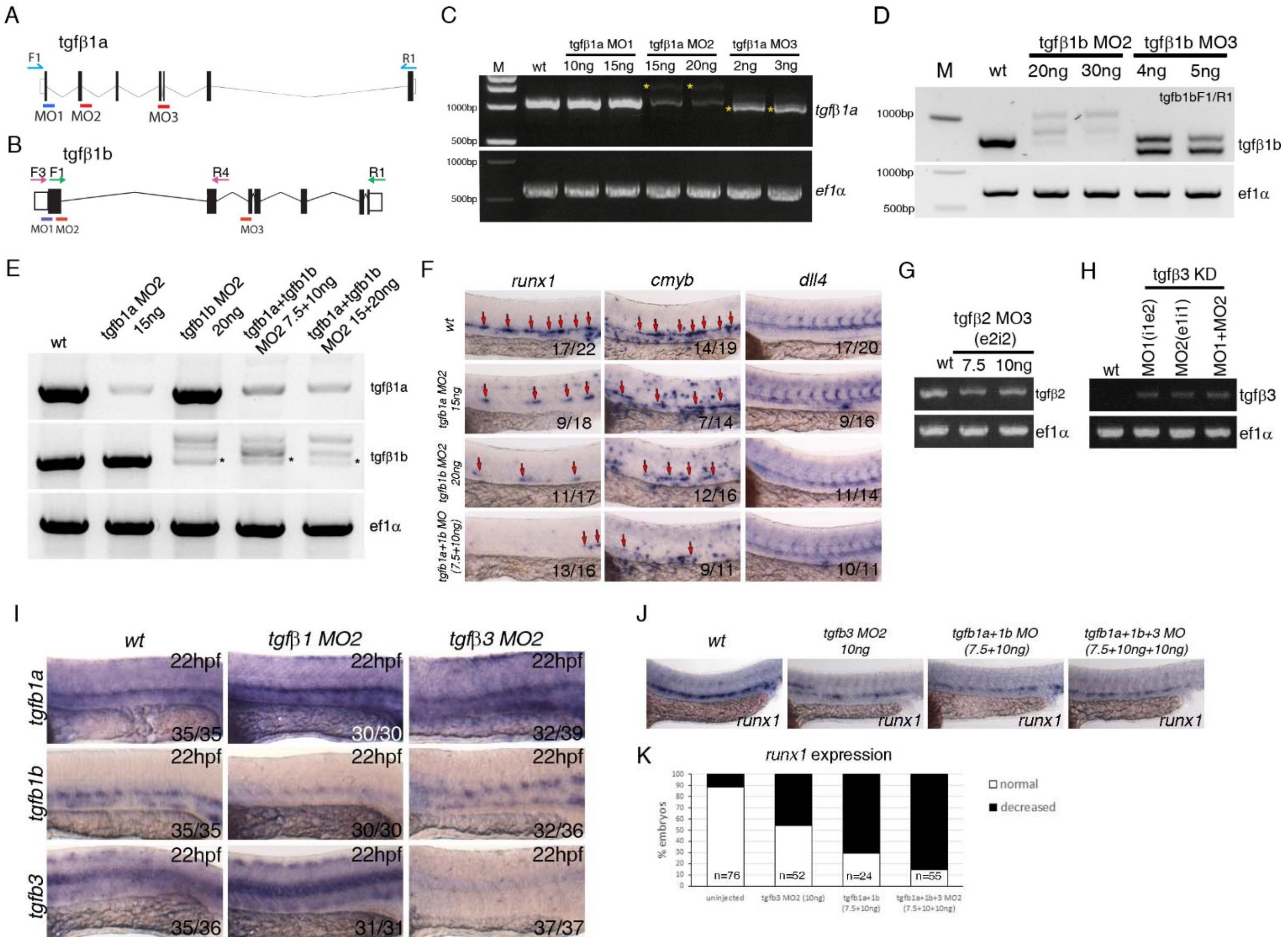

Figure S3, related to Figure 3 – Validation of morpholinos targeting *tgfβ1a*, *tgfβ1b*, *tgfβ2* and *tgfβ3* and further characterization of the phenotype. (A) Schematic representation of the genomic organization of the *tgfb1a* gene, location of morpholinos and primers used to validate morpholino activity. (B) Schematic representation of the genomic organization of the *tgfb1b* gene, location of morpholinos and primers used to validate morpholino activity. (C) Validation of the activity of the *tgfb1a* morpholinos by PCR. Yellow asterisk marks PCR products generated as a result of aberrant splicing induced by the *tgfb1a* morpholinos MO2 and MO3. We have used *tgfb1a*^{MO2} in all of the subsequent analysis. (D) Validation by PCR of the activity of two antisense morpholinos targeting *tgfb1b*. Clear aberrant splicing was induced by *tgfb1b* morpholinos MO2 and MO3. We have used *tgfb1b*^{MO2} in all of the subsequent analysis. (E) Validation by PCR of the *tgfb1a*^{MO2}+*tgfb1b*^{MO2} combination. Asterisk marks the remainder of the normally spliced *tgfb1b* gene product. (F) Testing the *tgfb1a*+*tgfb1b* MO2 combinations by *in situ* hybridisation against HE markers *runx1*, HSPC marker *cmyb* and arterial marker *dll4* at 28hpf. The combination of *tgfb1a*+*tgfb1b* MO2 (referred to

in the main text as *tgfb1*^{MO}) was used at 7.5 ng/μl +10ng/μl throughout the manuscript. Numbers of embryos analysed are shown in each panel as number of affected embryos/total observed. (G) Validation by PCR of the activity of a morpholino targeting *tgfb2*. (H) Validation by PCR of the activity of two published morpholinos (Cheah et al, 2010) targeting *tgfb3*. *Tgfb3*^{MO2} was used in all of the subsequent analysis. (I) Expression of *tgfb1a*, *tgfb1b* and *tgfb3* in *tgfb1*^{MO2} and *tgfb3*^{MO2} at 22hpf. *tgfb1*^{MO2} morphants show an increase in *tgfb1a* and *tgfb3* and a decrease in *tgfb1b*; *tgfb3*^{MO2} morphants show a dramataic loss of *tgfb3* expression. Numbers of embryos analysed are shown in each panel as number of affected embryos/total observed. (J,K) Comparison and quantification of the runx1 expression in the DA upon single tgfb3^{MO2}, tgfb1^{MO2} or combined tgfb3^{MO2}+tgfb1^{MO2}, showing increased severity of the phenotype in the triple morphants as compared to single morphants. The numbers of embryos analysed are shown in the graph in (K).

Figure S4

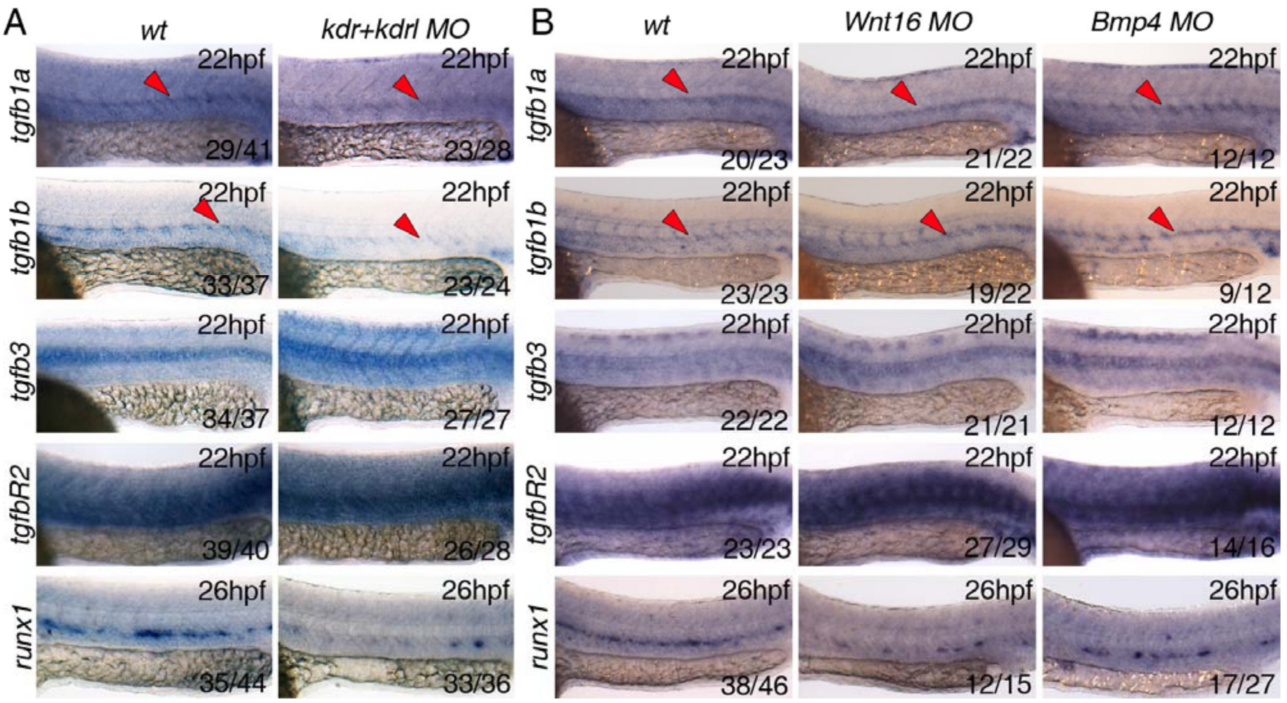

Figure S4, related to Figure 4 – Inhibition of Vegf (*kdr+kdrl*^{MO}), Wnt (*Wnt16*^{MO}) or BMP (*BMP4*^{MO}) signalling pathways by morpholino oligonucleotides. All *in situ* hybridisation experiments were performed at 22hpf except for the *runx1* probe, performed at 26hpf. (A) Loss of Vegf signalling upon morpholino knockdown of the Vegf receptors *kdrl* and *kdrl* (Bahary et al, 2007) results in decreased expression of *tgfb1a* and *tgfb1b* in the dorsal aorta (DA). *Runx1* expression in the DA was used as a positive control for the experiment. (B) Loss of Wnt signalling or BMP signalling by morpholino knockdown of Wnt16 (Clemens et al, 2011) or BMP4 (Chocron et al, 2007) showed no effect of expression of TGFβ ligands or receptor. *Runx1* expression in the DA was used as a positive control for the experiment. Numbers of embryos analysed are shown in each panel as number of affected embryos/total observed.

Figure S5

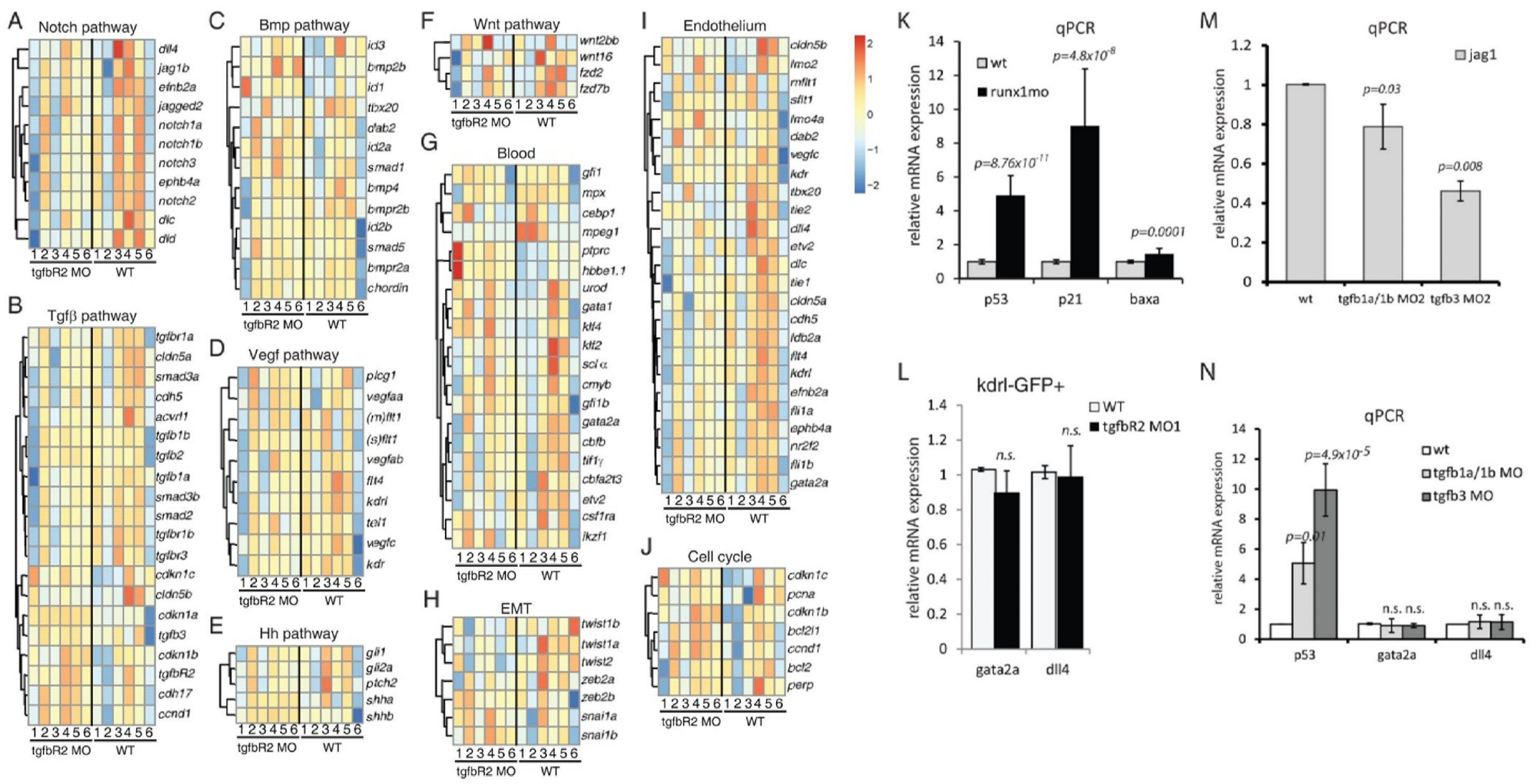
A Notch pathway
dll4
jag1b
efnb2a
jagged2
notch1a
notch1b
notch3
ephb4a
notch2
dlc
dld
1 2 3 4 5 6 1 2 3 4 5 6
tgfbR2 MO
WT
B Tgfβ pathway
tgfbr1a
cldn5a
smad3a
cdh5
acvrl1
tgfb1b
tgfb2
tgfb1a
smad3b
smad2
tgfbr1b
tgfbr3
cdkn1c
cldn5b
cdkn1a
tgfb3
cdkn1b
tgfbR2
cdh17
ccnd1
C Bmp pathway
id3
bmp2b
id1
tbx20
dab2
id2a
smad1
bmp4
bmpr2b
id2b
smad5
bmpr2a
chordin
D Vegf pathway
plcg1
vegfaa
(m)flt1
(s)flt1
vegfab
flt4
kdrl
tel1
vegfc
kdr
E Hh pathway
gli1
gli2a
ptch2
shha
shhb
F Wnt pathway
wnt2bb
wnt16
fzd2
fzd7b
G Blood
gfi1
mpx
cebp1
mpeg1
ptprc
hbbe1.1
urod
gata1
klf4
klf2
scl α
cmyb
gfi1b
gata2a
cbfb
tif1γ
cbfa2t3
etv2
csf1ra
ikzf1
H EMT
twist1b
twist1a
twist2
zeb2a
zeb2b
snai1a
snai1b
I Endothelium
cldn5b
lmo2
mflt1
sflt1
lmo4a
dab2
vegfc
kdr
tbx20
tie2
dll4
etv2
dlc
tie1
cldn5a
cdh5
ldb2a
flt4
kdrl
efnb2a
fli1a
ephb4a
nr2f2
fli1b
gata2a
J Cell cycle
cdkn1c
pcna
cdkn1b
bcl2l1
ccnd1
bcl2
perp
2
1
0
−1
−2
K qPCR
wt
runx1mo
relative mRNA expression
p=8.76x10^-11
p=4.8x10^-8
p=0.0001
p53
p21
baxa
L kdrl-GFP+
WT
tgfbR2 MO1
n.s.
n.s.
gata2a
dll4
M qPCR
jag1
p=0.03
p=0.008
wt
tgfb1a/1b MO2
tgfb3 MO2
N qPCR
wt
tgfb1a/1b MO
tgfb3 MO
p=4.9x10^-5
p=0.01
n.s. n.s.
n.s. n.s.
p53
gata2a
dll4

Figure S5, related to Figure 1 and Table S1 – Multiplex analysis of the gene expression profile in *tgfbR2* morphant embryos of a custom Probe Set (132 genes) using Nanostring (see Supplementary Experimental Procedures and Table S1). The custom designed probes include genes that belong to known pathway or processes that affect endothelial or hematopoietic cell programming, fate or survival, as well as six housekeeping genes. All genes shown here displayed no significant changes in expression upon $tgfbR2^{MO1}$ knockdown (see Figure 5 for differentially expressed genes). Each column represents one sample in a total of six wt and six $tgfbR2^{MO1}$ replicate morpholino injections (A) Notch pathway genes. (B) TGFβ pathway. (C) BMP pathway. (D) Vegf pathway. (E) Hedgehog pathway. (F) Wnt pathway. (G) Blood: genes expressed mostly in HSPCs, erythroid or myeloid cells. (H) Genes with known roles in epithelial to mesenchymal transition (EMT). (I) Gene expressed in endothelial cells (J) Cell cycle: genes that mark proliferation or apoptosis. Note that expression data is repeated for some genes as they fall into more than one category. (K) *p53*, *cdkn1a* and *baxa* are upregulated in *runx1* morphants. Trunks from 20 wild type and 20 *runx1* morphant embryos at 27hpf were dissected and the cDNA analysed by qPCR. The data represents the average of 4 biological replicates and were normalised to *gapdh*. (L) qPCR in kdrl-GFP^{+} endothelial cells confirms that neither *gata2a* nor *dll4* expression were affected by *tgfbR2* morpholino knockdown at 26hpf. (M) qPCR for *jag1a* in *tgfb1* and *tgfb3* morphants at 28hpf. (N) *p53* but not *gata2a* or *dll4* expression are affected in *tgfb1* (*tgfb1a* MO2+ *tgfb1b* MO2) or *tgfb3* morphants. qPCR results are shown as the average (±s.d.) of 3-6 biological replicates. Expression levels were normalized to *bactin2* and *ef1a*. *p* values are shown on the graphs. n.s.- not significant.

Figure S6

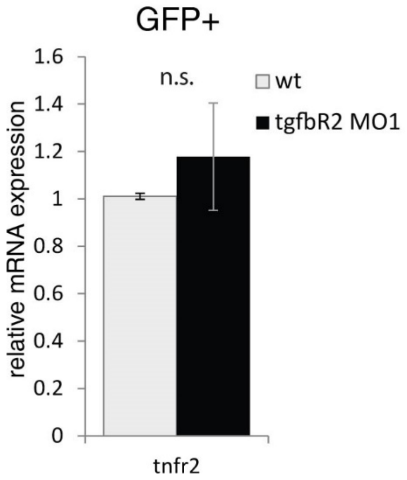

Figure S6, related to Figure 6 – qPCR analysis of *tnfr2* expression**.** *tnfr2* expression is unaffected in kdrl-GFP$^+$ endothelial cells at 26hpf in *tgfbR2* morphants. qPCR results are shown as the average (±s.d.) of 3 biological replicates; each biological replicate was done in triplicate. Expression levels were normalized to *bactin2* and *ef1a*. n.s.- not significant.

Supplemental Table S1 (excel file), related to Figure 5 - Mean Expression Values of the Data Analysed with the NanoStringNorm Package Sorted by Increasing P Value. The Top 9 Genes ($P<0.05$ and absolute log2FC> 0.5) are Highlighted. Eight of the Probes Failed and were not Considered in the Analysis.

Supplemental Experimental Procedures

Morpholinos, RNA and DNA injections and chemical inhibitors

To assess the role of TGFβ signalling in definitive haematopoiesis, we designed MOs targeting either the 5'-end or a splice site of *tgfβR2*, *tgfβ1a*, *tgfβ1b* and *tgfβ2* (see below; Fig. S2,S3). For splice blocking MOs, correct targeting was validated by PCR on cDNA from morphant embryos (Fig. S2, S3) using the primers indicated (see below). We validated the phenotypes induced by translation blocking MOs by comparing them against those induced by splice MOs for *tgfβR2*, *tgfβ1a*, *tgfβ1b* (Fig. S2,S3, data not shown). For *tgfβ2*, both MOs yielded the same phenotype (data not shown) but only tgfb2^{MO3} could be validated by PCR (Fig. S3G) so all experiments were done with tgfb2^{MO3}. For tgfbR2^{MO1}, the phenotype was further verified by western blot against TgfβR2 (Fig. S2B).

To investigate whether the Notch or Vegf pathways regulated the transcription of TGFβ ligands, we treated wildtype embryos with DMSO (control), the γ-secretase inhibitor inhibitor DAPM (565777, Calbiochem) or the Vegf inhibitor DMH4 (D8696, SIGMA) from tailbud stage until collection at 22hpf or 28hpf for analysis by in situ hybridization, at concentrations specified in the figures. To interrogate *kdrl*, *tgfb1a* and *tgfb1b* gene expression in endothelial cells of DMH4-treated embryos, treated and untreated Tg(Fli1a:gfp) embryos were dissociated and GFP$^+$ cells isolated and processed for mRNA extraction with the RNEasy Micro kit (Qiagen) as described (Monteiro et al., 2011). cDNA was synthesized from total RNA using a Superscript III RT-PCR enzyme (Invitrogen) following the manufacturer's instructions. The primers used for quantitative real-time PCR (qPCR) are shown in below. Fold changes in gene expression were calculated using the $2^{-\Delta\Delta C_T}$ method (Livak and Schmittgen, 2001) and normalized to a geometric mean of *bactin2* and *ef1a*.

To rescue the loss of HSC markers in *tgfbR2* morphants we transiently expressed *jag1a* (Ensembl ID: ENSDARG00000030289) in endothelial cells under the control of the *Kdrl* promoter (Jin et al., 2005). The jag1a sequence was PCR-amplified from 24hpf embryo cDNA and a C-terminal V5 tag was added

in frame in the 3'end primer (see below for primers). This amplicon was then cloned into a Tol2 destination vector, downstream of the Kdrl promoter using the InFusion HD Cloning kit (Takara Clontech). The resulting Kdrl:jag1a-V5 construct was confirmed by sequencing. Additional details available upon request. The amount of DNA used for the rescue experiment is shown in the figure legends.

Western blotting

Protein extracts were prepare as described (Link et al., 2006); samples were sonicated in a Bioruptor sonicator (Diagenode) prior to loading on gel. After transfer, membranes were blocked in 5% milk (SIGMA) in Tris-Buffered Saline+0.5%Tween-20 (TBST) for 1h at RT. TgfbR2 protein was detected by a primary anti-tgfbR2 antibody (diluted 1:250 in blocking solution, sc-17792, Santa Cruz). A goat anti-mouse HRP-conjugated was used as a secondary antibody (1:1000 in blocking solution, P044701-2, DAKO) and developed with ImmunoCruz luminol reagent (SantaCruz). Blots were stripped with Invitrogen's stripping buffer, blocked again in 5% milk in TBST and re-probed with an anti β-actin-HRP conjugated antibody (1:35000, A3854, SIGMA).

NanoString expression analysis

This technology is as sensitive as qPCR but gene expression levels are obtained by counting mRNA molecules that hybridise with specifically tagged probes and does not require amplification (Geiss et al., 2008). We used 100ng of total RNA to hybridise with the capture and reporter probes overnight at 65°C. After the washes, the Target/Probe complexes were eluted and immobilized in the cartridge for data collection in the nCounter Digital Analyzer according to manufacturer's instructions. The raw data was analysed using the NanoStringNorm R package (Waggott et al., 2012). The data was normalised using the geometric mean of the six positive controls and then it was background corrected by subtracting the mean and 2 standard deviations of the eight negative controls. The data was then

normalised for sample/RNA content using the geometric mean of five housekeeping genes (*bactin1*, *bactin2*, *gapdhs*, *sdha* and *ubiC*). Normalised mRNA expression levels were log2 transformed and analysed using a t-test to identify differentially expressed genes between conditions. The pheatmap package (Kolde, 2013) was used to generate heatmaps. The values used were the scaled per gene normalised values from the NanoStringNorm package (Waggott et al., 2012).

Immunohistochemistry and apoptosis staining

To evaluate apoptosis in *tgfbR2* morphants, we stained for apoptotic cells using the Click-IT TUNEL® Alexa 594 kit (C10246, LifeTechnologies) followed by immunostaining against GFP. Briefly, embryos at the desired stage were fixed for 1h at RT in 4% paraformaldehyde in PBS (PFA), permeabilized in PBS+0.25% TritonX-100 for 20min at RT and then washed twice in deionized water. The TdT and Click-IT reactions were performed according to the manufacturer's instructions. For detection of GFP following the Click-IT reaction, samples were blocked in 3%BSA, 5% goat serum in PBS+0.5% TritonX-100 (PBST) for 1h at RT and incubated overnight at 4°C with a chicken anti-GFP antibody in blocking solution (1:500, ab13970, Abcam). Samples were washed 6x15min in PBST, blocked again in 3%BSA, 5% goat serum in PBST for 1h at RT and incubated overnight at 4°C with a goat anti-chicken Alexa 488 conjugated antibody (1:500, A-11039, Invitrogen). Following 6x15min washes in PBST, the embryos were mounted in Vectashield® and imaged in an LSM780 confocal microscope with a LD C-Apochromat 40x/1.1 W objective.

Morpholinos, primers and NanoString probes

Antisense Morpholino Oligonucleotides (MOs) Used in this Study.

Gene	Ensembl ID	MO name	MO type	MO sequence (5'->3')	amount injected	published	comments
**tgfbR2**	ENSDART00000039832	tgfbr2 MO1	ATG	ATATCGCTCCATTAGAAACGCAGTC	12.5ng	this study	
		tgfbr2 MO2	e4i4	ATATTAAGTTGTCTCCTGACCTGCA	10ng	this study	
**tgfb1a**	ENSDART00000060839	tgfb1a MO1	ATG	CAGCACCAAGCAAACCAACCTCATA	10ng	this study	did not work
		tgfb1a MO2	e2i2	TGGTGCAACAATCACCTCACCTGAA	15ng	this study	
		tgfb1a MO3	e4i4	GGACAGCAAAAAGACTTACTCATCA	2ng	this study	
**tgfb1b**	ENSDART00000028981	tgfb1b MO1	ATG	GTAATAAACTCTCCGCCTTCATGGT	1ng	this study	
		tgfb1b MO2	e1i1	AAGGATAGTGCCACTCACTCATTGT	20ng	this study	
**tgfb2**	ENSDARG00000027087	tgfb2 MO1	ATG	GGAGGCTCAAGACGTACAAGTTCAT	5ng	this study	
		tgfb2 MO3	e2i2	AAAGGGACTTTGGATTTACCTGGTA	7.5ng	this study	
**tgfb3**	ENSDART00000019766	tgfb3 MO2	splice	CATCATCCCTAAGGGAAACTTACTG	17ng	(Cheah et al., 2010)	
**kdrl**		Kdrl MO		CCGAATGATACTCCGTATGTCACTT	4.5ng (in combination with kdr MO)	or MO2 kdrl (Bahary et al., 2007)	
**kdr**		Kdr MO		GTTTTCTTGATCTCACCTGAACCCT	4.5ng (in combination with kdrl MO)	or MO1 Kdrb (Bahary et al., 2007)	
**bmp4**		bmp4 MO	splice	GGTGTTTGATTGTCTGACCTTCATG	2ng	(Chocron et al., 2007)	
**wnt16**		wnt16 MO		AGGTTAGTTCTGTCACCCACCTGTC	5ng	(Clements et al., 2011)	
**runx1**		runx1 MO	splice	AGCGCTCTTACCGTATTTGGCGTCC	5ng	(Gering and Patient, 2005)	
**jag1a**		jag1a MO	splice	AAGCCAAACCCGCACATACCCGCAT	6ng	(Yamamoto et al., 2010)	

Primers Used in this Study.

Gene	Acession number /Ensembl ID	primer name	primer sequence	purpose	anti-sense linearisation	in vitro trans-cription
**tgfb1a**	Acession:NM_182873	tgfb1a F1	TCAGACGCTTTTCGATCCTT	generate in situ probe; test splice MO	ApaI	Sp6
		tgfb1a R1	AGGACCCCATGCAGTAGTTG			
**tgfb1b**	Ensembl ID:ENSDARG00000034895 (ENSDART00000028981)	tgfb1b F1	GCACACCATAGAAGATCCAACA	generate in situ probe; test splice MO	ApaI	Sp6
		tgfb1b R1	TGACAACTGTTCCACCTTATGC			
**tgfb2**	Accession:NM_194385	tgfb2 F1	GTTCAAGAAGAAGCGGATCG	generate in situ probe	SpeI	T7
		tgfb2 R1	GGGGTCTTGCCGATGTAGTA			
	Ensembl ID:ENSDART00000030271	tgfb2 F2	GTTCAAGAAGAAGCGGATCG	verify splice MO		
		tgfb2 R2	TGTCTGCGCTCCACAGATAC			
**tgfb3**	Accession:NM_194386	tgfb3 F1	TGGCTGACAAACAGAGCAAC	generate in situ probe	ApaI	Sp6
		tgfb3 R1	CTGCCGTGTGACAGAGGTAA			
	Cheah et al, 2010	tgfb3 F2	TCACACTTAGTTCATGTTAG	to verify splice MO (362bp in MO, but not in WT)		
		tgfb3 R2	TGTAGCGCTGCTTGCCGA			
	Cheah et al, 2010	tgfb3 F4	CGGGCAGGACAACACTGA	qPCR		
		tgfb3 R4	GGCAGTAGGGCAGGTCATTG			
**tgfbR2**	Accession:NM_182855	tgfbr2 F1	CACACATGCCAACAACATCA	generate in situ probe	ApaI	Sp6
		tgfbr2 R1	TCTCATTTGTCGTCGCTCAC			
	Ensembl ID:ENSDARG00000034541	tgfbR2 F2	TCAGTCCGGATCACACGATA	verify splice MO		
		tgfbR2 R2	CGACAGCGAGTTGTCCAAAC	verify splice MO		
**gata2b**		gata2b F1	ATGATGGATGCCCCAGCG	generate in situ probe	SacI	T7
		Gata2b R1	TCAGCCTATAGCAGTGACTAAGC			
**jag1a**	Yamamoto et al, 2010	jag1a F5	GACAGACAAACCGGGATGAT	verify splice MO		
		jag1a R5	CACCGCTTTCTCGATCACTT			
**rspo1**	Ensembl ID:ENSDARG00000039957	rspo1 F5	AGAAGCTCTACTCCATGGCTTG	qPCR		
		rspo1 R5	GACAGAGGCCTGGTTTATTTTG			
**baxa**	(Danilova et al., 2011)	Bax F1	CGTCGGGTGGAGGCGATACG	qPCR		
		Bax R1	GAGTCGGCTGAAGATTAGAGTT			
**baxa***	Accession:NM_131562	Bax F2	GGAGATGAGCTGGATGGAAA	qPCR		
		Bax R2	GAAAAGCGCCACAACTCTTC			
**p53**	Accession:NM_001271820	p53 F1	TTAAGTGATGTGGTGCCTGCCT	qPCR		
		p53 R1	AGCTTCTTTCCCTGTTTGGGCT			
**ef1a**	(Bertrand et al., 2008)	ef1a-F1	GAGAAGTTCGAGAAGGAAGC	qPCR		
		ef1a-R1	CGTAGTATTTGCTGGTCTCG			
**bactin2**	Ensembl ID:ENSDARG00000037870	bactin2 F1	GGACCTGTATGCCAACACTGTA	qPCR		
		bactin2 R1	ATGTGATCTCCTTCTGCATCCT			
**gapdh**	(Simoes et al., 2011)	gapdh F1	GGTCATTGATGGTCATGCAATC	qPCR		
		gapdh R1	CACCTGCATCACCCCACTTA			
**taz**	Ensembl ID:ENSDARG00000041421	taz F1	GGAGAATATCCAGCCGAGTG	qPCR		

	Ensembl ID:ENSDART00000138805	taz R1	TGCACCATCAGCGAGTTAAA	
**cdkn1a**	Ensembl ID:ENSDART00000136722	cdkn1a F1	AAGTGGAGAAAACCCCAGAGA	qPCR
		cdkn1a R1	TAGACGCTTCTTGGCTTGGTA	
**cdkn1a***	Ensembl ID:ENSDART00000136722	cdkn1a F2	AACGCTGCTACGAGACGAAT	qPCR
		cdkn1a R2	CGCAAACAGACCAACATCA	
**cdkn1b**	Ensembl ID:ENSDART00000076417	cdkn1b F1	ACGGGAATCACGACTGTAGG	qPCR
		cdkn1b R1	CACGATGAGTCGAGACAGGA	
**jag1a**	Ensembl ID:ENSDART00000137172	jag1a F3	ATTGGTGGATACTTCTGCGAGT	qPCR
		jag1a R3	CCATTCACCAGATCCTTACACA	
		jag1a kozak F	GCCACCATGATTCTCAGACCGAGCGC	amplify jag1a+C-terminal V5 tag
		jag1a V5 R	CTACGTAGAATCGAGACCGAGGAGAGGGTTAGGGATAGGCTTACCTACGATATACTCCATTTTCTGCAAG	
**gata2**	Accession:NM_131233	gata2 F3	GGACGAAAAGGAGTCCATCA	qPCR
		gata2 R3	GCACTCATAGCCAAGCTTCC	
**dll4**	Ensembl ID:ENSDARG00000070425	dll4 F4	ACGCATACAACCCTAACATGC	qPCR
		dll4 R4	CTCTGTCTGCTTCCCACTTTG	
**tnfr2**	(Espin-Palazon et al., 2014)	tnfr2 F	CACACAAGAGATCCGAAGCA	qPCR
		tnfr2 R	GGCATCTGTGATGGGAACTT	

*primers used for qPCR experiment shown in Figure S5K.

Sequence of the NanoString Probes Used in this Study.

Pathway	gene name	accession number	target sequence
**VegfA signalling**	vegfaa	NM_131408.3	TATTTCTCGCGGCTCTCCTCCATCTGTCTGCTGTAAAGGCTGCCCACATACCCAAAGAAGGGGGAAAGAGCAAAAATGATGTGATTCCCTTCATGGATGT
	vegfab	NM_001044855.2	ATGGACTAAAAGTACGCATGGATATCGCCTTCTCTCTTCAGCGCCTTTGTGTTCACTGATATCTCTGTATACTCCGCTAATAACACAGTGTCGGCAATAC
	VegfC	NM_205734.1	TCAGCAAGACGTTGTTTGAAATCACAGTTCCAGTCAAGCAAGGGACCAAACCGGTCACCATAAGCTTCGCCAACCACACTTCCTGCAGCTGTTTGTCAAA
	kdrl (flk1)	NM_131472.1	AACATACCCAAACCAAAACGTTATCCTTGAGACGCAGATGAATCCTATGGCAGATGATGTTAAAAGAGGGGTACAGTGGGATCCAAAAAAAGGTTTCACG
	kdr	NM_001024653.2	GCCCTATTGAAGGACAGGATGTTATAATGCGTTGTGTAGCAGACCGGCTGCTGTACTATAACTTGCGCTGGTATCGGGTAGCAAATGTAGCCAACCATGA
	mflt1	NM_001014829.2	GAAGCACTGGTTTCTGGCATCTATCGCTGTGTCACATCCAACATATTAGGCAGAGATGAACTAGACATTCCTTTCTATGTCACAGATGTCAAAGAAGGCC
	sflt1	NM_001257153.1	CAGCTTCCCAGCAGCGTGATTGTCCCTCCGTCTTCAAACGTGGCACAGTCCGCTTCCTCAGGAGCTTGATCTATTCTTGAGAGTCCCTGATCCCACACAC
	flt4	NM_130945.1	TCCTGACCTAAAAGTCACTCTCTTCTCGTTAGTGCCGTATCCAGAGCCTGTGGATGGCAGTGTGGTCACCTGGAATAATAAAAAGGGTTGGTCGATTCCC
	plcg1	NM_194407.1	TTTTACGCAGTGGATCGTAACCGAGAGGACAGAATTTCCTGTAAGGATCTGAAATGTATGTTGTGTCAGGTCAACTACAGAGTGCCCAACATGAAGTTCC
	tel1	NM_001044968.1	CCTGGTGGAACTAGTGGACTATTTCCGAAAGAAGCCGCTGTATCGAAAAACAAAACTGCGCTATCCTGTTACACCAGAACTAGTAGAGCGCTTCAGTTCG
	cbfa2t3	ENSDART00000021009.2	GCAACACTGCACGTTACTGTGGCTCTTTCTGTCAGCACAAGGACTGGGAGAAACACCATCATGTATGCGGCCAGGGTCTGCCTAGCAGTAGCGAGAGCAC
**Notch signalling**	notch1a	NM_131441.1	CAACTCTGATGATTGTGCATCTCAGCCGTGTCTCAACGGAAAATGCATCGACAAAATCAACTCGTTCCACTGCGAATGCCCTAAAGGGTTTTCTGGGAGT
	notch1b	NM_131302.2	GCCCGAGCAATTCAAGTAGAACAATGAGATTCGCTAAATGAAGGCACATTCCATAAGCCTGCTTATCTGAAGGGTCTTTATATGCCGCTTGTACTAACCT
	notch2	NM_001115094.1	CCATTCGGGATTCCTGTTTACAGCCATCTGCGATCTGAGGCAATGTTACCATTGAACGATCACACTGCCAAAAGTAAGGAGTCTCATTTTGGGGGTACTG
	notch3	NM_131549.2	TCAAACAGCTTGGACTGCATACAGTTGCCCAATGATTACCAATGTGTGTGCAAACCTGGCTTTACAGGACGAGGGTGTCAAAGTAGATTCAGCGTGTGCG
	dll4	NM_001079835.1	TCACCTTACTCGGATCTACCTGTAGACGCATACAACCCTAACATGCAGATAAGCTTTTTTGCCATCCAAAGAAAGTTGGAAGTAGGGGCTGAATGGTCTC
	dlc	NM_130944.1	GTCAACATTCACTGGTGGTAACTATTTCCGACATTCCCAAGGCTTGAAAGGACCCTTGGTCCAAACTGATAGCCTCAACATTGTCAAGATTTCCTGGGTT
	dld	NM_130955.2	AAGGACTTGTCCGTAAGCATCATCGGAGCCACGCAAGTGAAAAACATCAACAAGAAAGTGGACTTTCAGAGCGACGGCGACAAAAACGGATTCAAATCGC
	jag1a	NM_131861.1	GATGATTCTCAGACCGAGCGCAACTTTTGCGGCGCTGTCCGCTCACGTGCTGCTGAGGTGCTTGTGGATGCGGGTATGTGAGGCTTCGGGGCATTTCGAG
	jag1b	NM_131863.1	GGCTTGCAGTTTCGGAACTGGATCTACGCCCGTTCTCGGTGGGAATAAATTTTCGACCAAGGGAACGCGCAGTGAAAAGTCAAGGATTGTTTTACCCTTC
	jagged2	NM_131862.1	TGAAGACCCACAATCCCTTCCCTTACCAGTCCTCCTGTTGCTGATCATAAAACCGAGACCTGCCGGTTCATGCTTGTTGCTTGTAAAGATGCTGATATGT
	hey1	NM_212561.1	AACATCAGACTATTTTGAACAGATGCACAGAAACAGACTTGTACAAAGGGAAGACACGAATACAGATCCACTCTACTAACCAGAACGACTGTGCTTGTCC
	hey2 (grl)	NM_131622.2	GGAATGAAGTTTGAGACCTCCATTCGACGGCTCGGGGCGTGTTTTCTATTTTTTTTTTACGGTGGGTGTTCCCGAAGCAGGACGTGGGCGTGAATGTGAG
	efnb2a	NM_131023.1	ACTTTGGAGTTTTAGTGATCGCGTGCAAGGTGAACCTGTCCCGCGCGCTCATCCTGGACTCCATATACTGGAACACCACGAACACCAAGTTTGTGCCGGG
	ephb4a	NM_131414.1	ACCTTGTAAACATTCACAAGTAAAACACTCGTGATTCCGCGATGGAGCTCTTCTCCAGGAATGTGGCCGCGTTTTGGGTTATCCTGCTGGAGTTCCTGCT
**TGFβ signalling**	tgfb1a	NM_182873.1	CTGGGAACTCGCTTTGTCTCCAAGGACTTGTCCAACCGCTGGCTCTCATTTGACGTGAAACAGACAATGATAGAATGGCTGCAGGGTTCAGAAGATGAAG
	tgfb1b	ENSDART00000134907.2	TGCAAGTGTTGCTGAAAGCATCATTTCCGTTTGGTCTTGATGAGCAAAGTTAAGAGAACAACACACTGAGGTGCTTGAGACCCAGCACTTTCTGAGGGGA
	tgfb2	NM_194385.1	AAGTGCACAAGATAGACATGCAGCCCTTTTACCCTTCAGAGAATGTCATCTTATCACAACATTACTACCCATACTTCAGGAGGCTGATGTTTGACGTGAG
	tgfb3	NM_194386.2	CGCCTAAAGTGGAGCAGCTTTCCAACATGATCGTCAAATCCTGCAAGTGCAGCTGAGAGGTCCGTTTTCAACCCATCCACGAGCCAAAAGCTTCTGCGTT
	eng	ENSDART00000125008.2	GTGCGTTGGGTTTTGGAAAATGAGGGACTGTCAAGCAACATCAATGTGCTGGTGCAGTTGTCTGTGAACTCCACGGCAGAGTCTTCGAGTCTGTCTGTCC
	tgfbr3	ENSDART00000109313.2	AGCTCACAGAACGAATTTTACCAGTAAACGGACATCATGCGACTATTTTGTTCAACTGCACCTACCGCAAGAACCAGGACAGTCCATTTGACACCGATGC
	acvrl1	NM_153643.1	ACGAAAACCAGACATGAAGGCTTCAGTGATGCTCGCAGTGCTGTTTGTTTTGGTGTATTCTGGAAGCTATGCAACAACTGCAGATCATCTTATGGAGACT
	tgfbR2	NM_182855.2	CTTCAAGCAAACCGATGTCTACTCTATGGCTCTAGTGCTGTGGGAAATCACGTCCAGATGTAACGCCATCGGAGAGGTTAAAGACTATGAACCTCCATTT
	tgfbr1a	NM_001037683.2	CAAGGACTGCTGTGTTACTGTGAACGATGTGTCAATCGGTCGTGCAACACAACTGGACTGTGTTTTGCCGTCATCGCTAAGTCTTCTGGACAGACCGTGA
	tgfbr1b	NM_001115059.1	CATGAAGCATTTCGAGTCCTTCAAGAGGGCTGATATCTACGCCATGGGCCTGGTGTTCTGGGAGATCGCCAGCCGCTGCTCTATCGGAGGTATTCATGAA
	smad1	NM_131356.1	CCCCGTGCTGGATTGAGATTCATCTTCATGGACCCCTGCAGTGGTTGGATAAAGTCCTCACCCAGATGGGATCTCCTCACAACCCCATTTCTTCAGTGTC
	smad2	NM_131366.2	CTGCAGCCAGTGACTTACTCAGAGCCTGCGTTTTGGTGCTCCATAGCTTACTATGAACTTAACCAGCGGGTCGGAGAAACATTCCACGCCTCTCAGCCTT
	smad3a	NM_131571.2	CCCACAACAATTTAGATCTACAGCCAGTGACATACTGTGAACCAGCATTTTGGTGCTCTATTTCCTACTACGAATTGAACCAGCGAGTAGGAGAAACTTT
	smad3b	NM_175083.2	ACAGCCTCTGGACTCCTCAGAGTGTTACTTTATCAATTACAGATCCCGATATTTGCTGTGGGCAAAAACTGCTGCATACAGTTGAAGCACTTTGAACGGT
	smad4	ENSDART00000035478.2	AGTTTGACACCCCTGCTTTAAAGTAAACATTTAACCGTGTCTCTTTGTGCAGTTTCTCATAACCAAAGCTATTTTCTCCTCAGGGCCTGTTCACAATGAA
	smad5	NM_131368.2	CGGTGTCTGTAAAACAGAGCAAAGGTCGACAGAGTCTACAGCATTTCTTGAAAGGGATGGTGGCTTTTCCGCAGGGAGGTGTTGAGAAGTGATTGAAAAA
	claudin5a	NM_213274.1	TCTACATCCTCAGCGCCATCTTCGTGCTTGTGCCACTGTGCTGGATGGCCAATAATATCATCTCCGACTTCTATAACCCGCAGGTGCTGCCCGCCCAGAA
	claudin5b	NM_001006044.1	TTTCTGCATGTCTGGAGATTGTTGGACTCTGCTTGACCGTCACCGGGACTCTTTTGGTGATGGTAGCCTGTGGGTTGCCCATGTGGAAAGTGTCCGCCTT
	id1	NM_131245.1	GACAACTCAACAAGCCTTTAAAAGGAGTACGAGCACCTCGCTTCAGCTATTCCTCTTTGACTTACTCGCAAATAGCCAAAATGAAAGTTGTGGGACCTAC
	Id2a	NM_201291.1	ATATGTGCAGCATAGTGACCTTGCCTTTTATATCCAGGACTCTGAAGCATGTCACTGACCCTTCAAACTTGCACATTCGCCCAAGGAATGTTCTATTGAA
	Id2b	NM_199541.1	ACAGATGACCTCATAGCCCTGTCTCGTTGAAATGGTGAGTTGATTCATCTTCATGCTTTGACAAGCCAAGTTAAACTTTTGCACGTGGGGCAGTTTGCTG
	id3	NM_152967.1	AAACGAGACAGACACACAAAACACGCCTGACATTTTCCTGTCAATGAAGAACTCAGAGATGAGCCGTAATTTCTCCAAAGAAGATGGAGCTATGTGCCAT
	zeb2a	NM_001135104.1	ATATGATTCTTAAACGTACAAAGTCTTAAAGTTGGCCAGGGGTGGACGGAAAGAGTCTGGGTAACTATTGACGGAGGCTCATTGTATGAGGCAATGTCAG
	zeb2b	NM_001245966.1	ACTGGATCGGAGACGGAAGAAGAAGACAGACTTTTGGTATCGGAGGAGGACGCTTTGCTTAATGGGGCGGGGAGTCCGGCCAGCCTTGTCAATCATGAGT
	twist1a	NM_130984.2	GATGCACGCGTTTGATGCAGCATGATTCTCGGCCTGAGGAGCTGAACTCACTGGAAGGAGCGGCTCAAAACAAGGGCGAAAATAAGGATTATAAAGGGAA
	twist1b	NM_001017820.1	AATCTTGGGGAAAACGGCAAATGTTCCAACAGAGGTCATGGCTGTTACCGAGAGAAGGCCACGGGCAGCGAATTGTCATATGGATTTCCTCGCGAGTCTT
	twist2	NM_001005956.1	ACGCGTCCTGATAATGCCGAACGGACTGTTTACTTCCACTAATTTTGAGGATGCCAAAGGATTATTCGATGAACCTCTAAACCTCAGTGACGTGGCCAAA
	snai1a	NM_131066.1	CCCTGACCGCCACATCTCTGAAAACAGGTGGCGTCTGCTGATTTCTCTAGAAACACACTTGGAGAGAGTCCTTATGGAGCACAGTTGACTGCTTTACTTT
	snai1b	NM_130989.3	AGACCCACTCCGAGGTGAAGAAGTACCAGTGCGGGTCGTGCTCTCGGACCTTCAGCCGCATGTCTCTCCTGCACAAGCACACACTGTCCGGCTGCTGTCC
	cdh5	NM_001003983.1	GGATTATGATTTTATACATGAGTGGGGACCTCGGTTCAGGACCCTGGCTCAGCTTTATGGAGTAGACGGCTCTGATTCGGATAGCTCCTACTGAAGTCCA
**Cell cycle and apoptosis**	cdkn1a	ENSDART00000113620.2	CACAGATAAACTCTTGACCTGCATCTACCTTCATCTGTCTGGAGCTGCATTCGTCTCGTAGCAGCGTTATTGACAGAAAACGACCTTCAGGAACCAGAGA
	cdkn1b	NM_212792.2	GGGGGAAGGGCTCCGATATTCACATCTCCTCCTCTGGGAATAATAACGACAATAACGTGGATGTTAACGGGAATCACGACTGTAGGGTAACGGAGCAAAG
	cdkn1c	NM_001002040.1	GAGCGAGACCAGAGCCGGTGGAATTTTAACTTTGAGACCAACTCGCCTTTGCCTGGAGATTACGAGTGGGAGGCGATTTCAGAGGACACTTTGCCGTTTT
	cyclinD1	NM_131025.3	ACACGGTGCAGAAATTCATGTATAAGTGGCTGCCTGTCTTGTTCTGAACACAGGAGACCTGGCAGGTCCCCACCAAGTCGTTGTCTTAATAAATGTGACG
	tp53	NM_131327.1	TGAGGGGCAGGGAGCGTTATGAAATTTTAAAGAAATTGAACGACAGTCTGGAGTTAAGTGATGTGGTGCCTGCCTCAGATGCTGAAAAGTATCGTCAGAA
	perp	NM_001256207.1	ATGGAAGTCAGTGGGTGCATTTGTAGGTGAGATTTTTGGCGTGAACTGTCCCTCTAACAGGTATACATGCATCTCTCCCATAGTTAATTCTGATAGGTGG
	baxa	NM_131562.2	GTCTCCACTGCAAGGGCTTCCAAATGTCTGTGAGTTGTATTTACAGGTACAAGATGAGCCTCTAAAGTTTATGAGGTGGCGTGGCAGCAGTTGAGCGCAG
	bcl2	NM_001030253.2	TGGAGGTTGGGATGCCTTCGTGGAGATGTACGGTCAGCAGAGAGACTCTGTGTTCCACCCGTTTTCATACCTAACAAAAGTGCTCGGCTTGGCGGCGCTG
	bcl2l1	NM_131807.1	GCTTTGCAGAGATCTTTGGAAAAGATGCAGCGGCGGAAAGCAGGAAATCGCAAGAAAGCTTCAAGAAATGGTTGTTTGCAGGAATGACCTTGCTCACGGG
	pcna	NM_131404.1	TGTGACCCTCTAAAGCCATTACATATCATTGGCAGAATTGCGGGCGCACTTATTAAATGATGGTAGTTTGGGCCTTAGCTTTACCAGGCGCTCCGTGGGC
	bmp4	NM_131342.2	GATTCGTTTTTAATCTCAGCAGCATCCCAGAGGACGAACTCATATCCACCGCAGAGCTTCGCGTCTACAGGCAACAAATAGATGACGCCTTCTCAGACCC

**BMP, Shh and angiopoietin signalling**	bmp2b	NM_131360.1	AAGTTTTCATCACGAAGAGGCTTTCGAGGCACTGTCCAGCCTGAAAGGAAAAACAACGCAGCAGTTTTTCTTCAACCTTACCTCCATTCCTGGCGAGGAG
	bmpr2a	NM_001039817.1	CAGTTCTGCTCTCCTGAAACGTCAGCCTCCCACGGCCCGTTTTATCCTCTCATGAAGATGGTTTCTGAGGTTTCGGGGTCACAAGGATCCAGTAGGCATG
	bmpr2b	NM_001039807.1	CACATGAGCTTGAACTTCGTTCGATAGAAAGAAGAAAGGGGGAACAGAAAATCTAGTTACTGGAACTTTTTTTGGAGCTGAATCCATCCAGAAGCGGCAG
	shha	NM_131063.1	CCACGACGCGACGTGTGTTTTACGTCATAGAAACGCAAGAACCCGTTGAAAAGATCACCCTCACCGCCGCTCACCTCCTTTTTGTCCTCGACAACTCAAC
	shhb	NM_131199.2	GAAACATACAAGAGATTATATGAGATATGAGAAAGCGCAGGGCTGTGCAGTGTGCAGCGCTCTATTTTATCGGATAACCATAAAGGATGGCGCTCAAAAG
	ptch2	NM_130988.1	GCCACGCCGCTTTTGCTTTAAAACAGATTTCTAAGGGGAAAGCTGTGGGACAGAAAGCACCACTGTGGATTCGGGCGAGGTTCCAGGCTTTTCTCTTTTC
	gli1	NM_178296.2	GAACATGATGACATCCCATCATAATCTCCCTCATAACCAGCACACATCTGAACTCATGGCATCAGGAGACGCCTCTTGTTTCTCCACTCCTCGCTCGATG
	gli2a	NM_130967.1	GTATTTTGTCTCATCTAAGCTGTGTGATACTGAAGGACAGCATCTCTGCGCTTCAGCCTGTGTAGGCTTGACGAACCTTGCGTCCCTTTGAGATGTCTTT
	angpt1	NM_131813.1	GCTGACGAGGAGCGAAAGTTTCGGGATTGTGCTGATCTTTATCAAGCAGGCTTCCAGAAAAACGGAGTTTACACCATCAATATTAGCCCACAAGAGACCA
	angpt2	NM_131814.1	TGCAGAGGAACACCAGGCTTATTCTCAGTATGACACTTTCTACATTGACGGAGAAGATAAGAAGTATAGTCTCCATGCCAGGGGTTTCAGTGGTACTGCT
	tie1	ENSDART00000003701.2	GGGATAAAAATGAGTGCTGCAATTAAAATGCTCAAGGAGTTCGCCTCAGAGAATGACCATCGAGACTTTGCTGGAGAATTGGAAGTGCTGTGCAAATTGG
	tie2	NM_131461.1	GCGGCTCGAAACGTCCTGGTGGGAGAGAACTTTGTGGCGAAGATCGCAGACTTCGGGCTGTCCAGAGGTCAGGAGGTGTACGTCAAGAAGACCATGGGTC
**Haematopoietic/vascular genes**	fli1a	NM_131348.2	ACTTCCTGAGACTCACCAGCGTTTATAACACCGAGGTCCTTCTCTCACATCTCAATTACCTCAGGGAAAGTAGCTCATCGATATCATACAACACGCCATC
	fli1b	NM_001008780.1	GTAATTTCTTCACGCCTCAATCCACCTACTGGAACTCCGCAACCAGTGTGGTTTATCCCAGTTCACCGATGCCACGACATCCCAGCACTCACACTCACTT
	aplnra	NM_001075105.1	TCAGCTTCCAGTGAGATAAACTTTACCACAGACTCTTGTATCAGACAGAAGAATCCAAACATTTTCAGTGAACTCTATAAAAACTGCTGCCAGAGATTGG
	nr2f2	NM_131183.1	GTATTACAGACGCTTACCTAAGGTTGCGCCCAAGCGAATTTTCGCTCAAGTGTTTAGCCTCAGTAAACTGTATTTGTGAACGTGGGGAAATGATGGGTCA
	dab2	NM_205757.2	GGCCAGATTTCAGGGTGATGGAGTGAGATATAAAGCCAAACTTATTGGTGTGGATGATGTTCAAGATGCAAGAGGGGATAAAATGTGTCAGGATTCCATG
	scl alpha	NM_213237.1	TAGCAATCGAGTCAAGCGCAGACCTGCACCTTATGAGGTTGAAATCAACGATGGTTCGCAGCCCAAAATTGTGCGACGGATTTTCACGAACAGTCGCGAG
	lmo2	NM_131111.1	GCGTACACAATGTGTGCTGGATGTTTCTGACCTTTGATACACTTGCTAAGACAGCAGAACAGGTGCATCTCTGAAGCGTTTTGTGCGGCAGATGGTCTTT
	lmo4a	NM_177984.1	ACATCTTCCCTCCAATCTCTCCTCGTGAATGTCCACCTTCACCCGAGACCTTCTGCCCACTTCTGCGCATCCAGCCACCTTAAAAGAGTTCAGAACTACG
	lmo4b	NM_212689.1	TCTCAAGTGTTTCACATGTTCTACCTGCCGGAACCGGCTTGTCCCTGGTGACAGGTTTCACTACATCAACGGCAGCTTGTTTTGTGAACACGACAGACCC
	ldb2a	NM_131314.1	TGTCCAACCCTCCGCACGACCCCTTCTACTCGTCCCCTTTCGGACCCTTTTACCGGAGACATTCACCGTACATGGTGCAGCCCGAGTACAGAATCTACGA
	tbx20	NM_131506.1	GTTTCACGAACTCGGCACTGAAATGATTATCACAAAGTCTGGAAGACGAATGTTTCCGACAATTCGTGTGTCATTTTCCGGAGTGGACCCAGACGCCAAA
	hhex	NM_130934.1	GAGCATCAGTTCACCCTATGATAAGCCCTGCTCTTACTGTAAATACTGCACCATACTGTACATTCCGCCATACCTGACCACTGACTTAATGGATGTGTAT
	gata2a	NM_131233.1	ATTTACTGAGTCACTTTGGTACTGAAAGAGCGGACGCAGAATCACTGTGTGGTAGTCAAAACGGCCACCTCAAAACTCTCATAAAGGACTCGCTTTGAGC
	gata2b	NM_001002689.1	CCCTATACCTTCATATCCCGATTACTCAGTAGCCGGAGCGCACGAGTATCCCGCCAGTGTGTTTCACTCCAGAAATCTGCTCGGAAACATGACGACAAAA
	etv2	NM_001037375.1	CTTTGGCAGTTTCTGCTAGAACTCCTGCTGGATTCTGCTTGCCACACTTTTATAAGTTGGACTGGTGATGGCTGGGAGTTTAAAATGTCAGATCCCGCTG
	gata1	NM_131234.1	ACAGACTCTGGTTTACTGCCACCCGTTGATGTAGATGAACCTTTCTACTCAAGCTCTGAGACTGACCTACTGCCATCGTATTATTCCACCAGCGTCCAGA
	tif1g	NM_001002871.1	ACTCGAATCGTGAACGTCATGCGTTGTACAGTGTGCCATCAGGACTACAAACAGATTGACATGGTGGACAATTACTTCGTCAAGGACACTTCAGAGGCCA
	alas2	NM_131682.2	GCGAGTGGGAGAAGTCTTATTTTGGTAACATGGAGCCAAGATACATCACTGTGGCTGCACAATGAGTCCATTTTAACAGCTCAAGATGTAGATCTTCCAT
	gfi1b	XM_001922262.1	AAAGAGCTTCGGCCATGCAGTCAGTCTAGAGCAACACATGAACGTCCACTCACAGGAAAGAAGCTTTGAGTGCAAAATGTGCGGTAAAACCTTCAAGCGC
	spi1	NM_198062.1	TCTTGGGATTATAACACGCATCACATTCATCCAGTGGACTTCGAGAATCTGCCGGAGAGCCATTTTACAGAGCTACAAAGCGTGCAGTCACTACATGCGG
	mpx	NM_212779.1	GAGAGAGGCTGTTTGCCTTCACATCCCACATAGCTTTGGATCTGGCTTCCCTCAACATGCAAAGAGGGCGTGACCATGCTATACCAGGTTATAATGCATG
	mpeg1	NM_212737.1	TAAAACCAGTTCGTCTTCCACCGTTCACAAGACCACCAGTGGTCGGCATGATTGCGACAAACACAGTAGCCGTAATGACAGAAGGTGAGCGTTCTTGGGT
	csf1ra	NM_131672.1	TGTACTGAAGGAGTGAAAGATCAGTCTATATTTCCTCTGAGACGCTGAAGACTGCTGAATGGTTAAGCAGGAGCAGCTCTGAGATGTTCTTCGCGCTCTT
	cebp1	NM_131837.1	AGCGCTCAGACTCCGATGGACTCGGCCCTGTATACTCAGACCATCAGCTTTACTAAGAGTCCCGAGGTGATGATGGGTTATTTACCGTACTCCTCCTGTC
	lcp1	NM_131320.1	ATGAGGAGGATCTAGAAGCGGATTCCATTCCACTGGTCGTCTCCTATACTCCATTATACGCCTGAAACAGCTCAGAGTACGCCACGCTGAACTGTGGACA
	cmyb	NM_131266.1	TGAAGACTGGAAAGTCATCGCCAGCTTTCTACCGAATCGAACAGATGTTCAATGCCAGCATCGTTGGCAGAAAGTCCTCAACCCTGAACTCATTAAAGGA
	ikzf1	NM_130986.1	GAGAGAATCGTCTGTCAGAGCTATCTTTCGAGAGTGGCTCAGGTGAGCTGATGCAGCCCCATGTGATTGATCAGGCCATCAACAGTGCAATTAGCTATCT
	runx1	NM_131603.2	AAAAGCAGACACTTATGCTCTAAAATGTCGTCACTAATGACTAAATCACTGATGAATATCCACAAGATAAAGGTAAACATCGCTGAAGTGTAGCTCTGAC
	cbfbeta	NM_199209.1	TCCCGGATCAGAGGAGCAAGTTCGAGAACGAGGAGTTCTTCAGGAAACTCAGCCGCGAGTGCGAGATTAAATACACCGGTTTCCGCGATCGGCCGCACGA
	itga2b	NM_001003857.1	TGCTGTCGCTTCCCTGGAGATGCGTGGAGTCTCTTCTCCTGTTGACTGTGTCCTGCCTATTTCTAAATGGGAGTCGAAGGATTATCCGGAGGACTTGGAT
	dnmt4	NM_001025450.1	TGTAGCACTCTGATGCCTTAATGTGTGGTGCCTTTTGAAGAGAACAGATACAGCCTGCCATACGGGGGTTGCGAAGAAACTTTCTGCCTTTGTTTGCTGT
	gfi1.1	NM_001020776.1	ACAGGCTAGATTGGAGAGCTGTGAGTGATTGGTGACCCTGAAGCTGATAACGGAGAGTAAACATGCCGAGGTCATTTTTGGTGAAGAGCAAACGGGCGCA
	gfi1	NM_201338.1	AGCGGCCTACTCCAACCTATTACAATGAGCGCGTGACCGAGCCCTCTATTTTCACCGAACGAGGATCTGGCGCAAGAATTTACAACAGCTATGGCTCCAC
	hbbe1.1	NM_131759.1	GCTGATTGCTTGACCATCGTTGTTGCTGCTCAGATGGGTGCTGGATTCACACCTGAAGTTCAGGCCGCTTTCCAGAAATTCATCGCTGTTGCGGTGTCCG
	urod	NM_131347.1	TGGGTCACGGCCTTTACCCTGATATGGACCCAGAAAATGTGGGCGCATTCGTGGAGGCTGTACATAACCACTCTCGCCAGCTTCTCAAACGCTAATAAAA
	ptprc	ENSDART00000105607.2	ATGCTTCATTCTTAGATGGCTACTGGTGTCAGAAGAGTCTGATCGCAGCACAAGGGCCTTTACCAAACACAACAGCAGAGTTTCTGCTCATGCTGTACCA
	klf2	NM_131856.2	CTGGACCTAAACCTCAAGACGGAACTGGAATCAGACAGTATAATGTGCTTTAAGTTAACATGATGTGCTGCGGACATCACGACAGTGCCTTTTAATATGC
	klf4	NM_131723.1	TTTTAATATGTTTAGCGAACCACTGCGGGCAAATCACCCGGCCATGCCAGGTGTGATGCTGACTCCACCATCCTCACCTCTCCTGGGATTTTTAAGCCCA
**Wnt signalling**	rspo1	NM_001002352.1	TTCATCTTACTGGAGCGAAATGATATCCGTCAGATAGGCATTTGCCTGGCCGCGTGTCCTGTTGGATATTATGGCATTCGAAATCGGGATATGAACAAAT
	wnt2bb	NM_001044344.1	CCCTTGGTGCACGTGTGATCTGTGACAATATTCCTGGGCTGGTGAATAAACAGAGACAGTTGTGTCAGAAATATCCTGACATCATGCAATCGATAGGTGG
	wnt16	NM_001100046.1	CCAAAACAATGTTGACAGACTGCCGCTGTCATGGCGTATCGGGTTCATGTGCGGTAAAGACGTGTTGGCGCACAATGGCTGCATTTGAGCGTGTGGGCGC
	fzd2	NM_131140.1	GAAAGACACTGCACTCTTGGCAGAAGTTCTATGTGCGTTTGACCAGTGCTGGACAAGGAGAAACCACTGTTTGAAACGAGACATTGTGATCCACAATTTC
	fzd7b	NM_170763.1	ACAACGGTGTGAACTGAACTTTGTGGACTGCATGTGAGAAATACAGGTCAATATAGAAAGGAAACGTGAACTGCGATCAGGTCACACATTTGCTGATGGG
	taz	NM_001001814.1	TCTGGGGAGTCCTTAAGTTCCGGCAGTTGTGGAACCTTAACAAGATGAGATGGACACCTGCTGCCTCTGATATCTGTTTCACAAGAGAATTTCACTCCAG
**Housekeeping genes**	actb1	NM_131031.1	GATCTTGCAGGACTTCCCTAGGGTATGTGAATAAGGGATGTCCCTTGAAAATGTAAGCCAGGGTGTCTCTGTACACTGACAAGTCAACCCAAATAAAACG
	actb2	NM_181601.3	CCTGGGCATATTGTAAAAGCTGTGTGGAACGTGGCGGTGCCAGACATTTGGTGGGGCCAACCTGTACACTGACTAATTCAATTCCAATAAAAGTGCACAT
	gapdhs	NM_213094.2	GAGGTTTAGCACCACAATCCACACTCTTGCAATAATGTCTGAGCTTTGTGTTGGAATCAATGGATTTGGCCGTATTGGCCGTTTGGTCCTCAGGGCCTGC
	ubic	NM_001077804.1	AGATGGACGCACTCTGTCCGACTACAACATCCAGAAGGAGTCCACCCTCCACCTGGTGCTGCGTCTTCGTGGCGGCAAATAATCTGCTTATGATTAATCA
	sdha	NM_200910.1	AAGACTATGCAGAGTCACGCAGCTGTGTTCCGTACTGGAGATGTTCTGAAGGAGGGTTGTGTCAAGATGGAGTCCGTCTATAAGTCGATGGATAACATTA
	eef1a1a	NM_200009.1	CTTCGGTGCACTGACAGCTTTATGTTTAGCACTTTGACTGGCGTAGGCTAACCCTGCACAAGCACTTGTTGACTTTAATTGGCATTAGCTACTGCACCTT
**pronephric duct**	chordin	NM_130973.1	CGACTCTGGGAGGAAGGAGGTCGAGTCTCTGTTTGATTTCTTCCAGGAAAAAGATGACGATTTGCACAAATCCTACAACGACAGATCGTACATCAGCTCC
	cdh17	NM_194422.1	GCACTTTTGCTACACTTGACCCTTCTCGTCAGCATTGGTCATGGGATTGATCTAGAGGACAAAAAGGGGCCATTAATAGATACGGTTTTAGATGTGCCAG
	pax2a	NM_131184.2	CCATTCTTGAAGAGAGGGGGACTGAGAGACACTCGCCGTGTCCATGTTTTTTCCTGTCATTCCCGCACGAATCTGACTGTACCCCTCAGACTGTTCAGAC

Supplemental References

Bahary, N., Goishi, K., Stuckenholz, C., Weber, G., Leblanc, J., Schafer, C.A., Berman, S.S., Klagsbrun, M., and Zon, L.I. (2007). Duplicate VegfA genes and orthologues of the KDR receptor tyrosine kinase family mediate vascular development in the zebrafish. Blood *110*, 3627-3636.

Bertrand, J.Y., Kim, A.D., Teng, S., and Traver, D. (2008). CD41+ cmyb+ precursors colonize the zebrafish pronephros by a novel migration route to initiate adult hematopoiesis. Development *135*, 1853-1862.

Cheah, F.S., Winkler, C., Jabs, E.W., and Chong, S.S. (2010). Tgfbeta3 regulation of chondrogenesis and osteogenesis in zebrafish is mediated through formation and survival of a subpopulation of the cranial neural crest. Mech Dev *127*, 329-344.

Chocron, S., Verhoeven, M.C., Rentzsch, F., Hammerschmidt, M., and Bakkers, J. (2007). Zebrafish Bmp4 regulates left-right asymmetry at two distinct developmental time points. Dev Biol *305*, 577-588.

Clements, W.K., Kim, A.D., Ong, K.G., Moore, J.C., Lawson, N.D., and Traver, D. (2011). A somitic Wnt16/Notch pathway specifies haematopoietic stem cells. Nature *474*, 220-224.

Danilova, N., Sakamoto, K.M., and Lin, S. (2011). Ribosomal protein L11 mutation in zebrafish leads to haematopoietic and metabolic defects. Br J Haematol *152*, 217-228.

Espin-Palazon, R., Stachura, D.L., Campbell, C.A., Garcia-Moreno, D., Del Cid, N., Kim, A.D., Candel, S., Meseguer, J., Mulero, V., and Traver, D. (2014). Proinflammatory signaling regulates hematopoietic stem cell emergence. Cell *159*, 1070-1085.

Geiss, G.K., Bumgarner, R.E., Birditt, B., Dahl, T., Dowidar, N., Dunaway, D.L., Fell, H.P., Ferree, S., George, R.D., Grogan, T., *et al.* (2008). Direct multiplexed measurement of gene expression with color-coded probe pairs. Nat Biotechnol *26*, 317-325.

Gering, M., and Patient, R. (2005). Hedgehog signaling is required for adult blood stem cell formation in zebrafish embryos. Dev Cell *8*, 389-400.

Jin, S.W., Beis, D., Mitchell, T., Chen, J.N., and Stainier, D.Y. (2005). Cellular and molecular analyses of vascular tube and lumen formation in zebrafish. Development *132*, 5199-5209.

Kolde, R. (2013). pheatmap: Pretty Heatmaps. R package version 0.7.7. (http://cran.r-project.org/web/packages/pheatmap/index.html).

Link, V., Shevchenko, A., and Heisenberg, C.P. (2006). Proteomics of early zebrafish embryos. BMC Dev Biol *6*, 1.

Livak, K.J., and Schmittgen, T.D. (2001). Analysis of relative gene expression data using real-time quantitative PCR and the 2(-Delta Delta C(T)) Method. Methods *25*, 402-408.

Monteiro, R., Pouget, C., and Patient, R. (2011). The gata1/pu.1 lineage fate paradigm varies between blood populations and is modulated by tif1gamma. EMBO J *30*, 1093-1103.

Simoes, F.C., Peterkin, T., and Patient, R. (2011). Fgf differentially controls cross-antagonism between cardiac and haemangioblast regulators. Development *138*, 3235-3245.

Waggott, D., Chu, K., Yin, S., Wouters, B.G., Liu, F.F., and Boutros, P.C. (2012). NanoStringNorm: an extensible R package for the pre-processing of NanoString mRNA and miRNA data. Bioinformatics *28*, 1546-1548.

Yamamoto, M., Morita, R., Mizoguchi, T., Matsuo, H., Isoda, M., Ishitani, T., Chitnis, A.B., Matsumoto, K., Crump, J.G., Hozumi, K., *et al.* (2010). Mib-Jag1-Notch signalling regulates patterning and structural roles of the notochord by controlling cell-fate decisions. Development *137*, 2527-2537.
