# Supplementary material for: Transforming Growth Factor β Drives Hemogenic Endothelium Programming and the Transition to Hematopoietic Stem Cells
Source: Dev Cell. 2016 Aug 22;38(4):358–70. doi: 10.1016/j.devcel.2016.06.024 (PMC4998007; doi:10.1016/j.devcel.2016.06.024)
Supplement: Document S2. Article plus Supplemental Information [file mmc3.pdf]

Article

Developmental Cell

Transforming Growth Factor β Drives Hemogenic Endothelium Programming and the Transition to Hematopoietic Stem Cells

Graphical Abstract

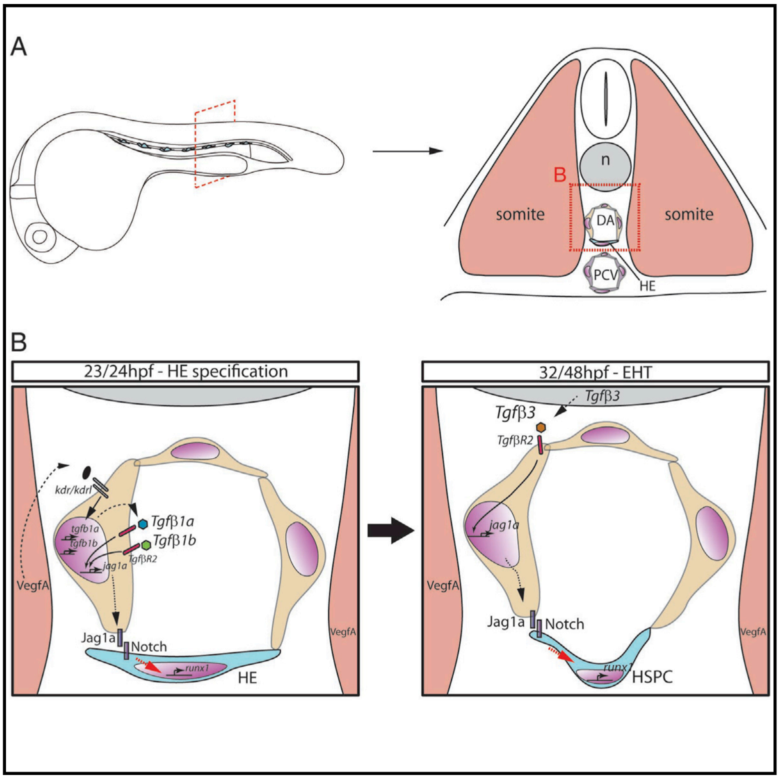

Highlights

- TGFβ signaling is required for hematopoietic stem cell (HSC) emergence in embryos
- TGFβ regulates *jag1a* expression and programs endothelium to become hemogenic endothelium (HE)
- Tgfb1a/Tgfb1b and Tgfb3 act sequentially to program HE and give rise to HSCs

Authors

Rui Monteiro, Philip Pinheiro, Nicola Joseph, ..., Florian Bonkhofer, Arif Kirmizitas, Roger Patient

Correspondence

rui.monteiro@imm.ox.ac.uk (R.M.),
roger.patient@imm.ox.ac.uk (R.P.)

In Brief

Hematopoietic stem cells (HSCs) arise in the embryo from hemogenic endothelium in the dorsal aorta. Here, Monteiro et al. reveal that sequential activity by two TGFβ ligands and crosstalk between TGFβ and Notch signaling are required to program arterial endothelial cells to become hemogenic and give rise to HSCs.

Monteiro et al., 2016, Developmental Cell *38*, 358–370
August 22, 2016 © 2016 The Author(s). Published by Elsevier Inc.
http://dx.doi.org/10.1016/j.devcel.2016.06.024

CellPress

Transforming Growth Factor β Drives Hemogenic Endothelium Programming and the Transition to Hematopoietic Stem Cells

Rui Monteiro,[1,2,4,*] Philip Pinheiro,[1] Nicola Joseph,[1] Tessa Peterkin,[1] Jana Koth,[1] Emmanouela Repapi,[3] Florian Bonkhofer,[1] Arif Kirmizitas,[1] and Roger Patient[1,2,*]
[1]Molecular Haematology Unit, Weatherall Institute of Molecular Medicine, John Radcliffe Hospital, University of Oxford, Oxford OX3 9DS, UK
[2]BHF Centre of Research Excellence, Oxford, UK
[3]Computational Biology Research Group, Weatherall Institute of Molecular Medicine, John Radcliffe Hospital, University of Oxford, Oxford OX3 9DS, UK
[4]Lead Contact
*Correspondence: rui.monteiro@imm.ox.ac.uk (R.M.), roger.patient@imm.ox.ac.uk (R.P.)
http://dx.doi.org/10.1016/j.devcel.2016.06.024

SUMMARY

Hematopoietic stem cells (HSCs) are self-renewing multipotent stem cells that generate mature blood lineages throughout life. They, together with hematopoietic progenitor cells (collectively known as HSPCs), emerge from hemogenic endothelium in the floor of the embryonic dorsal aorta by an endothelial-to-hematopoietic transition (EHT). Here we demonstrate that transforming growth factor β (TGFβ) is required for HSPC specification and that it regulates the expression of the Notch ligand Jagged1a in endothelial cells prior to EHT, in a striking parallel with the epithelial-to-mesenchymal transition (EMT). The requirement for TGFβ is two fold and sequential: autocrine via Tgfβ1a and Tgfβ1b produced in the endothelial cells themselves, followed by a paracrine input of Tgfβ3 from the notochord, suggesting that the former programs the hemogenic endothelium and the latter drives EHT. Our findings have important implications for the generation of HSPCs from pluripotent cells in vitro.

INTRODUCTION

Hematopoietic stem cells (HSCs) are specified during embryonic development from a subset of arterial endothelial cells located in the floor of the dorsal aorta (DA). HSCs emerge by a process termed the endothelial-to-hematopoietic transition (EHT) (Bertrand et al., 2010; Boisset et al., 2010; Kissa and Herbomel, 2010). In zebrafish, the hematopoietic stem and progenitor cells (HSPCs) generated by EHT migrate to the caudal hematopoietic tissue (CHT), where they proliferate and undergo differentiation into erythroid and myeloid lineages (Kissa et al., 2008; Murayama et al., 2006). Some will exit the CHT and migrate to the thymus to give rise to T cells, and others move to the kidney, the adult site of hematopoiesis in the zebrafish, equivalent to the bone marrow in mammals (Ciau-Uitz et al., 2014).

The transcription factor Runx1 is required for EHT in mice and zebrafish (Chen et al., 2009; Kissa and Herbomel, 2010). Its expression in the floor of the DA is initiated by 23 hpf in zebrafish (Wilkinson et al., 2009) and marks a cell population committed to the hemogenic fate, the hemogenic endothelium (HE). Several signaling pathways including Hedgehog, VEGF, Notch and BMP are required sequentially to regulate programming of the arterial endothelium and HSPC emergence (Burns et al., 2005; Gering and Patient, 2005; Kim et al., 2014; Wilkinson et al., 2009). The Notch receptor Notch1 is the main driver of HSPC emergence from HE, likely downstream of its ligand Jagged1 (Gama-Norton et al., 2015; Hadland et al., 2015; Jang et al., 2015) and is thought to drive *runx1* expression via Gata2 (Robert-Moreno et al., 2005). Jagged1 is dispensable for arterial programming but required in the endothelium for the specification of HSPCs (Espin-Palazon et al., 2014; Gama-Norton et al., 2015; Robert-Moreno et al., 2008).

In humans, defective transforming growth factor β (TGFβ) signaling is associated with proliferative disorders of HSPCs such as acute myeloid leukemia and T cell acute lymphoblastic leukemia (Kim and Letterio, 2003). More recently, it has been shown that paracrine TGFβ signaling in the bone marrow niche maintains quiescence of the resident HSC pool (Zhao et al., 2014) and may also direct differentiation of lineage-biased HSC subtypes (Challen et al., 2010), positioning TGFβ as a critical regulator of proliferation and differentiation of adult HSCs. Whether TGFβ plays a role in the formation of HSCs is however not known. Mutants for the ligand TGFβ1 or its receptor TGFβR2, including endothelial-specific conditional knockout mice, die between E9.5 and E10.5 due to defective recruitment of mural cells to the yolk sac vasculature and the subsequent loss of vessel integrity (Carvalho et al., 2004; Dickson et al., 1995; Oshima et al., 1996). This is before the emergence of HSPCs in the embryo proper (de Bruijn et al., 2002), effectively precluding the analysis of the role of TGFβ signaling in HSPC specification in mice. Zebrafish, however, develop externally and do not depend on extraembryonic tissues for survival. In addition, recruitment of mural cells to the endothelium does not happen until 72 hpf (Santoro et al., 2009), 2 days after the HSPCs are specified in the DA. Thus, we can address the role of TGFβ in HSPC emergence in zebrafish without the inherent limitations of the mouse models.

 © 2016 The Author(s). Published by Elsevier Inc.
This is an open access article under the CC BY license (http://creativecommons.org/licenses/by/4.0/).

The TGFβ superfamily comprises BMPs, Activins, Nodals, and TGFβs. There are three TGFβ ligands in the mouse: TGFβ1, TGFβ2, and TGFβ3 (Goumans and Mummery, 2000), and they all signal through a single type II serine-threonine kinase receptor (TGFβR2) that recruits the type I receptors Activin-like kinase 1 (Alk1) or Alk5 (Shi and Massague, 2003). Alk1 expression is essentially restricted to endothelial cells (ECs) (Oh et al., 2000), whereas Alk5 is more broadly expressed (Goumans et al., 2002) but also present in ECs. Activated Alk1 phosphorylates Smad1, Smad5, and Smad9, whereas activated Alk5 phosphorylates Smad2 and Smad3 (Shi and Massague, 2003). Activated Smads migrate to the nucleus together with the co-Smad Smad4 and regulate transcription together with co-activators or co-repressors (Shi and Massague, 2003). In addition, TGFβ can signal through the non-canonical Erk, JNK, and p38 MAPK kinase pathways to instigate transcriptional responses (Derynck and Zhang, 2003). Thus, to circumvent the complexity of the intracellular signaling elicited by TGFβ, we focused our attention on TgfβR2, the type II receptor for TGFβ. Abrogation of TGFβR2 activity revealed that TGFβ signaling plays a key role in the formation of HSPCs. TGFβ is required for the correct programming of the HE downstream of Vegf and independently of arterial programming. We demonstrate that Jag1a is a target of TGFβ signaling, and *jag1a* overexpression in endothelium rescues the loss of HSPCs in *tgfbR2*-depleted embryos. Finally, we identified two independent sources of ligand: TGFβ1a and TGFβ1b in the endothelium and TGFβ3 in the nearby notochord. Both inputs contribute to the regulation of *jag1a* in endothelium through the TgfβR2 receptor and thus enable Notch signaling to program the HE prior to specification of HSPCs.

RESULTS

TGFβ Signaling Components Are Expressed in or around the Embryonic Site of HSC Emergence

To investigate whether TGFβ signaling could play a role in HSPC specification in zebrafish, we first carried out expression analysis. *tgfβR2* is expressed in the head vasculature and in the somites at 15 hpf and in the DA and the somites from at least 18 hpf up to 24 hpf (Figures 1A and S1A), prior to the onset of *runx1* expression in the HE (Wilkinson et al., 2009). At 30 hpf, *tgfbR2* becomes essentially endothelial, with higher expression in the posterior cardinal vein (PCV) and in the caudal plexus (Figure 1A). TGFβ ligands are also expressed in the region at the onset of HE formation: *tgfb1a* and *tgfb1b* are expressed in the endothelium, including the DA at 15 hpf, 24 hpf, and 27 hpf (Figures 1B, 1C, S1B, and S1C). At 27 hpf, *tgfb1a* expression is downregulated in the DA and PCV, whereas *tgfb1b* is still clearly present (Figures 1B and 1C). T*gfb2* is expressed in the notochord at 12 hpf, 20 hpf, and 24 hpf (Figures 1D and S1D), and *tgfb3* is expressed in the notochord and in the 3–4 anterior-most somites from 12 hpf to 20 hpf and also in ECs in the head (Figures 1E and S1E). From 20 hpf onward, *tgfb3* was found in the dorsal tip of the somites, the floorplate, and in the notochord (Figure 1E).

TGFβ Signaling through TgfβR2 Is Required for the Specification of HSPCs

To investigate whether TGFβ signaling is required for the specification of HSPCs, we designed an antisense morpholino oligonucleotide (MO) targeting the start site of tgfbR2 translation (*tgfβR2*^{MO1}; Figure S2A) and verified that it decreased TgfβR2 protein levels at 26 hpf (Figure S2B). *TgfbR2* morphants showed a severe decrease in expression of *runx1*, *gfi1aa*, and *gata2b*, two other HE markers (Butko et al., 2015; Cooney et al., 2013), at 26–28 hpf (Figures 2A–2F), suggesting that HSPC emergence is impaired. Specification of the arterial program in the endothelium of the DA by Notch signaling is required for HSPC emergence (Burns et al., 2005; Gering and Patient, 2005). Therefore, we asked if either the endothelial or the arterial programs are affected by loss of TGFβ signaling. We found that the pan-endothelial *kdrl* and the arterial markers *notch3, hey2*, and *efnb2a* were unaffected in *tgfβR2* morphants (Figures 2G–2N).

HSPCs emerging from the DA express *kdrl* and low levels of *itga2b* (also known as CD41) in itga2b:GFP;Kdrl:HsRas-mCherry transgenic embryos (Kissa et al., 2008). To quantitate the loss of HSPCs in *tgfbR2* morphants, we counted the number of Kdrl-mCherry^{+};itga2b-GFP^{low} HSPCs in the DA of live itga2b:GFP;Kdrl:HsRas-mCherry transgenic embryos by confocal microscopy at 48 hpf (Figures 2O–2Q). In *tgfbR2* morphants, the number of Kdrl-mCherry^{+};itga2b-GFP^{low} HSPCs was reduced more than 2-fold compared with uninjected embryos (Figure 2Q). The loss of itga2b-GFP^{low} cells in *tgfbR2* morphants was still evident at 5 dpf (Figures 2R and 2S) and, consistent with this, we found that the expression of the HSPC markers *runx1*, *cmyb*, and *ikzf1* was severely downregulated in *tgfbR2* morphants at 48 hpf (Figure S2D). *cmyb*, *ikzf1*, and *l-plastin* (pan-leukocyte marker) were severely reduced in the CHT and in the thymus at 4 dpf (Figures S2E and S2F). Expression of the early T cell marker *rag1* in the thymus (Figures 2T and 2U) and the erythroid marker *hbbe1* in the CHT (Figures 2V and 2W) were also severely reduced. Taken together, these experiments indicate that TGFβ signaling is required for the specification of HSPCs.

We designed a second MO, *tgfbR2*^{MO2} that blocks splicing of exon 4 of *tgfβR2* (Figure S2A), and confirmed the results obtained with *tgfbR2*^{MO1} (Figure S2G). Neither the pan-endothelial marker *fli1* nor the arterial markers *dll4* and *dlC* were affected in *tgfbR2*^{MO1} and *tgfbR2*^{MO2} morphants (Figure S2G). To examine whether TGFβ signaling is also required for primitive hematopoiesis, we performed in situ hybridization for *scl*, *gata1*, and *pu.1* at 20 hpf (Figure S2H) and *gata1* and *pu.1* at 24 hpf (Figure S2I). *TgfbR2* morphants showed no significant change in expression of these markers, suggesting that specification of primitive hematopoietic cells does not require TGFβ signaling through TgfβR2. However, maturation of primitive erythrocytes was slightly impaired, as suggested by a small decrease in *o*-dianisidine staining in *tgfbR2* morphants at 36 hpf (Figure S2J).

Taken together, our data show that TGFβ signaling through TgfβR2 is required for the specification of HSPCs independently of arterial programming.

TGFβ1 in the Arterial Endothelium and TGFβ3 from the Notochord Are Required for HSPC Emergence

Tgfβ1$^{-/-}$ and TgfβR2$^{-/-}$ mouse mutants share a similar vasculogenic phenotype in the yolk sac (Dickson et al., 1995; Oshima et al., 1996); thus we reasoned that TGFβ1 was the likeliest ligand for TgfbR2 in HSPC emergence. To test this hypothesis, we knocked down *tgfb1a* or *tgfb1b* with at least two

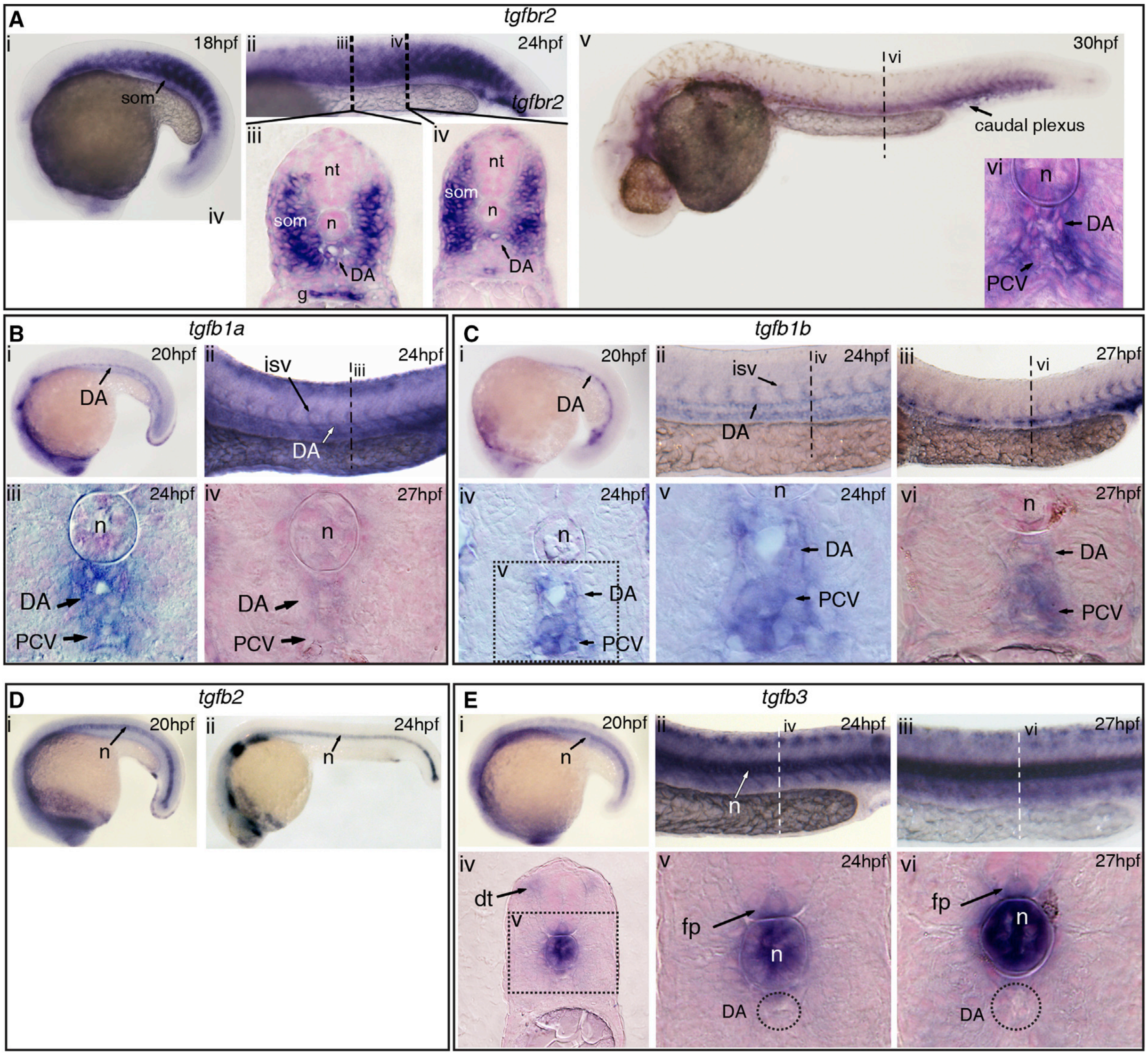

Figure 1. TGFβ Signaling Components Are Expressed in and around the Embryonic Dorsal Aorta

(A) Expression of *tgfbR2* at (i) 18 hpf and (ii–iv) 24 hpf, including the somites, dorsal aorta (DA), and gut. (v–vi) At 30 hpf, expression was confined to the DA, notochord, posterior cardinal vein (PCV), and some of the surrounding mesenchyme.

(B) Expression of *tgfb1a* in the DA at (i) 20 hpf and (ii, iii) in the DA, PCV, and intersomitic vessels (ISVs) at 24 hpf. At 27 hpf, there was very little expression of *tgfb1a* remaining in the DA.

(C) *tgfb1b* is also expressed in the DA (i) at 20 hpf and in the DA and PCV at (ii) 24 hpf and (iii) 27 hpf. (iv, v) Transversal sections show *tgfb1b* expression at 24 hpf in the DA and PCV. (vi) *Tgfb1b* was still apparent in the DA and PCV by 27 hpf.

(D) Expression of *tgfb2* at (i) 20 hpf and (ii) 24 hpf. Notochord-specific expression was found throughout all the stages analyzed.

(E) Expression of *tgfb3* at (i) 20 hpf, (ii) 24 hpf, and (iii) 27 hpf. (iv, v) Transversal section at 24 hpf, showing expression in the dorsal tip of the somites, notochord, and floorplate. (vi) Expression in the notochord and floorplate was maintained at 27 hpf. Note that *tgfb3* is absent from the DA.

g, gut; dt, dorsal tip of the somite; fp, floorplate; isv, intersomitic vessel; n, notochord; nt, neural tube; som, somite. See also Figure S1.

splice-blocking morpholinos for each (Figures S3A–S3F) and found partial loss of *runx1* and *cmyb* in the DA without affecting expression of the arterial marker *dll4* (Figure S3F and data not shown). Co-injection of half the amounts of $tgfb1a^{MO2}$ and $tgfb1b^{MO2}$ (7.5 + 10 ng, respectively, referred to as $tgfb1^{MO2}$) induced a severe loss of *runx1* and *cmyb* expression in a higher proportion of embryos at 28 hpf when compared with single *tgfb1a* or *tgfb1b* morphants (Figures 3A, S3E, and S3F; and results not shown), suggesting that, in single morphants, the TGFβ1 ligands can partially compensate for the other's absence and that both are required for HSPC emergence. Knocking down *tgfb2* with a splice-blocking morpholino ($tgfb2^{MO3}$; Figure S3G) had very little effect on *runx1* expression (Figure 3A), whereas over half (28/50) of the *tgfb3* morphants ($tgfb3^{MO2}$, Figure S3H) showed a severe decrease in *runx1* in the DA at 28 hpf (Figure 3A). Expression of *kdrl* in the endothelium and *dll4* and *dlC* in the arterial endothelium was unaffected in $tgfb1^{MO2}$, $tgfb2^{MO3}$, or $tgfb3^{MO2}$ morphant embryos

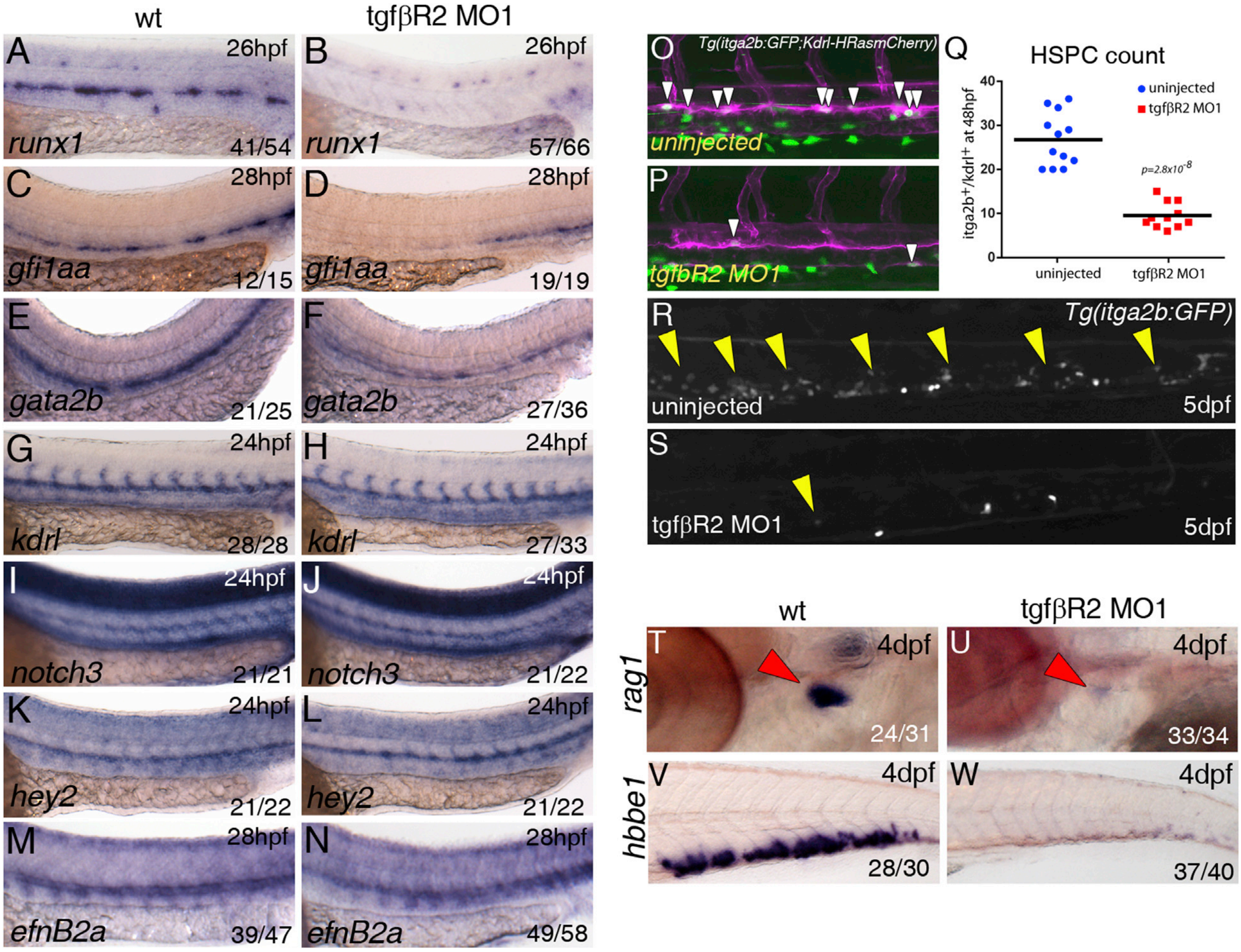

Figure 2. TGFβ Signaling Is Required for Specification of HSCs

Expression of *runx1* in (A) wild-type (wt) and (B) *tgfbr2* morphants at 26 hpf. Expression of *gfi1aa* in (C) wild-type or (D) *tgfbr2* morphants at 26 hpf. Expression of *gata2b* in (E) wild-type or (F) *tgfbr2* morphants at 26 hpf.

Expression of the vascular marker *kdrl* (G, H) and the arterial markers *notch3* (I, J), *hey2* (K, L), and *efnB2A* (M, N) is unaffected in *tgfbR2* morphants. Maximum projections of itga2b:GFP; Kdrl:HRas-mCherry transgenic embryos at 48 hpf in (O) uninjected and (P) *tgfbr2* MO1-injected embryos. Region shown includes part of the DA and white arrowheads denote itga2b:GFP$^+$(green), kdrl:HRas-mCherry$^+$ (magenta) HSPCs.

(Q) HSPC counts in the entire trunk region of uninjected and *tgfbR2* morphant itga2b:GFP; Kdrl:HRas-mCherry transgenic embryos at 48 hpf (p value is indicated on the graph, n = 12 (wild-type) and n = 11 (*tgfbR2*^{MO1}).

itga2b-GFP$^+$ cells are present in the CHT of itga2b:GFP embryos (R, yellow arrowheads) and greatly reduced in the CHT of *tgfbR2* morphants at 5 dpf (S). Expression of *rag1* in the thymus (red arrowheads) at 4 dpf in (T) wild-type and (U) *tgfbR2* morphants. Expression of *hbbe1* in the CHT at 4 dpf in (V) wild-type and (W) *tgfbR2* morphants.

The numbers of embryos are shown in each panel as the number of embryos with phenotype/total number analyzed. See also Figure S2.

(Figures 3B–3D), consistent with TGFβ signaling being required for HSPC specification but not for arterial programming. To quantitate the effect, we counted the number of Kdrl-mCherry$^+$;itga2b-GFP^{low} HSPCs at 48 hpf (Figures 3E–3H). Both *tgfb1*^{MO2} and *tgfb3*^{MO2} morphants showed severely reduced numbers of HSPCs when compared with uninjected embryos. *tgfb3*^{MO2} morphants had fewer HSPCs than *tgfb1*^{MO2} morphants at 48 hpf (Figure 3H), which correlated with a stronger decrease in *rag1* expression in the thymus of *tgfb3*^{MO2} morphants at 4 dpf (Figures 3I–3K). Further analysis revealed that expression of the arterial marker *efnB2a* was unaffected, whereas that of the HE marker *gata2b* was reduced in *tgfb1*^{MO2} and in half of the *tgfb3*^{MO2} embryos (Figures 3L and 3M). Next we investigated whether the milder phenotype in *tgfb3* morphants was due to upregulation of Tgfb1. We found that *tgfb3* was essentially absent in tgfb3^{MO2} morphants but *tgfb1a* or *tgfb1b* expression was unaffected (Figure S3I). Conversely, *tgfb1* morphants showed increased *tgfb3* expression in the notochord (Figure S3I). Knocking down *tgfb1* in *tgfb3* morphants increased the percentage of embryos with reduced *runx1* expression from 50% to 85% (Figures S3J and S3K), suggesting that Tgfβ1 and Tgfβ3 have an additive effect on HSPC specification. Taken together, we conclude that Tgfβ1a/1b produced by the ECs of the DA are required for HSPC formation by programming the HE downstream or in parallel to arterial programming. In addition, there is a significant

paracrine contribution by Tgfβ3, which becomes a more important regulator of HSPC generation between 28 and 48 hpf.

Vegf Signaling Regulates Expression of the *tgfb1a* and *tgfb1b* Ligands in the Dorsal Aorta

The sequential activity of VegfA and Notch is required for programming the DA endothelium to become arterial and give rise to HSPCs (Burns et al., 2005; Gering and Patient, 2005; Leung et al., 2013). Because our data suggest that the requirement for TGFβ lies downstream or parallel to arterial programming by Notch signaling (Figures 1, 2, and 3), we asked whether Vegf or Notch signaling might act as upstream transcriptional regulators of TGFβ ligands. To address this, we treated wild-type embryos after gastrulation with selective inhibitors for Vegf (DMH4, 20 μM) and Notch signaling (DAPM, 100 μM) (Hao et al., 2010; Walsh et al., 2002) and examined the expression of *tgfb1a*, *tgfb1b*, *tgfb3*, and *tgfbR2* (Figure 4). DMH4-treated embryos failed to form intersomitic vessels as expected (Bahary et al., 2007) and showed diminished *kdrl* expression in the trunk vasculature when compared with DMSO-treated controls (Figure 4A). The DAPM treatment had no effect on *kdrl* expression (Figure 4A). Blocking either Vegf or Notch signaling led to loss of *runx1* from the floor of the DA by 28 hpf, as described (Burns et al., 2005; Gering and Patient, 2005; Lam et al., 2010) (Figure 4B). To ask whether the loss of *kdrl* expression upon inhibition by DMH4 was due to transcriptional regulation by Vegf, we repeated the experiment in Tg(Fli1:EGFP) embryos. We confirmed that intersomitic vessels were absent but trunk ECs were still present in DMH4-treated embryos (Figure 4C). Analysis by qPCR showed that *kdrl* was decreased in DMH4-treated Fli-EGFP$^+$ ECs (Figure 4D). Strikingly, inhibition by DMH4 led to decreased *tgfb1a* and *tgfb1b* in the endothelium, whereas DAPM treatment had no obvious effect (Figures 4D and 4E). *Tgfb3* and *tgfbR2* were unaffected by either treatment, suggesting that only *tgfb1a* and *tgfb1b* are Vegf-dependent. These results were confirmed by morpholino knockdown of the Vegf receptors, *kdr* and *kdrl* (Figure S4A). Next we asked whether Wnt16 and BMP4, which are required for HSPC formation independently of Vegf or Notch signaling in the endothelium (Clements et al., 2011; Wilkinson et al., 2009), could be upstream regulators of TGFβ. Knocking down either Wnt16 or BMP4 had no effect on TGFβ ligand or receptor expression (Figure S4B). Thus, we conclude that Vegf signaling is an upstream regulator of TGFβ signaling by positively regulating expression of *tgfb1a* and *tgfb1b* in ECs (Figure 4F) before HSPC specification.

The Notch Ligand Jag1a Is a Downstream Target of TGFβ Signaling in Endothelial Cells

Formation of HSPCs requires many cell extrinsic and intrinsic factors (ligands, receptors, transcription factors, and chromatin modifiers). Thus, to investigate whether any of the known pathways required to specify HSPCs are regulated by TGFβ signaling, we used the NanoString gene quantitation system (Geiss et al., 2008). We designed a custom panel of 132 NanoString probes that included Vegf, Notch, BMP, Wnt, Hh, and TGFβ signaling pathway components or targets. The probe set also contained known blood and endothelial genes, cell-cycle and apoptosis genes, mediators of EMT, and six housekeeping genes for data normalization. To assess expression changes in the somites as well as in the endothelium, we dissected the trunks of wild-type and *tgfbR2* morphant embryos at 26 hpf and isolated total RNA to hybridize against the NanoString probe set (Figure 5A). Only nine of the genes probed showed statistically significant differences in expression ($p < 0.05$ and an absolute logFC >0.5) between wild-type and *tgfbR2* morphants, importantly including decreased *runx1* expression in the morphants (Figure 5B and Table S1). Applying a more stringent filtering (false discovery rate <0.1) yielded a smaller high-confidence subset of differentially expressed genes in *tgfbR2* morphants (Figure 5C). Five of six genes in this subset were upregulated and three of those, *p53*, *cdkn1a*, *bax*, are associated with apoptosis and cell-cycle arrest (Menendez et al., 2009). A fourth gene, *rspo1*, is an agonist of Wnt signaling that is required for sprouting angiogenesis (Gore et al., 2011) and is expressed at very low levels in wild-type embryos (Table S1). *Taz*, a Wnt signaling mediator (Azzolin et al., 2012), was also upregulated in our assay, suggesting a link between TGFβ and Wnt signaling. However, when we sorted kdrl:GFP$^+$ ECs versus kdrl:GFP$^-$ cells from control embryos and *tgfbR2* morphants (Figure 5D), we found no significant difference in *rspo1* expression by qPCR in either population (Figures 5E and 5F). Analysis of *p53*, *cdkn1a*, and *bax* expression by qPCR showed that only *p53* and *cdkn1a* were significantly upregulated in ECs (Figure 5E), whereas all three were upregulated in kdrl:GFP$^-$ cells (Figure 5F). These results suggested that *tgfbR2* morphants might show increased apoptosis. Thus we performed a TUNEL assay for apoptotic cells in Kdrl:GFP transgenic embryos and found a marked increase in TUNEL$^+$ (apoptotic) cells in the trunk and tail regions of 30 hpf *tgfbR2* morphants compared with control embryos (Figures 5G and 5H). We found increased apoptosis in ECs in the tail vascular plexus (Figure 5H′, white arrows) but not in the trunk vasculature (Figure 5H) where HSPCs arise. Thus, if *p53* and its targets *cdkn1a* and *bax* play a role in HSC specification downstream of TGFβ, it appears to be independent of their pro-apoptotic activity. The increase in *p53* could have been non-specific due to the injection of MOs, as previously reported (Robu et al., 2007). However, knocking down *runx1* also led to an increase in *p53*, *cdkn1a*, and *baxa* (Figure S5K). This raises the possibility that the increase in pro-apoptotic gene expression in *tgfbR2* morphants could be indirect, acting downstream of Runx1.

Strikingly, *jag1a* was the only gene besides *runx1* that was significantly downregulated in *tgfbR2* morphants (Figures 5B and 5C). Neither its paralog *jag1b* nor any of the other Notch ligands or receptors in the probe set were significantly affected by loss of TGFβ signaling (Figure S5A). To confirm that *jag1a* was downregulated in the absence of TGFβ signaling, we injected *tgfbR2*^{MO1} into Kdrl:GFP embryos, sorted GFP$^+$ ECs and GFP$^-$ cells, and assayed *jag1a* expression by qPCR. *jag1a* was downregulated in both populations (Figures 5I and 5J). *dll4* and *gata2a* expression was unaltered in ECs from *tgfbR2* morphants (Figure S5L), confirming the NanoString results. To determine which TGFβ ligand regulates *jag1a*, we assayed its expression in *tgfb1*^{MO2} and *tgfb3*^{MO2} morphants compared with wild-type embryos at 26 hpf. *Jag1a* was downregulated in both t*gfb1*^{MO2} and *tgfb3*^{MO2} morphants (Figure S5M). Moreover, *tgfb1*^{MO2} and *tgfb3*^{MO2} morphants showed increased *p53* and

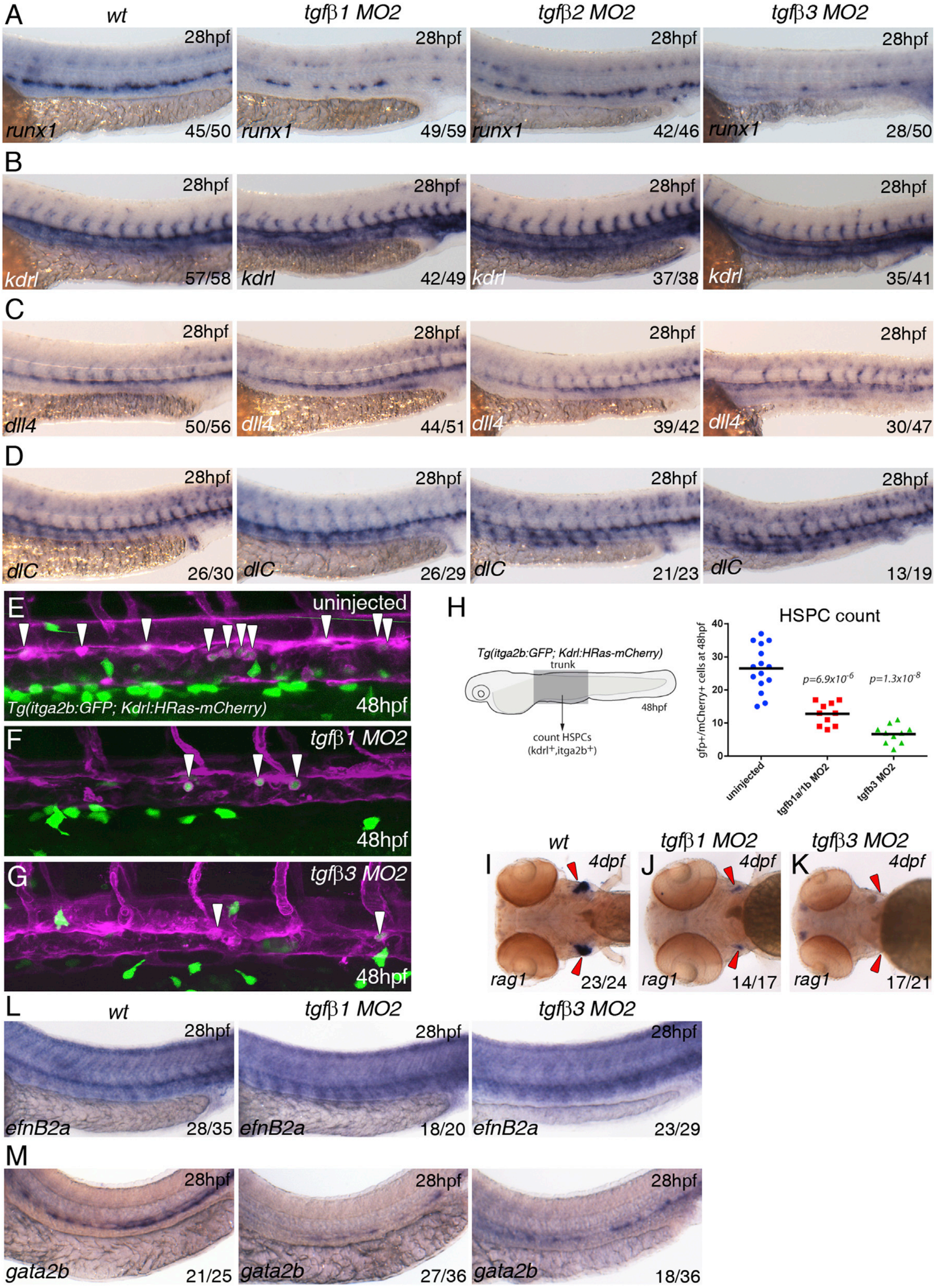

Figure 3. TGFβ1 and TGFβ3 Are Required for Specification of HSCs

(A) Expression of *runx1* in wild-type, *tgfb1, tgfb2*, and *tgfb3* morphants.

(B) Expression of *kdrl* in wild-type, *tgfb1, tgfb2*, and *tgfb3* morphants.

(legend continued on next page)

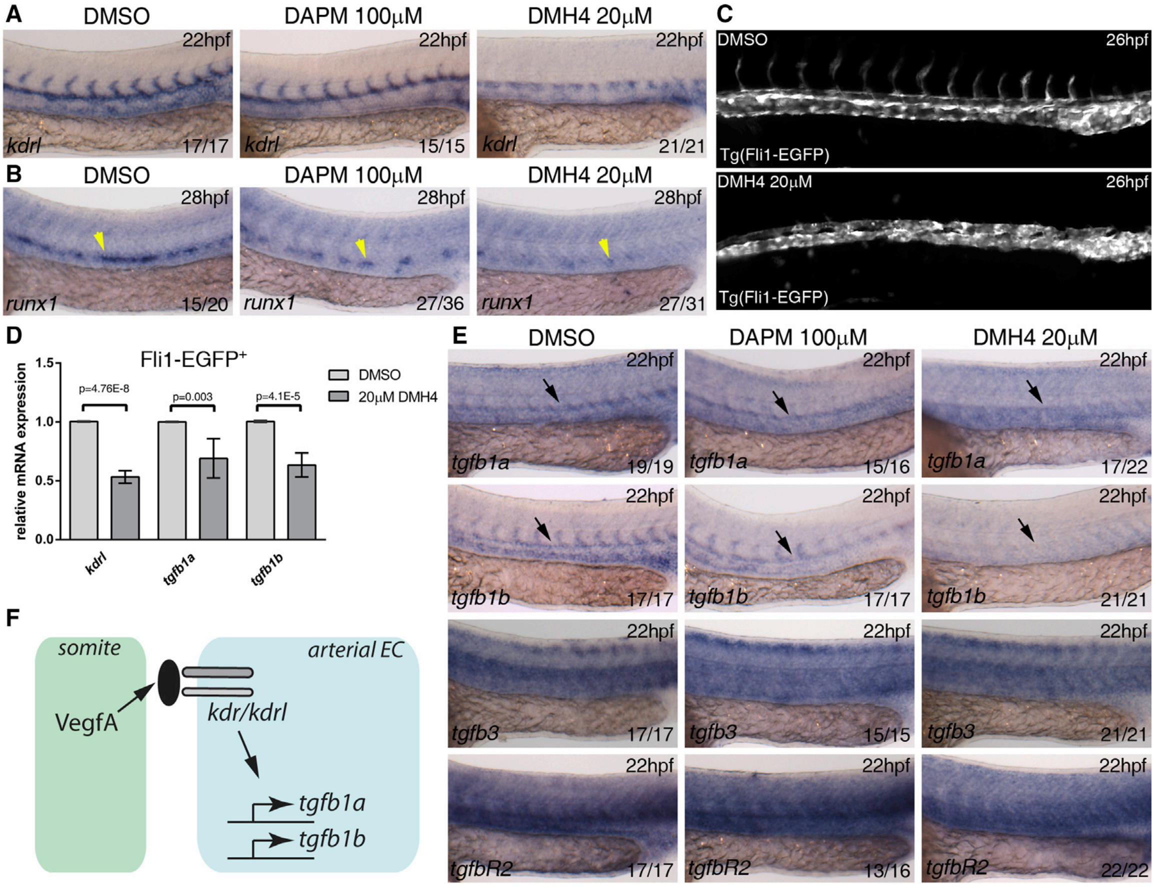

Figure 4. Vegf Signaling Is Required for *tgfb1a* and *tgfb1b* Expression in the Dorsal Aorta

(A and B) Wild-type embryos were treated from 10 hpf with DMSO (control), Vegf inhibitor DMH4 (20 μM), and Notch inhibitor DAPM (100 μM) and collected at 22 hpf or 28 hpf. Embryos were collected and analyzed for (A) *kdrl* expression at 22 hpf and (B) *runx1* expression at 28 hpf.

(C) Tg(Fli1:EGFP) embryos were treated from 10 to 26 hpf with DMSO or DMH4 (20 μM). DMH4-treated embryos showed a severe loss of intersomitic vessels but ECs are still present in the trunk, and circulation was detected in a majority of embryos at 48 hpf (data not shown).

(D) Expression of *kdrl*, *tgfb1a*, *tgfb1b* by qPCR in 26 hpf sorted Fli1:EGFP^{+} ECs. All three genes were downregulated after DMH4 treatment. Results are shown as averages ± SD of 4–5 biological replicates.

(E) Wild-type embryos were treated from 10 hpf with DMSO (control), Vegf inhibitor DMH4 (20 μM), and Notch inhibitor DAPM (100 μM) and collected at 22 hpf for analysis of *tgfb1a*, *tgfb1b*, *tgfb3*, and *tgfbR2* by in situ hybridization at 22 hpf.

(F) Schematic representation of the experimental results.

Black arrows indicate the location of the DA; yellow arrowheads indicate the location of *runx1* expression in the floor of the DA. The numbers of embryos are shown in each panel as the number of embryos with phenotype/total number analyzed. Arterial EC, arterial endothelial cell.

See also Figure S4.

no effect on *gata2a* or *dll4* expression (Figure S5N). Thus, our data indicate that *jag1a* is a TGFβ target in the endothelium at the onset of HSPC specification and suggest that both TGFβ1 and TGFβ3 contribute to the expression of *jag1a*.

Jag1a Is Required Downstream of TGFβ to Program the HE

To determine if Jag1a is required for arterial programming or HSPC specification in zebrafish, we knocked down *jag1a*

(C) Expression of *dll4* in wild-type, *tgfb1, tgfb2*, and *tgfb3* morphants.

(D) Expression of *dlC* in wild-type, *tgfb1, tgfb2*, and *tgfb3* morphants. All samples were analyzed at 28 hpf.

(E–G) Maximum projections of itga2b:GFP; Kdrl:HRas-mCherry transgenic embryos in (E) uninjected, (F) *tgfb1* morphants, and (G) *tgfb3* morphants at 48 hpf. The images show part of the trunk DA and white arrowheads denote itga2b:GFP^{+} (green), kdrl:HRas-mCherry^{+} (magenta) HSPCs.

(H) HSPCs counts in the entire trunk region of uninjected, *tgfb1*, and *tgfb3* morphant itga2b:GFP; Kdrl:HRas-mCherry transgenic embryos at 48 hpf (p value is indicated on the graph, n = 10 for each of the conditions).

(I–K) Expression of *rag1* in the thymus at 4 dpf (red arrowheads) in (I) wild-type, (J) *tgfb1* morphants, and (K) *tgfb3* morphants.

(L) Expression of *efnB2a* in wild-type, *tgfb1*, and *tgfb3* morphants.

(M) Expression of *gata2b* in wild-type, *tgfb1*, and *tgfb3* morphants. The numbers of embryos are shown in each panel as the number of embryos with phenotype/total number analyzed.

See also Figure S3.

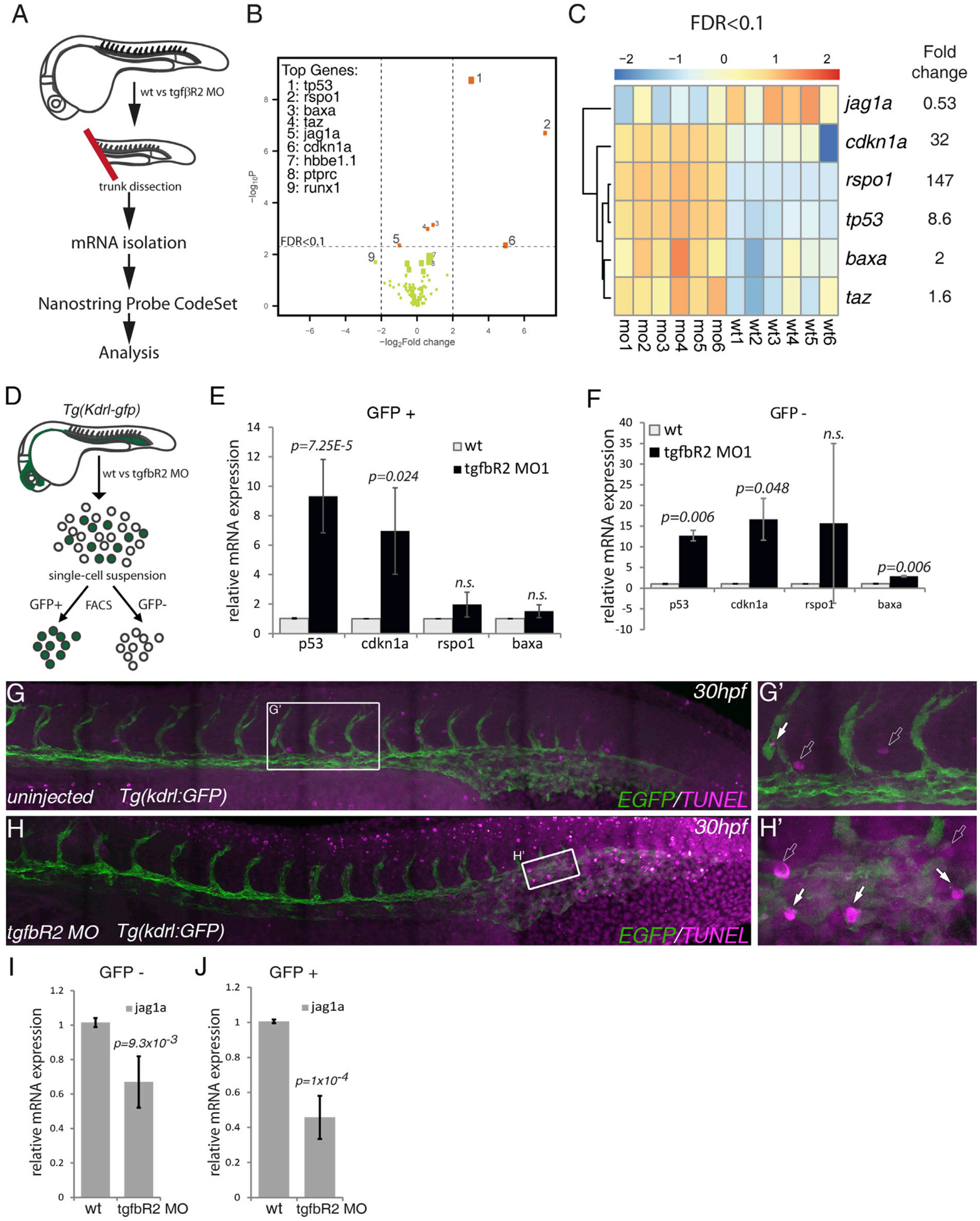

Figure 5. Multiplex Analysis of Gene Expression Shows that *jag1a* Is a Downstream Target of TGFβ Signaling

(A) Schematic representation of the trunk dissection experiment for isolation of mRNA for hybridization with the NanoString Probe CodeSet. Six groups of independent wild-type (wt1–6) and *tgfbR2*^{MO1}-injected embryos (mo1-6) were used in this analysis.

(B) Volcano plot depicting differential gene expression between wild-type and *tgfbR2* morphants in log2-fold change with a significance level of $p < 0.05$. Vertical broken lines limit the absolute logFC larger than 0.5-fold change range, whereas the horizontal broken line represents the false discovery rate (FDR) threshold set at FDR < 0.1. The genes where FDR < 0.1 are shown as orange dots. The size of the dots is proportional to mRNA expression levels.

(legend continued on next page)

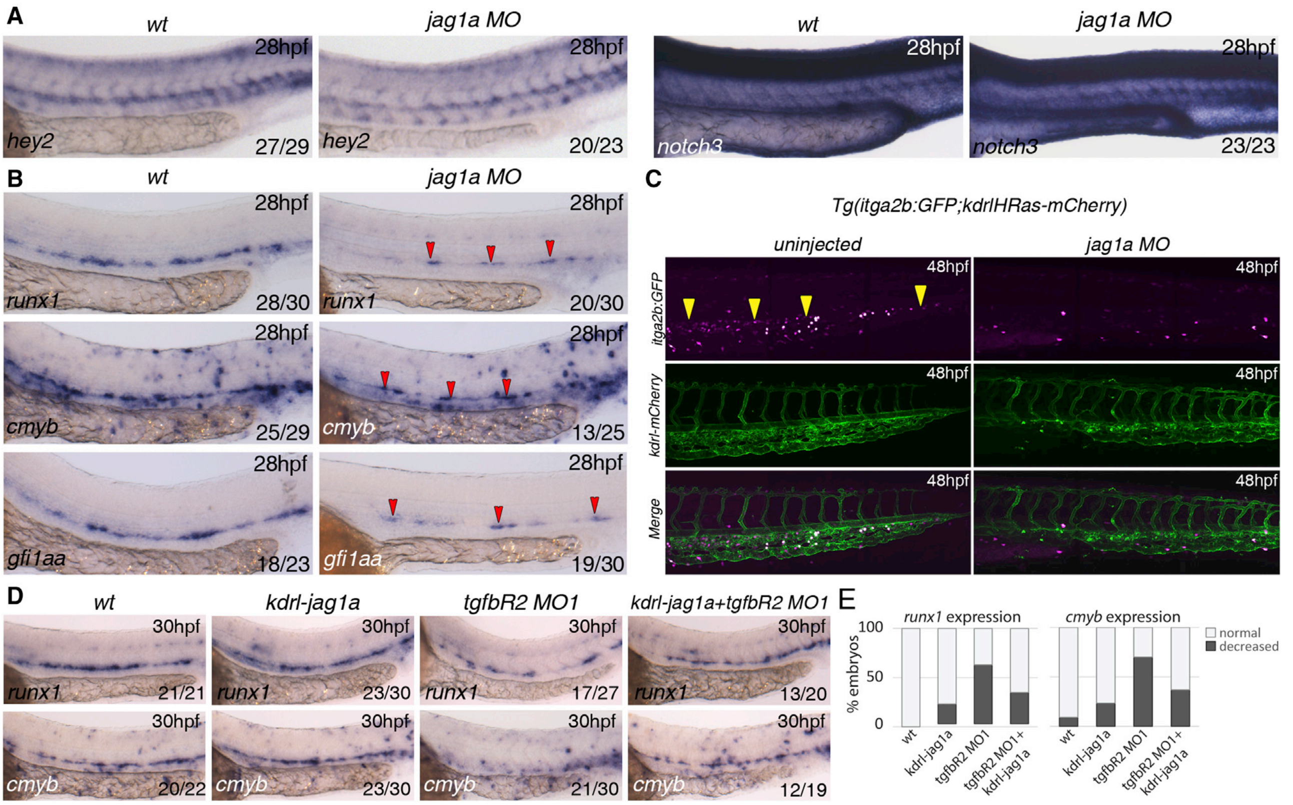

Figure 6. Jag1a Is Required Downstream of TGFβ Signaling for HSC Specification

(A) Expression of arterial markers in *jag1a*^{MO} is unaffected when compared to wild-type embryos.

(B) Expression of *runx1*, *cmyb*, and *gfi1aa* in wild-type and *jag1a* morphants (MO) at 28 hpf. All the markers analyzed are reduced or absent in *jag1a* morphants. Red arrowheads indicate the remaining gene expression in the floor of the DA of *jag1a* morphants.

(C) HSPCs (yellow arrowheads) are severely reduced in the CHT of itga2b:GFP;Kdrl:HsRas-mCherry transgenic embryos at 48 hpf injected with the *jag1a*^{MO}. Itga2b:GFP$^+$ cells, magenta; Kdrl-HsRas:mCherry$^+$ cells, green.

(D) Overexpression of *jag1a* with a Kdrl:jag1-V5 construct partially rescues the loss of *runx1* and *cmyb* in the floor of the DA. 15 pg of the construct was used for this experiment. The numbers of embryos are shown in each panel as the number of embryos with phenotype/total number analyzed.

(E) Quantitation of the rescue effect observed in (D).

See also Figure S6.

with a specific morpholino (Yamamoto et al., 2010) and found no obvious defects in arterial programming compared with wild-type embryos (Figure 6A). However, expression of the HSPC markers *runx1*, *cmyb*, and *gfi1aa* was severely downregulated in *jag1a* morphants at 28 hpf (Figure 6B). Furthermore, itga2b:GFP$^+$ HSPCs were nearly absent in the CHT of *jag1a* morphants by 48 hpf (Figure 6C) suggesting that the HE was mis-programmed and failed to give rise to HSPCs in the absence of Jag1a. A recent study showed that *jag1a* is regulated by TNFα through the TnfR2 receptor in ECs (Espin-Palazon et al., 2014). Expression of tnfR2 in ECs was unaffected in *tgfbR2* morphants (Figure S6), suggesting that TGFβ does not regulate *jag1a* indirectly via regulation of *tnfr2*. To determine if Jag1a is the main target of TGFβ signaling in HSPC specification, we restored Jag1a expression specifically in the endothelium of *tgfbr2*^{MO1} morphants using a Kdrl:jag1a construct. Wild-type embryos overexpressing *jag1a* in ECs showed little effect on expression of *runx1* or *cmyb* at 28 hpf (Figures 6D

(C) Hierarchical clustering of genes expressed with FDR < 0.1 in each of the six biological replicates analyzed (wild-type, wt1 to wt6; *tgfbR2*^{MO1}, mo1 to mo6). Results are normalized and presented as *Z* scores from −2 (downregulated) to 2 (upregulated).

(D) Schematic representation of the sorting of kdrl:GFP$^+$ cells in wild-type and *tgfbR2* morphants (MO) by fluorescence-activated cell sorting to isolate mRNA and validate the NanoString results by qPCR.

(E and F) qPCR of *p53*, *cdkn1a*, *rspo1*, and *bax* in (E) kdrl:GFP$^+$ cells and (F) kdrl:GFP$^-$ cells of wild-type and *tgfbR2* morphants (MO) at 28 hpf. *Taz* was omitted from the analysis as its fold induction <2.

(G and G′) TUNEL-stained apoptotic cells in uninjected (control) kdrl:GFP embryos at 30 hpf.

(H and H′) Apoptotic cells in *tgfbR2*^{MO1}-injected kdrl:GFP embryos at 30 hpf. White arrows, apoptotic endothelial cells; outline arrows, apoptotic non-endothelial cells.

(I and J) qPCR for *jag1a* in (I) kdrl:GFP$^-$ cells and (J) in kdrl:GFP$^+$ cells at 28 hpf.

See also Figure S5 and Table S1.

and 6E). However, forced expression of *jag1a* in the endothelium of *tgfbR2* morphants rescued the loss of *runx1* and *cmyb* expression (Figures 6D and 6E), confirming that the hematopoietic defects in *tgfbR2* morphants are mainly due to loss of *jag1a*. We conclude that autocrine TGFβ1 and paracrine TGFβ3 signal to the endothelium through TgfβR2, inducing *jag1a* expression, which in turn induces HE programming and HSPC emergence.

DISCUSSION

TGFβ Is a Regulator of HSPC Specification in the Embryo

We have demonstrated a critical role for TGFβ signaling in the specification of HSPCs. Our data show that knockdown of the type II receptor for TGFβ leads to the loss of HSPCs and their differentiated progeny. Cell-autonomous Notch signaling is required for the programming of arterial identity in the endothelium (Quillien et al., 2014) and failure to acquire this identity, through the absence of Notch signaling or Hey2, leads to loss of HSPCs (Gering and Patient, 2010; Kim et al., 2014; Rowlinson and Gering, 2010). However, recent publications suggest that arterial identity is not an absolute requirement for HE specification and HSPC emergence (Ditadi et al., 2015; Jang et al., 2015). Here we show that neither Hey2 nor the Notch pathway components that program the artery are affected by the absence of TGFβ signaling, whereas the HE markers *gata2b*, *runx1*, and *gfi1aa* are strongly downregulated. Thus, we propose that TGFβ functions independently of arterial development to program the arterial ECs to become hemogenic.

Parallel Activation of Notch and TGFβ Signaling by Vegf Programs the HE

Vegf and TGFβ are important regulators of vasculogenesis and angiogenesis in both embryonic development and cancer progression (Holderfield and Hughes, 2008), and crosstalk between them has been demonstrated, mainly through regulation of *vegfA* by TGFβ (Massague and Gomis, 2006). Here we show the opposite: expression of *tgfb1a* and *tgfb1b* ligands is dependent on VegfA signaling through its receptors Kdr and Kdrl. Vegf also regulates the expression of *hey2, notch3*, and *notch1b*, which are required for arterial programming (Gering and Patient, 2005; Lawson et al., 2002; Rowlinson and Gering, 2010). Thus, we propose that HSPC emergence requires parallel activation of both pathways by Vegf, where Notch signaling provides the arterial identity and TGFβ programs the endothelium to become hemogenic.

TGFβ and Notch Crosstalk in EHT: Similarities to Epithelial-to-Mesenchymal Transition

Because *tgfbR2* is expressed in the DA prior to HSC specification (Figures S1 and S2), we propose that TGFβ ligands act directly on ECs, resulting in *jag1a* activation. Jag1a then activates the Notch receptor, presumably Notch1a (Espin-Palazon et al., 2014), and the signal-receiving cell becomes hemogenic by expressing specific markers such as *gata2b*, *runx1*, and *gfi1aa*. Loss of TGFβ signaling would therefore prevent HE from being specified by the Jag1a/Notch1a interaction. Thus, the concerted activities of TGFβ and Notch signaling explain how only some of the ECs in the floor of the aorta are programmed to become hemogenic. Interestingly, *jag1* expression is also induced by TGFβ prior to EMT and is required for epithelial cells to progress to the mesenchymal fate in oncogenic transformation (Zavadil et al., 2004). In development, the cardiac cushion arises by an endothelial-to-mesenchymal transition (EndoMT) and this is also dependent on crosstalk between TGFβ and Notch signaling (Lamouille et al., 2014). Thus, this crosstalk between TGFβ and Notch signaling is a shared feature between EMT, EndoMT, and EHT. The similarity between these processes may guide future studies on the molecular and cellular basis of EHT.

TGFβ1a, TGFβ1b, and TGFβ3 Are Required Sequentially to Generate HSPCs

Genetic studies in mice suggested that paracrine TGFβ is primarily required to recruit smooth muscle cells to the endothelium (Pardali et al., 2010). In addition, autocrine signaling in ECs is important to regulate proliferation and migration (Pardali et al., 2010). Thus, TGFβ acts both in an autocrine and paracrine fashion in vivo. Similarly, here we describe two independent sources of TGFβ ligands that are required for HSPC specification: TGFβ1a and TGFβ1b in the endothelium, and TGFβ3 from the neighboring notochord. Our data suggest that TGFβ3 is less important for programming of HE but may instead play a more important role in the EHT process. In agreement with this, in situ hybridization for HSPC derivatives at 4 dpf showed a more severe phenotype in *tgfb3* morphants than in *tgfb1* morphants. That TGFβ3 has a role in hematopoiesis was surprising because mouse TGFβ3 mutants have no described hematopoietic phenotypes (Goumans and Mummery, 2000). However, TGFβ3$^{-/-}$ mouse embryos show loss of palatal fusion due to defective EMT (Kaartinen et al., 1995; Proetzel et al., 1995). TGFβ3 induces EMT in palate epithelial cells by downregulating E-cadherin and upregulating fibronectin and vimentin (Nawshad et al., 2007). This raises the possibility that TGFβ may be required sequentially to generate HSPCs: TGFβ1 is required for the initial HE programming, and then TGFβ3 modulates expression of extracellular matrix components to allow HE cells to undergo EHT.

Knowledge of how ECs are programmed to become HSCs is critical to inform attempts to generate these cells in vitro for therapeutic purposes. Our findings show that TGFβ signaling is required to program the HE that will give rise to HSPCs. By contrast, we have previously shown that in *Xenopus laevis* excessive TGFβ signaling blocks specification of the hemangioblast population that precedes the formation of HE (Nimmo et al., 2013). Similarly, adding TGFβ2 to Pre-HPCs, a population of primitive hematopoietic precursor cells (Ve-Cad^{+}, CD41^{+}), impairs the EHT process in vitro (Vargel et al., 2016). This suggests that primitive hematopoiesis is sensitive to elevated levels of TGFβ signaling. Whether excessive TGFβ hinders EHT from the embryonic HE that gives rise to definitive HSPCs remains to be determined. Our work highlights the importance of identifying the different spatial and temporal requirements for TGFβ signaling in the formation of HSCs and will help to realize the goal of generating HSCs in vitro for regenerative medicine.

EXPERIMENTAL PROCEDURES

Ethics Statement
All animal experiments were performed under a Home Office Licence according to the Animals Scientific Procedures Act 1986, UK, and approved by the local ethics committee.

Fish Breeding and Maintenance
Wild-type, Tg(kdrl:GFP)^{s843} (Jin et al., 2005), Tg(itga2b:GFP)^{la2} (Lin et al., 2005), Tg(Kdrl-HsRas-mCherry)^{s896} (Bertrand et al., 2010), Tg(Fli1-GFP)y1Tg (Lawson and Weinstein, 2002), and Tg(cmyb:GFP)^{zf169Tg} (North et al., 2007) fish were bred, maintained, and staged as described (Westerfield, 2000). Tg(itga2b:gfp; Kdrl-HsRas-mCherry) animals were generated by natural mating.

Morpholinos and RNA and DNA Injections
Antisense MOs (GeneTools) were used to target *runx1* (Gering and Patient, 2005), *tgfβ3* (*tgfb3^{MO2}*) (Cheah et al., 2010), *kdr* + *kdrl* (Bahary et al., 2007), and *jag1a* (Yamamoto et al., 2010) at the amounts specified. The MOs selected for this study were *tgfbR2*^{MO1}, *tgfb1a^{MO2}* + *tgfb1b^{MO2}* (referred to as *tgfb1*^{MO2}), *tgfb2^{MO2}*, and *tgfb3^{MO2}* at the amounts indicated (see Supplemental Experimental Procedures). Typically, 1 nl total volume of MO was injected in 1–4 cell stage embryos. MO design and validation is described in the Supplemental Experimental Procedures.

To rescue the loss of HSC markers in *tgfbR2* morphants, we transiently expressed *jag1a* in ECs under the control of the *Kdrl* promoter (Jin et al., 2005) (see Supplemental Experimental Procedures). The amount of DNA used for the rescue experiment is shown in the figure legends.

Western Blotting
Protein extracts were prepare as described (Link et al., 2006). TgfβR2 protein was detected by a primary anti-tgfβR2 antibody (diluted 1:250 in blocking solution, sc-17792; Santa Cruz Biotechnology) followed by a goat anti-mouse horseradish peroxidase (HRP)-conjugated secondary antibody (1:1,000 in blocking solution, P044701-2, DAKO). An anti β-actin-HRP-conjugated antibody (1:35,000, A3854; Sigma) was used for loading control.

NanoString Expression Analysis
To quantitate the effects of *tgfβR2* loss of function in and around the embryonic DA, trunks from anesthetized 26–28 hpf embryos were microdissected with a straight stab knife. Total RNA was isolated with the RNEasy Micro kit (QIAGEN) following the manufacturer's instructions and quantified in a Nanodrop spectrometer. We interrogated expression of a panel of 132 probes (see Supplemental Experimental Procedures) using the NanoString nCounter gene expression system.

mRNA Extraction, Flow Cytometry, cDNA Synthesis, and qPCR
Total RNA was isolated from wild-type or morpholino-injected embryos using TRI reagent (Sigma) and cleaned using the RNEasy Micro kit (QIAGEN) following the manufacturer's instructions. To interrogate gene expression in ECs of tgfβR2 morphants, uninjected and tgfβR2 MO1-injected Tg(kdrl:gfp) embryos were dissociated, and kdrl-GFP^{+} cells were isolated and processed for mRNA extraction with the RNEasy Micro kit (QIAGEN) as described (Monteiro et al., 2011). cDNA was synthesized from total RNA using a Superscript III RT-PCR enzyme (Invitrogen) following the manufacturer's instructions. The primers used for quantitative real-time PCR (qPCR) are shown in the Supplemental Experimental Procedures. Fold changes in gene expression were calculated using the $2^{-\Delta\Delta C_T}$ method (Livak and Schmittgen, 2001) and normalized to a geometric mean of *bactin2* and *ef1a*.

In Situ Hybridization, Sections, and Image Acquisition
Whole-mount in situ hybridization was carried out as described (Jowett and Yan, 1996). cDNA fragments for *tgfβR2*, *tgfβ1a*, *tgfβ1b*, and *tgfβ3* were PCR-amplified from 24 hpf embryo cDNA, cloned into pGEMT-Easy, and used as templates to generate in situ hybridization probes (see Supplemental Experimental Procedures). After in situ hybridization, embryos were processed and imaged as described (Gering and Patient, 2005; Monteiro et al., 2011).

Fluorescence Imaging and Image Processing
HSPCs express low levels of a GFP transgene under the control of the itga2b promoter (Kissa et al., 2008). Itga2b:GFP^{low}, kdrl:HsRas-mCherry^{+} HSPCs were imaged in uninjected and morpholino-injected Tg(itga2b:GFP; Kdrl:HsRas-mCherry) embryos at 48 hpf on a Zeiss LSM780 confocal microscope (Zen software). HSPCs were enumerated in maximum intensity projection images. GraphPad Prism software was used to generate scatterplots of cell counts and for statistical analysis. Alternatively, Tg(itga2b:GFP) embryos were imaged on a Zeiss Lumar V.12 stereomicroscope with an AxioCam MRm (Zeiss) and AxioVision software.

Apoptosis staining was performed with the Click-IT TUNEL Alexa 594 kit (C10246; Life Technologies) followed by immunostaining against GFP (see Supplemental Experimental Procedures).

Images were processed and figures and schemes were assembled in Adobe Photoshop CS5 and Adobe Illustrator CS5.

SUPPLEMENTAL INFORMATION

Supplemental Information includes Supplemental Experimental Procedures, six figures, and one table and can be found with this article online at http://dx.doi.org/10.1016/j.devcel.2016.06.024.

AUTHOR CONTRIBUTIONS

R.M. performed most experiments and analyzed the data; T.P., P.P., J.K., N.J., F.B., and A.K. performed experiments and analyzed the data; E.R. analyzed the NanoString data and performed statistical analysis; R.M. and R.P. conceived experiments, wrote the manuscript and secured funding.

ACKNOWLEDGMENTS

This research was funded by the British Heart Foundation (BHF Oxford CoRE Fellowship to R.M., BHF IBSR Fellowship FS/13/50/30436 to R.M., and a BHF Project Grant to J.K. and R.P. PG/14/39/30865), by the MRC (P.P., T.P., A.K., and R.P.), and by a Wolfson/Royal Society Merit Award (R.P.). We thank Maggie Walmsley for critical reading of the manuscript. We are very grateful to the staff of the Biomedical Services Unit for excellent fish husbandry. We thank Kevin Clark and Sally-Ann Clark for cell sorting. The flow cytometry facility is supported by the MRC HIU, MRC MHU (MC_UU_12009), NIHR Oxford BRC and John Fell Fund (131/030 and 101/517), the EPA fund (CF182 and CF170), and WIMM Strategic Alliance awards G0902418 and MC_UU_12025. We thank Christoffer Lagerholm for help with imaging. The Wolfson Imaging Centre Oxford is supported by the MRC via the WIMM Strategic Alliance (G0902418), the Molecular Haematology Unit (MC_UU_12009), the Human Immunology Unit (MC_UU_12010), the Wolfson Foundation (grant 18272), and an MRC/BBSRC/EPSRC grant (MR/K015777X/1) to MICA – Nanoscopy Oxford (NanO): Novel Super-resolution Imaging Applied to Biomedical Sciences, Micron (107457/Z/15Z). The facility was supported by WIMM Strategic Alliance awards G0902418 and MC_UU_12025.

Received: August 28, 2015
Revised: May 19, 2016
Accepted: June 21, 2016
Published: August 4, 2016

REFERENCES

Azzolin, L., Zanconato, F., Bresolin, S., Forcato, M., Basso, G., Bicciato, S., Cordenonsi, M., and Piccolo, S. (2012). Role of TAZ as mediator of Wnt signaling. Cell *151*, 1443–1456.

Bahary, N., Goishi, K., Stuckenholz, C., Weber, G., Leblanc, J., Schafer, C.A., Berman, S.S., Klagsbrun, M., and Zon, L.I. (2007). Duplicate VegfA genes and orthologues of the KDR receptor tyrosine kinase family mediate vascular development in the zebrafish. Blood *110*, 3627–3636.

Bertrand, J.Y., Chi, N.C., Santoso, B., Teng, S., Stainier, D.Y., and Traver, D. (2010). Haematopoietic stem cells derive directly from aortic endothelium during development. Nature *464*, 108–111.

Boisset, J.C., van Cappellen, W., Andrieu-Soler, C., Galjart, N., Dzierzak, E., and Robin, C. (2010). In vivo imaging of haematopoietic cells emerging from the mouse aortic endothelium. Nature *464*, 116–120.

Burns, C.E., Traver, D., Mayhall, E., Shepard, J.L., and Zon, L.I. (2005). Hematopoietic stem cell fate is established by the Notch-Runx pathway. Genes Dev. *19*, 2331–2342.

Butko, E., Distel, M., Pouget, C., Weijts, B., Kobayashi, I., Ng, K., Mosimann, C., Poulain, F.E., McPherson, A., Ni, C.W., et al. (2015). Gata2b is a restricted early regulator of hemogenic endothelium in the zebrafish embryo. Development *142*, 1050–1061.

Carvalho, R.L., Jonker, L., Goumans, M.J., Larsson, J., Bouwman, P., Karlsson, S., Dijke, P.T., Arthur, H.M., and Mummery, C.L. (2004). Defective paracrine signalling by TGFbeta in yolk sac vasculature of endoglin mutant mice: a paradigm for hereditary haemorrhagic telangiectasia. Development *131*, 6237–6247.

Challen, G.A., Boles, N.C., Chambers, S.M., and Goodell, M.A. (2010). Distinct hematopoietic stem cell subtypes are differentially regulated by TGF-beta1. Cell Stem Cell *6*, 265–278.

Cheah, F.S., Winkler, C., Jabs, E.W., and Chong, S.S. (2010). Tgfbeta3 regulation of chondrogenesis and osteogenesis in zebrafish is mediated through formation and survival of a subpopulation of the cranial neural crest. Mech. Dev. *127*, 329–344.

Chen, M.J., Yokomizo, T., Zeigler, B.M., Dzierzak, E., and Speck, N.A. (2009). Runx1 is required for the endothelial to haematopoietic cell transition but not thereafter. Nature *457*, 887–891.

Ciau-Uitz, A., Monteiro, R., Kirmizitas, A., and Patient, R. (2014). Developmental hematopoiesis: ontogeny, genetic programming and conservation. Exp. Hematol. *42*, 669–683.

Clements, W.K., Kim, A.D., Ong, K.G., Moore, J.C., Lawson, N.D., and Traver, D. (2011). A somitic Wnt16/Notch pathway specifies haematopoietic stem cells. Nature *474*, 220–224.

Cooney, J.D., Hildick-Smith, G.J., Shafizadeh, E., McBride, P.F., Carroll, K.J., Anderson, H., Shaw, G.C., Tamplin, O.J., Branco, D.S., Dalton, A.J., et al. (2013). Teleost growth factor independence (gfi) genes differentially regulate successive waves of hematopoiesis. Dev. Biol. *373*, 431–441.

de Bruijn, M.F., Ma, X., Robin, C., Ottersbach, K., Sanchez, M.J., and Dzierzak, E. (2002). Hematopoietic stem cells localize to the endothelial cell layer in the midgestation mouse aorta. Immunity *16*, 673–683.

Derynck, R., and Zhang, Y.E. (2003). Smad-dependent and Smad-independent pathways in TGF-beta family signalling. Nature *425*, 577–584.

Dickson, M.C., Martin, J.S., Cousins, F.M., Kulkarni, A.B., Karlsson, S., and Akhurst, R.J. (1995). Defective haematopoiesis and vasculogenesis in transforming growth factor-beta 1 knock out mice. Development *121*, 1845–1854.

Ditadi, A., Sturgeon, C.M., Tober, J., Awong, G., Kennedy, M., Yzaguirre, A.D., Azzola, L., Ng, E.S., Stanley, E.G., French, D.L., et al. (2015). Human definitive haemogenic endothelium and arterial vascular endothelium represent distinct lineages. Nat. Cell Biol. *17*, 580–591.

Espin-Palazon, R., Stachura, D.L., Campbell, C.A., Garcia-Moreno, D., Del Cid, N., Kim, A.D., Candel, S., Meseguer, J., Mulero, V., and Traver, D. (2014). Proinflammatory signaling regulates hematopoietic stem cell emergence. Cell *159*, 1070–1085.

Gama-Norton, L., Ferrando, E., Ruiz-Herguido, C., Liu, Z., Guiu, J., Islam, A.B., Lee, S.-U.U., Yan, M., Guidos, C.J., López-Bigas, N., et al. (2015). Notch signal strength controls cell fate in the haemogenic endothelium. Nat. Commun. *6*, 8510.

Geiss, G.K., Bumgarner, R.E., Birditt, B., Dahl, T., Dowidar, N., Dunaway, D.L., Fell, H.P., Ferree, S., George, R.D., Grogan, T., et al. (2008). Direct multiplexed measurement of gene expression with color-coded probe pairs. Nat. Biotechnol. *26*, 317–325.

Gering, M., and Patient, R. (2005). Hedgehog signaling is required for adult blood stem cell formation in zebrafish embryos. Dev. Cell *8*, 389–400.

Gering, M., and Patient, R. (2010). Notch signalling and haematopoietic stem cell formation during embryogenesis. J. Cell Physiol. *222*, 11–16.

Gore, A.V., Swift, M.R., Cha, Y.R., Lo, B., McKinney, M.C., Li, W., Castranova, D., Davis, A., Mukouyama, Y.S., and Weinstein, B.M. (2011). Rspo1/Wnt signaling promotes angiogenesis via Vegfc/Vegfr3. Development *138*, 4875–4886.

Goumans, M.J., and Mummery, C. (2000). Functional analysis of the TGFbeta receptor/Smad pathway through gene ablation in mice. Int. J. Dev. Biol. *44*, 253–265.

Goumans, M.J., Valdimarsdottir, G., Itoh, S., Rosendahl, A., Sideras, P., and ten Dijke, P. (2002). Balancing the activation state of the endothelium via two distinct TGF-beta type I receptors. EMBO J. *21*, 1743–1753.

Hadland, B.K., Varnum-Finney, B., Poulos, M.G., Moon, R.T., Butler, J.M., Rafii, S., and Bernstein, I.D. (2015). Endothelium and NOTCH specify and amplify aorta-gonad-mesonephros-derived hematopoietic stem cells. J. Clin. Invest. *125*, 2032–2045.

Hao, J., Ho, J.N., Lewis, J.A., Karim, K.A., Daniels, R.N., Gentry, P.R., Hopkins, C.R., Lindsley, C.W., and Hong, C.C. (2010). In vivo structure-activity relationship study of dorsomorphin analogues identifies selective VEGF and BMP inhibitors. ACS Chem. Biol. *5*, 245–253.

Holderfield, M.T., and Hughes, C.C. (2008). Crosstalk between vascular endothelial growth factor, notch, and transforming growth factor-beta in vascular morphogenesis. Circ. Res. *102*, 637–652.

Jang, I.H., Lu, Y.-F.F., Zhao, L., Wenzel, P.L., Kume, T., Datta, S.M., Arora, N., Guiu, J., Lagha, M., Kim, P.G., et al. (2015). Notch1 acts via Foxc2 to promote definitive hematopoiesis via effects on hemogenic endothelium. Blood *125*, 1418–1426.

Jin, S.W., Beis, D., Mitchell, T., Chen, J.N., and Stainier, D.Y. (2005). Cellular and molecular analyses of vascular tube and lumen formation in zebrafish. Development *132*, 5199–5209.

Jowett, T., and Yan, Y.L. (1996). Double fluorescent in situ hybridization to zebrafish embryos. Trends Genet. *12*, 387–389.

Kaartinen, V., Voncken, J.W., Shuler, C., Warburton, D., Bu, D., Heisterkamp, N., and Groffen, J. (1995). Abnormal lung development and cleft palate in mice lacking TGF-beta 3 indicates defects of epithelial-mesenchymal interaction. Nat. Genet. *11*, 415–421.

Kim, S.J., and Letterio, J. (2003). Transforming growth factor-beta signaling in normal and malignant hematopoiesis. Leukemia *17*, 1731–1737.

Kim, A.D., Melick, C.H., Clements, W.K., Stachura, D.L., Distel, M., Panakova, D., MacRae, C., Mork, L.A., Crump, J.G., and Traver, D. (2014). Discrete Notch signaling requirements in the specification of hematopoietic stem cells. EMBO J. *33*, 2363–2373.

Kissa, K., and Herbomel, P. (2010). Blood stem cells emerge from aortic endothelium by a novel type of cell transition. Nature *464*, 112–115.

Kissa, K., Murayama, E., Zapata, A., Cortes, A., Perret, E., Machu, C., and Herbomel, P. (2008). Live imaging of emerging hematopoietic stem cells and early thymus colonization. Blood *111*, 1147–1156.

Lam, E.Y., Hall, C.J., Crosier, P.S., Crosier, K.E., and Flores, M.V. (2010). Live imaging of Runx1 expression in the dorsal aorta tracks the emergence of blood progenitors from endothelial cells. Blood *116*, 909–914.

Lamouille, S., Xu, J., and Derynck, R. (2014). Molecular mechanisms of epithelial-mesenchymal transition. Nat. Rev. Mol. Cell Biol. *15*, 178–196.

Lawson, N.D., and Weinstein, B.M. (2002). In vivo imaging of embryonic vascular development using transgenic zebrafish. Dev. Biol. *248*, 307–318.

Lawson, N.D., Vogel, A.M., and Weinstein, B.M. (2002). Sonic hedgehog and vascular endothelial growth factor act upstream of the notch pathway during arterial endothelial differentiation. Dev. Cell *3*, 127–136.

Leung, A., Ciau-Uitz, A., Pinheiro, P., Monteiro, R., Zuo, J., Vyas, P., Patient, R., and Porcher, C. (2013). Uncoupling VEGFA functions in arteriogenesis and hematopoietic stem cell specification. Dev. Cell *24*, 144–158.

Lin, H.F., Traver, D., Zhu, H., Dooley, K., Paw, B.H., Zon, L.I., and Handin, R.I. (2005). Analysis of thrombocyte development in CD41-GFP transgenic zebrafish. Blood *106*, 3803–3810.

Link, V., Shevchenko, A., and Heisenberg, C.P. (2006). Proteomics of early zebrafish embryos. BMC Dev. Biol. *6*, 1.

CellPress

Livak, K.J., and Schmittgen, T.D. (2001). Analysis of relative gene expression data using real-time quantitative PCR and the 2(-Delta Delta C(T)) Method. Methods *25*, 402–408.

Massague, J., and Gomis, R.R. (2006). The logic of TGFbeta signaling. FEBS Lett. *580*, 2811–2820.

Menendez, D., Inga, A., and Resnick, M.A. (2009). The expanding universe of p53 targets. Nat. Rev. Cancer *9*, 724–737.

Monteiro, R., Pouget, C., and Patient, R. (2011). The gata1/pu.1 lineage fate paradigm varies between blood populations and is modulated by tif1gamma. EMBO J. *30*, 1093–1103.

Murayama, E., Kissa, K., Zapata, A., Mordelet, E., Briolat, V., Lin, H.F., Handin, R.I., and Herbomel, P. (2006). Tracing hematopoietic precursor migration to successive hematopoietic organs during zebrafish development. Immunity *25*, 963–975.

Nawshad, A., Medici, D., Liu, C.C., and Hay, E.D. (2007). TGFbeta3 inhibits E-cadherin gene expression in palate medial-edge epithelial cells through a Smad2-Smad4-LEF1 transcription complex. J. Cell Sci. *120*, 1646–1653.

Nimmo, R., Ciau-Uitz, A., Ruiz-Herguido, C., Soneji, S., Bigas, A., Patient, R., and Enver, T. (2013). MiR-142-3p controls the specification of definitive hemangioblasts during ontogeny. Dev. Cell *26*, 237–249.

North, T.E., Goessling, W., Walkley, C.R., Lengerke, C., Kopani, K.R., Lord, A.M., Weber, G.J., Bowman, T.V., Jang, I.H., Grosser, T., et al. (2007). Prostaglandin E2 regulates vertebrate haematopoietic stem cell homeostasis. Nature *447*, 1007–1011.

Oh, S.P., Seki, T., Goss, K.A., Imamura, T., Yi, Y., Donahoe, P.K., Li, L., Miyazono, K., ten Dijke, P., Kim, S., et al. (2000). Activin receptor-like kinase 1 modulates transforming growth factor-beta 1 signaling in the regulation of angiogenesis. Proc. Natl. Acad. Sci. USA *97*, 2626–2631.

Oshima, M., Oshima, H., and Taketo, M.M. (1996). TGF-beta receptor type II deficiency results in defects of yolk sac hematopoiesis and vasculogenesis. Dev. Biol. *179*, 297–302.

Pardali, E., Goumans, M.-J.J., and ten Dijke, P. (2010). Signaling by members of the TGF-beta family in vascular morphogenesis and disease. Trends Cell Biol. *20*, 556–567.

Proetzel, G., Pawlowski, S.A., Wiles, M.V., Yin, M., Boivin, G.P., Howles, P.N., Ding, J., Ferguson, M.W., and Doetschman, T. (1995). Transforming growth factor-beta 3 is required for secondary palate fusion. Nat. Genet. *11*, 409–414.

Quillien, A., Moore, J.C., Shin, M., Siekmann, A.F., Smith, T., Pan, L., Moens, C.B., Parsons, M.J., and Lawson, N.D. (2014). Distinct Notch signaling outputs pattern the developing arterial system. Development *141*, 1544–1552.

Robert-Moreno, A., Espinosa, L., de la Pompa, J.L., and Bigas, A. (2005). RBPjkappa-dependent Notch function regulates Gata2 and is essential for the formation of intra-embryonic hematopoietic cells. Development *132*, 1117–1126.

Robert-Moreno, A., Guiu, J., Ruiz-Herguido, C., Lopez, M.E., Ingles-Esteve, J., Riera, L., Tipping, A., Enver, T., Dzierzak, E., Gridley, T., et al. (2008). Impaired embryonic haematopoiesis yet normal arterial development in the absence of the Notch ligand Jagged1. EMBO J. *27*, 1886–1895.

Robu, M.E., Larson, J.D., Nasevicius, A., Beiraghi, S., Brenner, C., Farber, S.A., and Ekker, S.C. (2007). p53 activation by knockdown technologies. PLoS Genet. *3*, e78.

Rowlinson, J.M., and Gering, M. (2010). Hey2 acts upstream of Notch in hematopoietic stem cell specification in zebrafish embryos. Blood *116*, 2046–2056.

Santoro, M., Pesce, G., and Stainier, D. (2009). Characterization of vascular mural cells during zebrafish development. Mech. Dev. *126*, 638–649.

Shi, Y., and Massague, J. (2003). Mechanisms of TGF-beta signaling from cell membrane to the nucleus. Cell *113*, 685–700.

Vargel, Ö., Zhang, Y., Kosim, K., Ganter, K., Foehr, S., Mardenborough, Y., Shvartsman, M., Enright, A.J., Krijgsveld, J., and Lancrin, C. (2016). Activation of the TGFβ pathway impairs endothelial to haematopoietic transition. Sci. Rep. *6*, 21518.

Walsh, D.M., Klyubin, I., Fadeeva, J.V., Cullen, W.K., Anwyl, R., Wolfe, M.S., Rowan, M.J., and Selkoe, D.J. (2002). Naturally secreted oligomers of amyloid beta protein potently inhibit hippocampal long-term potentiation in vivo. Nature *416*, 535–539.

Westerfield, M. (2000). The Zebrafish Book. A Guide for the Laboratory Use of Zebrafish (Danio rerio) (Eugene: University of Oregon Press).

Wilkinson, R.N., Pouget, C., Gering, M., Russell, A.J., Davies, S.G., Kimelman, D., and Patient, R. (2009). Hedgehog and Bmp polarize hematopoietic stem cell emergence in the zebrafish dorsal aorta. Dev. Cell *16*, 909–916.

Yamamoto, M., Morita, R., Mizoguchi, T., Matsuo, H., Isoda, M., Ishitani, T., Chitnis, A.B., Matsumoto, K., Crump, J.G., Hozumi, K., et al. (2010). Mib-Jag1-Notch signalling regulates patterning and structural roles of the notochord by controlling cell-fate decisions. Development *137*, 2527–2537.

Zavadil, J., Cermak, L., Soto-Nieves, N., and Bottinger, E.P. (2004). Integration of TGF-beta/Smad and Jagged1/Notch signalling in epithelial-to-mesenchymal transition. EMBO J. *23*, 1155–1165.

Zhao, M., Perry, J.M., Marshall, H., Venkatraman, A., Qian, P., He, X.C., Ahamed, J., and Li, L. (2014). Megakaryocytes maintain homeostatic quiescence and promote post-injury regeneration of hematopoietic stem cells. Nat. Med. *20*, 1321–1326.

Developmental Cell, Volume *38*

Supplemental Information

Transforming Growth Factor β Drives Hemogenic Endothelium Programming and the Transition to Hematopoietic Stem Cells

Rui Monteiro, Philip Pinheiro, Nicola Joseph, Tessa Peterkin, Jana Koth, Emmanouela Repapi, Florian Bonkhofer, Arif Kirmizitas, and Roger Patient

Supplemental Information Inventory

Supplemental Figures S1-S6

Figure S1, related to Figure 1

Figure S2, related to Figure 2

Figure S3, related to Figure 3

Figure S4, related to Figure 4

Figure S5, related to Figure 5

Figure S6, related to Figure 6

Supplemental Table S1 (excel file), related to Figure 5

Supplemental Experimental Procedures, related to Figures 1-6, S1-S6

Supplemental References, related to Supplemental Experimental Procedures and to Supplemental Table S1

Supplemental Figures and Figure Legends

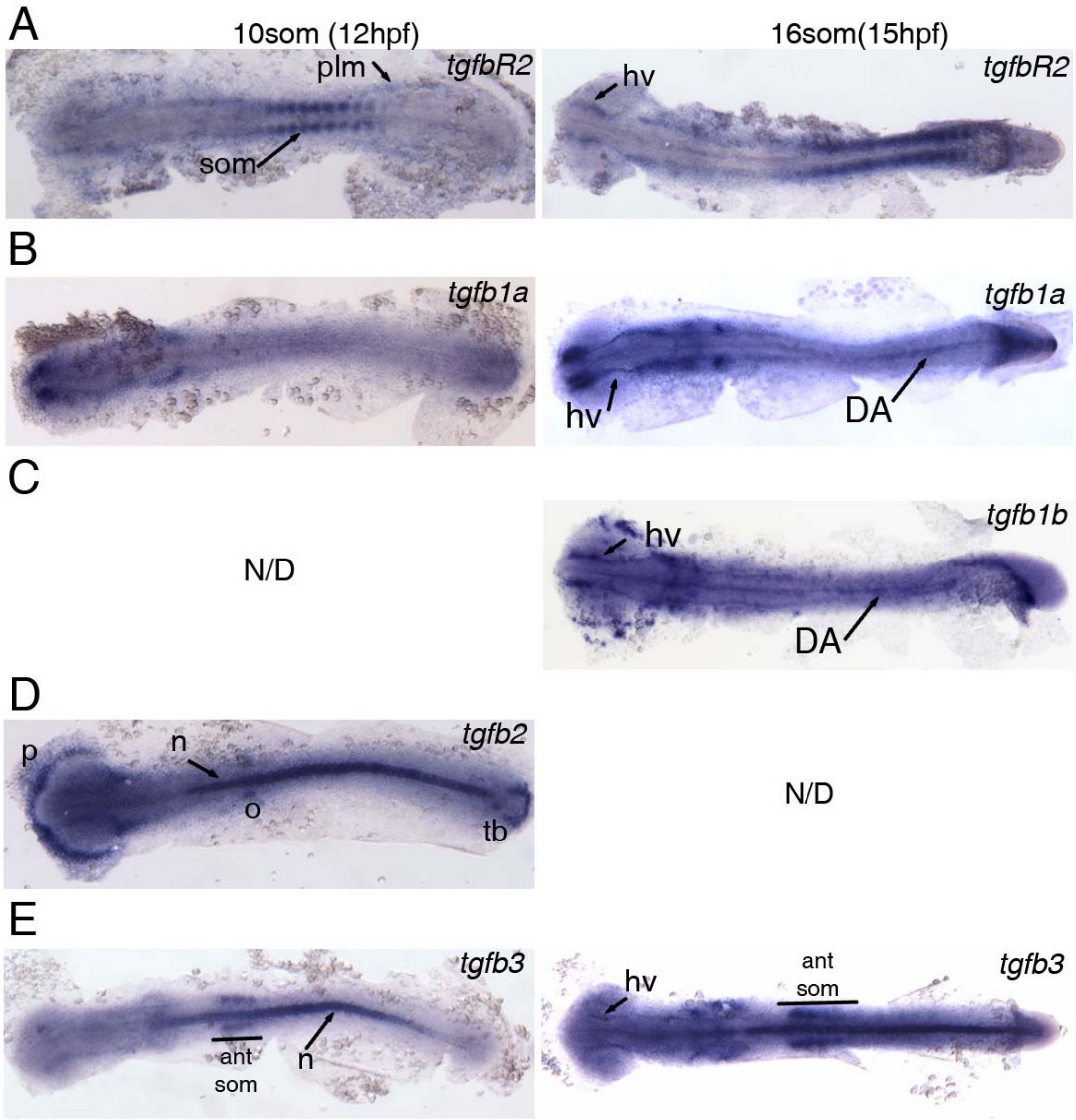

Figure S1, related to Figure 1 – Expression of TGFβ signalling components at 12hpf (10-somite stage) and 15hpf (16-somite stage). All embryos were flatmounted for imaging. (A) Expression of *tgfbR2* in the somites (som) and posterior lateral mesoderm (plm) at 12hpf. Expression of *tgfbR2* at 15hpf in head vasculature (hv) and somites. (B) *tgfb1a* is not expressed in the posterior at 12hpf but is present in head vasculature from 12hpf and in the embryonic dorsal aorta (DA) at 15hpf. (C) *tgfb1b* is also present in the head vasculture and DA at 15hpf. Note that there is anterior expression of *tgfb1b* in cells that are likely myeloid. (D) *tgfb2* is expressed in the polster (p), otic vesicles (o), notochord (n) and in the tailbud (tb) at 12hpf. (E) Expression of *tgfb3* in the notochord and in the 4 anterior-most somites (ant som) at 12hpf. At 15hpf, the expression in the somites and notchord is maintained and weak expression in the head vasculature is observed.

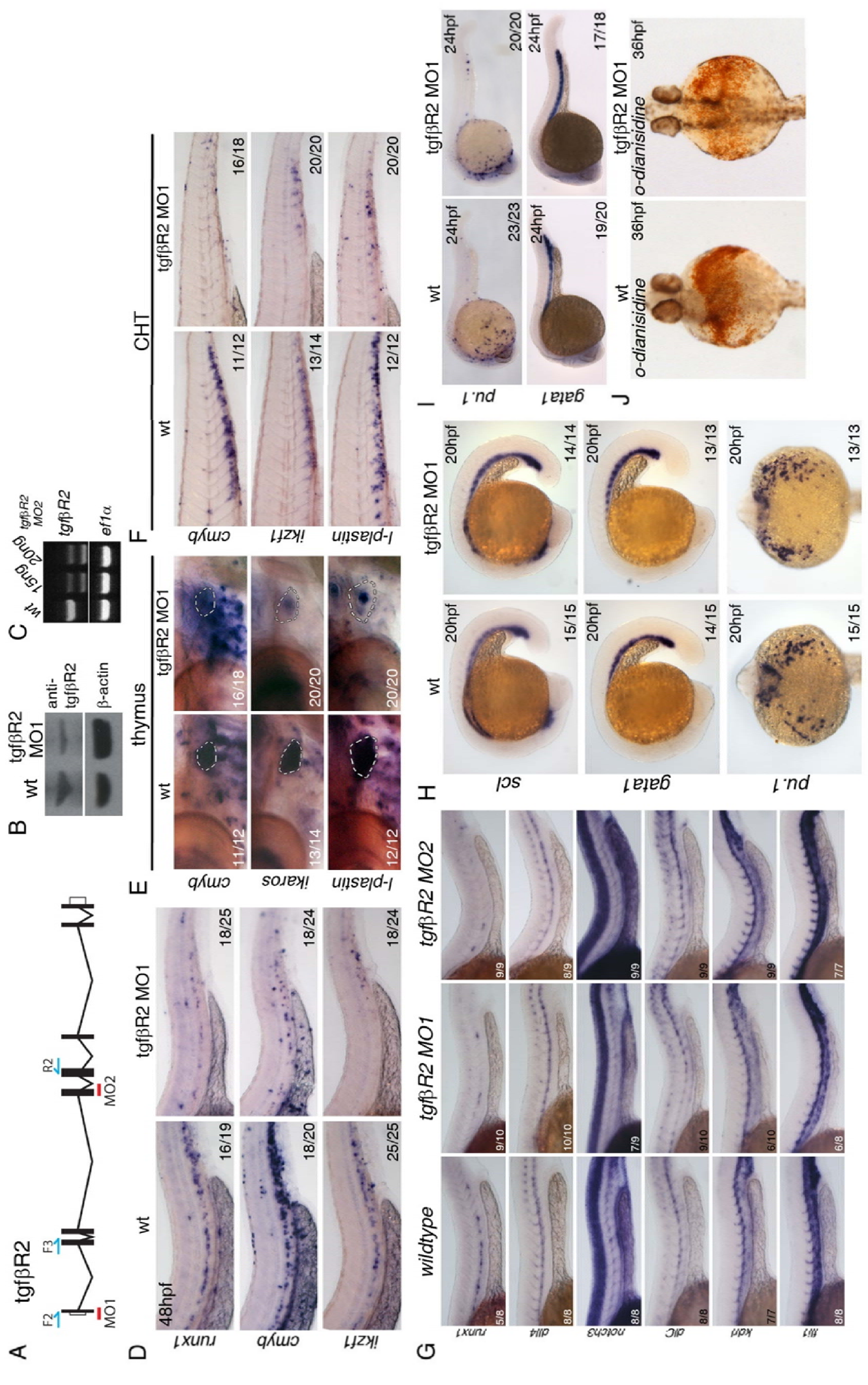

Figure S2, related to Figure 2 – Further characterization and validation of two independent morpholinos targeting tgfβR2 and analysis of primitive haematopoiesis in tgfβR2 morphants. (A) Schematic representation of the genomic organization of the tgfbR2 gene, location of morpholinos

and primers used to validate the morpholinos. (B) Western blot against TgfβR2 showing a decrease of WT protein induced by the *tgfbR2*^{MO1} morpholino. (C) Validation of the *tgfbR2*^{MO2} by qPCR on 24hpf cDNA with tgfbR2 F2/R2 primers (see below). Ef1a PCR was used as a control for the PCR. (D) Expression of *runx1*, *cmyb* and *ikzf1* at 48hpf is reduced in the trunk and CHT of *tgfbR2* morphants. Expression of *cmyb*, *ikzf1* and *l-plastin* is severely reduced (E) in the thymus and (F) in the CHT of *tgfbR2* morphants at 4dpf. (G) Characterisation of the tgfbR2 MO1 and MO2 morpholinos. *Runx1* expression is decreased upon injection of either morpholino, whereas the arterial markers dll4, notch3 and dlC and the vascular markers kdrl and fli1 are grossly normal. (H) Expression of *scl*, *gata1* and *pu.1* is indistinguishable between wildtype and *tgfbR2* morphant embryos at 20hpf. (I) Expression of the primitive hematopoietic markers *gata1* and *pu.1* is unaffected in *tgfbR2* morphants at 24hpf. (J) *tgfbR2* morphants show a slight reduction in o-dianisidine staining at 36hpf. Numbers of embryos analysed are shown in each panel as number of affected embryos/total observed.

Figure S3

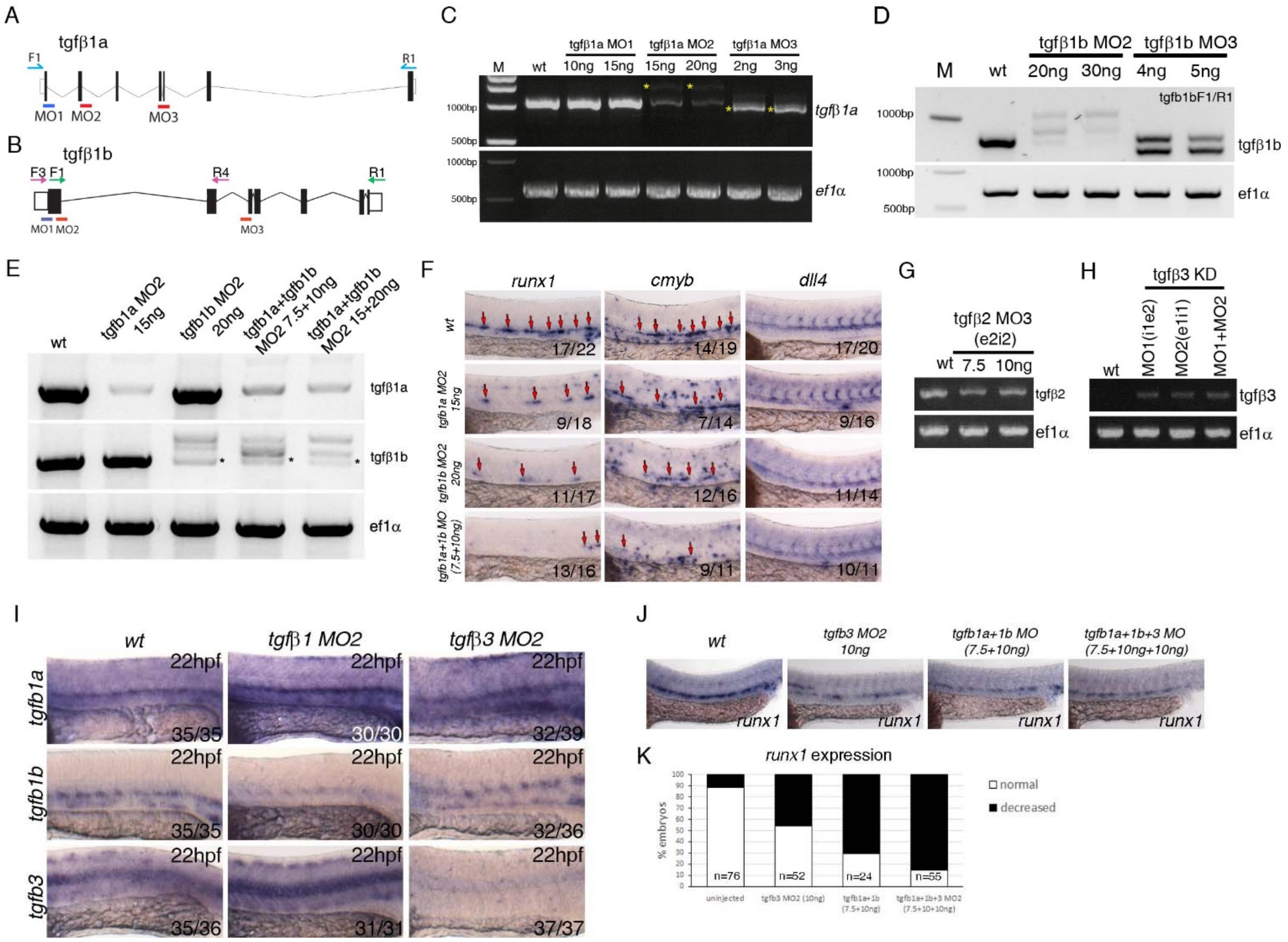

Figure S3, related to Figure 3 – Validation of morpholinos targeting *tgfβ1a*, *tgfβ1b*, *tgfβ2* and *tgfβ3* and further characterization of the phenotype. (A) Schematic representation of the genomic organization of the *tgfb1a* gene, location of morpholinos and primers used to validate morpholino activity. (B) Schematic representation of the genomic organization of the *tgfb1b* gene, location of morpholinos and primers used to validate morpholino activity. (C) Validation of the activity of the *tgfb1a* morpholinos by PCR. Yellow asterisk marks PCR products generated as a result of aberrant splicing induced by the *tgfb1a* morpholinos MO2 and MO3. We have used *tgfb1a*^{MO2} in all of the subsequent analysis. (D) Validation by PCR of the activity of two antisense morpholinos targeting *tgfb1b*. Clear aberrant splicing was induced by *tgfb1b* morpholinos MO2 and MO3. We have used *tgfb1b*^{MO2} in all of the subsequent analysis. (E) Validation by PCR of the *tgfb1a*^{MO2}+*tgfb1b*^{MO2} combination. Asterisk marks the remainder of the normally spliced *tgfb1b* gene product. (F) Testing the *tgfb1a*+*tgfb1b* MO2 combinations by *in situ* hybridisation against HE markers *runx1*, HSPC marker *cmyb* and arterial marker *dll4* at 28hpf. The combination of *tgfb1a*+*tgfb1b* MO2 (referred to

in the main text as *tgfb1*^{MO}) was used at 7.5 ng/μl +10ng/μl throughout the manuscript. Numbers of embryos analysed are shown in each panel as number of affected embryos/total observed. (G) Validation by PCR of the activity of a morpholino targeting *tgfb2*. (H) Validation by PCR of the activity of two published morpholinos (Cheah et al, 2010) targeting *tgfb3*. *Tgfb3*^{MO2} was used in all of the subsequent analysis. (I) Expression of *tgfb1a*, *tgfb1b* and *tgfb3* in *tgfb1*^{MO2} and *tgfb3*^{MO2} at 22hpf. *tgfb1*^{MO2} morphants show an increase in *tgfb1a* and *tgfb3* and a decrease in *tgfb1b*; *tgfb3*^{MO2} morphants show a dramataic loss of *tgfb3* expression. Numbers of embryos analysed are shown in each panel as number of affected embryos/total observed. (J,K) Comparison and quantification of the runx1 expression in the DA upon single tgfb3^{MO2}, tgfb1^{MO2} or combined tgfb3^{MO2}+tgfb1^{MO2}, showing increased severity of the phenotype in the triple morphants as compared to single morphants. The numbers of embryos analysed are shown in the graph in (K).

Figure S4

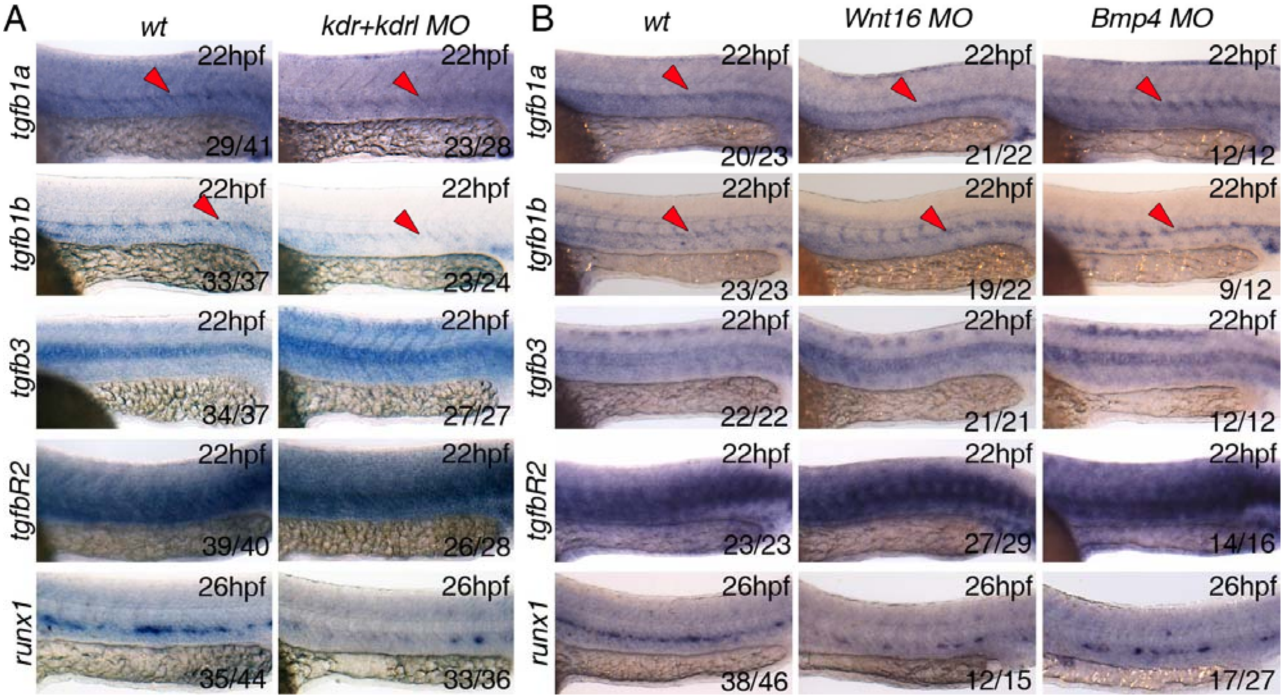

Figure S4, related to Figure 4 – Inhibition of Vegf (*kdr+kdrl*^{MO}), Wnt (*Wnt16*^{MO}) or BMP (*BMP4*^{MO}) signalling pathways by morpholino oligonucleotides. All *in situ* hybridisation experiments were performed at 22hpf except for the *runx1* probe, performed at 26hpf. (A) Loss of Vegf signalling upon morpholino knockdown of the Vegf receptors *kdrl* and *kdrl* (Bahary et al, 2007) results in decreased expression of *tgfb1a* and *tgfb1b* in the dorsal aorta (DA). *Runx1* expression in the DA was used as a positive control for the experiment. (B) Loss of Wnt signalling or BMP signalling by morpholino knockdown of Wnt16 (Clemens et al, 2011) or BMP4 (Chocron et al, 2007) showed no effect of expression of TGFβ ligands or receptor. *Runx1* expression in the DA was used as a positive control for the experiment. Numbers of embryos analysed are shown in each panel as number of affected embryos/total observed.

Figure S5

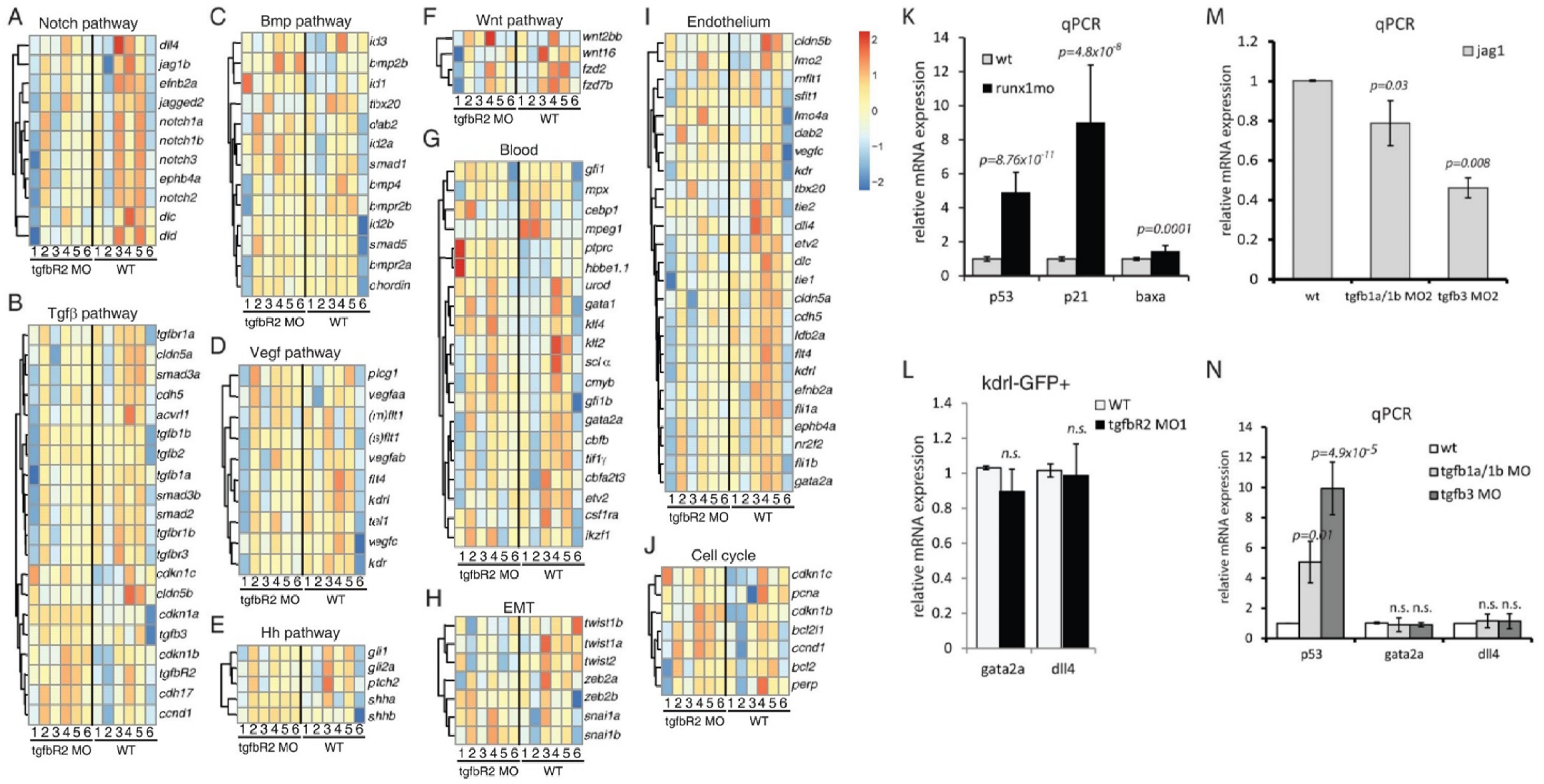

A
Notch pathway
dll4
jag1b
efnb2a
jagged2
notch1a
notch1b
notch3
ephb4a
notch2
dlc
dld
tgfbR2 MO
WT
B
Tgfβ pathway
tgfbr1a
cldn5a
smad3a
cdh5
acvrl1
tgfb1b
tgfb2
tgfb1a
smad3b
smad2
tgfbr1b
tgfbr3
cdkn1c
cldn5b
cdkn1a
tgfb3
cdkn1b
tgfbR2
cdh17
ccnd1
C
Bmp pathway
id3
bmp2b
id1
tbx20
dab2
id2a
smad1
bmp4
bmpr2b
id2b
smad5
bmpr2a
chordin
D
Vegf pathway
plcg1
vegfaa
(m)flt1
(s)flt1
vegfab
flt4
kdrl
tel1
vegfc
kdr
E
Hh pathway
gli1
gli2a
ptch2
shha
shhb
F
Wnt pathway
wnt2bb
wnt16
fzd2
fzd7b
G
Blood
gfi1
mpx
cebp1
mpeg1
ptprc
hbbe1.1
urod
gata1
klf4
klf2
scl α
cmyb
gfi1b
gata2a
cbfb
tif1γ
cbfa2t3
etv2
csf1ra
ikzf1
H
EMT
twist1b
twist1a
twist2
zeb2a
zeb2b
snai1a
snai1b
I
Endothelium
cldn5b
lmo2
mflt1
sflt1
lmo4a
dab2
vegfc
kdr
tbx20
tie2
dll4
etv2
dlc
tie1
cldn5a
cdh5
ldb2a
flt4
kdrl
efnb2a
fli1a
ephb4a
nr2f2
fli1b
gata2a
J
Cell cycle
cdkn1c
pcna
cdkn1b
bcl2l1
ccnd1
bcl2
perp
K
qPCR
wt
runx1mo
relative mRNA expression
p=8.76x10^-11
p=4.8x10^-8
p=0.0001
p53
p21
baxa
L
kdrl-GFP+
WT
tgfbR2 MO1
n.s.
gata2a
dll4
M
qPCR
jag1
p=0.03
p=0.008
wt
tgfb1a/1b MO2
tgfb3 MO2
N
qPCR
wt
tgfb1a/1b MO
tgfb3 MO
p=4.9x10^-5
p=0.01
n.s. n.s.
p53
gata2a
dll4

Figure S5, related to Figure 1 and Table S1 – Multiplex analysis of the gene expression profile in *tgfbR2* morphant embryos of a custom Probe Set (132 genes) using Nanostring (see Supplementary Experimental Procedures and Table S1). The custom designed probes include genes that belong to known pathway or processes that affect endothelial or hematopoietic cell programming, fate or survival, as well as six housekeeping genes. All genes shown here displayed no significant changes in expression upon $tgfbR2^{MO1}$ knockdown (see Figure 5 for differentially expressed genes). Each column represents one sample in a total of six wt and six $tgfbR2^{MO1}$ replicate morpholino injections (A) Notch pathway genes. (B) TGFβ pathway. (C) BMP pathway. (D) Vegf pathway. (E) Hedgehog pathway. (F) Wnt pathway. (G) Blood: genes expressed mostly in HSPCs, erythroid or myeloid cells. (H) Genes with known roles in epithelial to mesenchymal transition (EMT). (I) Gene expressed in endothelial cells (J) Cell cycle: genes that mark proliferation or apoptosis. Note that expression data is repeated for some genes as they fall into more than one category. (K) *p53*, *cdkn1a* and *baxa* are upregulated in *runx1* morphants. Trunks from 20 wild type and 20 *runx1* morphant embryos at 27hpf were dissected and the cDNA analysed by qPCR. The data represents the average of 4 biological replicates and were normalised to *gapdh*. (L) qPCR in kdrl-GFP^{+} endothelial cells confirms that neither *gata2a* nor *dll4* expression were affected by *tgfbR2* morpholino knockdown at 26hpf. (M) qPCR for *jag1a* in *tgfb1* and *tgfb3* morphants at 28hpf. (N) *p53* but not *gata2a* or *dll4* expression are affected in *tgfb1* (*tgfb1a* MO2+ *tgfb1b* MO2) or *tgfb3* morphants. qPCR results are shown as the average (±s.d.) of 3-6 biological replicates. Expression levels were normalized to *bactin2* and *ef1a*. *p* values are shown on the graphs. n.s.- not significant.

Figure S6

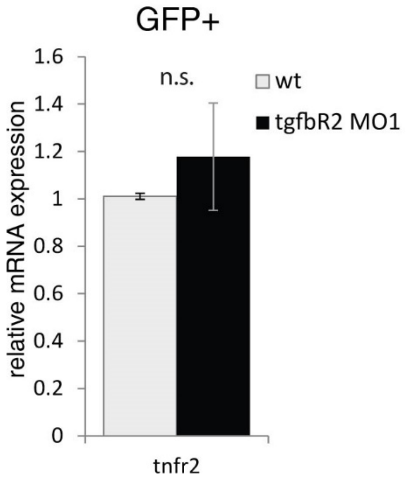

Figure S6, related to Figure 6 – qPCR analysis of *tnfr2* expression**.** *tnfr2* expression is unaffected in kdrl-GFP$^+$ endothelial cells at 26hpf in *tgfbR2* morphants. qPCR results are shown as the average (±s.d.) of 3 biological replicates; each biological replicate was done in triplicate. Expression levels were normalized to *bactin2* and *ef1a*. n.s.- not significant.

Supplemental Table S1 (excel file), related to Figure 5 - Mean Expression Values of the Data Analysed with the NanoStringNorm Package Sorted by Increasing P Value. The Top 9 Genes ($P<0.05$ and absolute log2FC> 0.5) are Highlighted. Eight of the Probes Failed and were not Considered in the Analysis.

Supplemental Experimental Procedures

Morpholinos, RNA and DNA injections and chemical inhibitors

To assess the role of TGFβ signalling in definitive haematopoiesis, we designed MOs targeting either the 5'-end or a splice site of *tgfβR2*, *tgfβ1a*, *tgfβ1b* and *tgfβ2* (see below; Fig. S2,S3). For splice blocking MOs, correct targeting was validated by PCR on cDNA from morphant embryos (Fig. S2, S3) using the primers indicated (see below). We validated the phenotypes induced by translation blocking MOs by comparing them against those induced by splice MOs for *tgfβR2*, *tgfβ1a*, *tgfβ1b* (Fig. S2,S3, data not shown). For *tgfβ2*, both MOs yielded the same phenotype (data not shown) but only tgfb2^{MO3} could be validated by PCR (Fig. S3G) so all experiments were done with tgfb2^{MO3}. For tgfbR2^{MO1}, the phenotype was further verified by western blot against TgfβR2 (Fig. S2B).

To investigate whether the Notch or Vegf pathways regulated the transcription of TGFβ ligands, we treated wildtype embryos with DMSO (control), the γ-secretase inhibitor inhibitor DAPM (565777, Calbiochem) or the Vegf inhibitor DMH4 (D8696, SIGMA) from tailbud stage until collection at 22hpf or 28hpf for analysis by in situ hybridization, at concentrations specified in the figures. To interrogate *kdrl*, *tgfb1a* and *tgfb1b* gene expression in endothelial cells of DMH4-treated embryos, treated and untreated Tg(Fli1a:gfp) embryos were dissociated and GFP$^+$ cells isolated and processed for mRNA extraction with the RNEasy Micro kit (Qiagen) as described (Monteiro et al., 2011). cDNA was synthesized from total RNA using a Superscript III RT-PCR enzyme (Invitrogen) following the manufacturer's instructions. The primers used for quantitative real-time PCR (qPCR) are shown in below. Fold changes in gene expression were calculated using the $2^{-\Delta\Delta C_T}$ method (Livak and Schmittgen, 2001) and normalized to a geometric mean of *bactin2* and *ef1a*.

To rescue the loss of HSC markers in *tgfbR2* morphants we transiently expressed *jag1a* (Ensembl ID: ENSDARG00000030289) in endothelial cells under the control of the *Kdrl* promoter (Jin et al., 2005). The jag1a sequence was PCR-amplified from 24hpf embryo cDNA and a C-terminal V5 tag was added

in frame in the 3’end primer (see below for primers). This amplicon was then cloned into a Tol2 destination vector, downstream of the Kdrl promoter using the InFusion HD Cloning kit (Takara Clontech). The resulting Kdrl:jag1a-V5 construct was confirmed by sequencing. Additional details available upon request. The amount of DNA used for the rescue experiment is shown in the figure legends.

Western blotting

Protein extracts were prepare as described (Link et al., 2006); samples were sonicated in a Bioruptor sonicator (Diagenode) prior to loading on gel. After transfer, membranes were blocked in 5% milk (SIGMA) in Tris-Buffered Saline+0.5%Tween-20 (TBST) for 1h at RT. TgfbR2 protein was detected by a primary anti-tgfbR2 antibody (diluted 1:250 in blocking solution, sc-17792, Santa Cruz). A goat anti-mouse HRP-conjugated was used as a secondary antibody (1:1000 in blocking solution, P044701-2, DAKO) and developed with ImmunoCruz luminol reagent (SantaCruz). Blots were stripped with Invitrogen’s stripping buffer, blocked again in 5% milk in TBST and re-probed with an anti β-actin-HRP conjugated antibody (1:35000, A3854, SIGMA).

NanoString expression analysis

This technology is as sensitive as qPCR but gene expression levels are obtained by counting mRNA molecules that hybridise with specifically tagged probes and does not require amplification (Geiss et al., 2008). We used 100ng of total RNA to hybridise with the capture and reporter probes overnight at 65°C. After the washes, the Target/Probe complexes were eluted and immobilized in the cartridge for data collection in the nCounter Digital Analyzer according to manufacturer’s instructions. The raw data was analysed using the NanoStringNorm R package (Waggott et al., 2012). The data was normalised using the geometric mean of the six positive controls and then it was background corrected by subtracting the mean and 2 standard deviations of the eight negative controls. The data was then

normalised for sample/RNA content using the geometric mean of five housekeeping genes (*bactin1*, *bactin2*, *gapdhs*, *sdha* and *ubiC*). Normalised mRNA expression levels were log2 transformed and analysed using a t-test to identify differentially expressed genes between conditions. The pheatmap package (Kolde, 2013) was used to generate heatmaps. The values used were the scaled per gene normalised values from the NanoStringNorm package (Waggott et al., 2012).

Immunohistochemistry and apoptosis staining

To evaluate apoptosis in *tgfbR2* morphants, we stained for apoptotic cells using the Click-IT TUNEL® Alexa 594 kit (C10246, LifeTechnologies) followed by immunostaining against GFP. Briefly, embryos at the desired stage were fixed for 1h at RT in 4% paraformaldehyde in PBS (PFA), permeabilized in PBS+0.25% TritonX-100 for 20min at RT and then washed twice in deionized water. The TdT and Click-IT reactions were performed according to the manufacturer's instructions. For detection of GFP following the Click-IT reaction, samples were blocked in 3%BSA, 5% goat serum in PBS+0.5% TritonX-100 (PBST) for 1h at RT and incubated overnight at 4°C with a chicken anti-GFP antibody in blocking solution (1:500, ab13970, Abcam). Samples were washed 6x15min in PBST, blocked again in 3%BSA, 5% goat serum in PBST for 1h at RT and incubated overnight at 4°C with a goat anti-chicken Alexa 488 conjugated antibody (1:500, A-11039, Invitrogen). Following 6x15min washes in PBST, the embryos were mounted in Vectashield® and imaged in an LSM780 confocal microscope with a LD C-Apochromat 40x/1.1 W objective.

Morpholinos, primers and NanoString probes

Antisense Morpholino Oligonucleotides (MOs) Used in this Study.

Gene	Ensembl ID	MO name	MO type	MO sequence (5'->3')	amount injected	published	comments
**tgfbR2**	ENSDART00000039832	tgfbr2 MO1	ATG	ATATCGCTCCATTAGAAACGCAGTC	12.5ng	this study	
		tgfbr2 MO2	e4i4	ATATTAAGTTGTCTCCTGACCTGCA	10ng	this study	
**tgfb1a**	ENSDART00000060839	tgfb1a MO1	ATG	CAGCACCAAGCAAACCAACCTCATA	10ng	this study	did not work
		tgfb1a MO2	e2i2	TGGTGCAACAATCACCTCACCTGAA	15ng	this study	
		tgfb1a MO3	e4i4	GGACAGCAAAAAGACTTACTCATCA	2ng	this study	
**tgfb1b**	ENSDART00000028981	tgfb1b MO1	ATG	GTAATAAACTCTCCGCCTTCATGGT	1ng	this study	
		tgfb1b MO2	e1i1	AAGGATAGTGCCACTCACTCATTGT	20ng	this study	
**tgfb2**	ENSDARG00000027087	tgfb2 MO1	ATG	GGAGGCTCAAGACGTACAAGTTCAT	5ng	this study	
		tgfb2 MO3	e2i2	AAAGGGACTTTGGATTTACCTGGTA	7.5ng	this study	
**tgfb3**	ENSDART00000019766	tgfb3 MO2	splice	CATCATCCCTAAGGGAAACTTACTG	17ng	(Cheah et al., 2010)	
**kdrl**		Kdrl MO		CCGAATGATACTCCGTATGTCACTT	4.5ng (in combination with kdr MO)	or MO2 kdrl (Bahary et al., 2007)	
**kdr**		Kdr MO		GTTTTCTTGATCTCACCTGAACCCT	4.5ng (in combination with kdrl MO)	or MO1 Kdrb (Bahary et al., 2007)	
**bmp4**		bmp4 MO	splice	GGTGTTTGATTGTCTGACCTTCATG	2ng	(Chocron et al., 2007)	
**wnt16**		wnt16 MO		AGGTTAGTTCTGTCACCCACCTGTC	5ng	(Clements et al., 2011)	
**runx1**		runx1 MO	splice	AGCGCTCTTACCGTATTTGGCGTCC	5ng	(Gering and Patient, 2005)	
**jag1a**		jag1a MO	splice	AAGCCAAACCCGCACATACCCGCAT	6ng	(Yamamoto et al., 2010)	

Primers Used in this Study.

Gene	Acession number /Ensembl ID	primer name	primer sequence	purpose	anti-sense linearisation	in vitro trans-cription
**tgfb1a**	Acession:NM_182873	tgfb1a F1	TCAGACGCTTTTCGATCCTT	generate in situ probe; test splice MO	ApaI	Sp6
		tgfb1a R1	AGGACCCCATGCAGTAGTTG			
**tgfb1b**	Ensembl ID:ENSDARG00000034895 (ENSDART00000028981)	tgfb1b F1	GCACACCATAGAAGATCCAACA	generate in situ probe; test splice MO	ApaI	Sp6
		tgfb1b R1	TGACAACTGTTCCACCTTATGC			
**tgfb2**	Accession:NM_194385	tgfb2 F1	GTTCAAGAAGAAGCGGATCG	generate in situ probe	SpeI	T7
		tgfb2 R1	GGGGTCTTGCCGATGTAGTA			
	Ensembl ID:ENSDART00000030271	tgfb2 F2	GTTCAAGAAGAAGCGGATCG	verify splice MO		
		tgfb2 R2	TGTCTGCGCTCCACAGATAC			
**tgfb3**	Accession:NM_194386	tgfb3 F1	TGGCTGACAAACAGAGCAAC	generate in situ probe	ApaI	Sp6
		tgfb3 R1	CTGCCGTGTGACAGAGGTAA			
	Cheah et al, 2010	tgfb3 F2	TCACACTTAGTTCATGTTAG	to verify splice MO (362bp in MO, but not in WT)		
		tgfb3 R2	TGTAGCGCTGCTTGCCGA			
	Cheah et al, 2010	tgfb3 F4	CGGGCAGGACAACACTGA	qPCR		
		tgfb3 R4	GGCAGTAGGGCAGGTCATTG			
**tgfbR2**	Accession:NM_182855	tgfbr2 F1	CACACATGCCAACAACATCA	generate in situ probe	ApaI	Sp6
		tgfbr2 R1	TCTCATTTGTCGTCGCTCAC			
	Ensembl ID:ENSDARG00000034541	tgfbR2 F2	TCAGTCCGGATCACACGATA	verify splice MO		
		tgfbR2 R2	CGACAGCGAGTTGTCCAAAC	verify splice MO		
**gata2b**		gata2b F1	ATGATGGATGCCCCAGCG	generate in situ probe	SacI	T7
		Gata2b R1	TCAGCCTATAGCAGTGACTAAGC			
**jag1a**	Yamamoto et al, 2010	jag1a F5	GACAGACAAACCGGGATGAT	verify splice MO		
		jag1a R5	CACCGCTTTCTCGATCACTT			
**rspo1**	Ensembl ID:ENSDARG00000039957	rspo1 F5	AGAAGCTCTACTCCATGGCTTG	qPCR		
		rspo1 R5	GACAGAGGCCTGGTTTATTTTG			
**baxa**	(Danilova et al., 2011)	Bax F1	CGTCGGGTGGAGGCGATACG	qPCR		
		Bax R1	GAGTCGGCTGAAGATTAGAGTT			
**baxa***	Accession:NM_131562	Bax F2	GGAGATGAGCTGGATGGAAA	qPCR		
		Bax R2	GAAAAGCGCCACAACTCTTC			
**p53**	Accession:NM_001271820	p53 F1	TTAAGTGATGTGGTGCCTGCCT	qPCR		
		p53 R1	AGCTTCTTTCCCTGTTTGGGCT			
**ef1a**	(Bertrand et al., 2008)	ef1a-F1	GAGAAGTTCGAGAAGGAAGC	qPCR		
		ef1a-R1	CGTAGTATTTGCTGGTCTCG			
**bactin2**	Ensembl ID:ENSDARG00000037870	bactin2 F1	GGACCTGTATGCCAACACTGTA	qPCR		
		bactin2 R1	ATGTGATCTCCTTCTGCATCCT			
**gapdh**	(Simoes et al., 2011)	gapdh F1	GGTCATTGATGGTCATGCAATC	qPCR		
		gapdh R1	CACCTGCATCACCCCACTTA			
**taz**	Ensembl ID:ENSDARG00000041421	taz F1	GGAGAATATCCAGCCGAGTG	qPCR		

	Ensembl ID:ENSDART00000138805	taz R1	TGCACCATCAGCGAGTTAAA	
**cdkn1a**	Ensembl ID:ENSDART00000136722	cdkn1a F1<br>cdkn1a R1	AAGTGGAGAAAACCCCAGAGA<br>TAGACGCTTCTTGGCTTGGTA	qPCR
**cdkn1a***	Ensembl ID:ENSDART00000136722	cdkn1a F2<br>cdkn1a R2	AACGCTGCTACGAGACGAAT<br>CGCAAACAGACCAACATCA	qPCR
**cdkn1b**	Ensembl ID:ENSDART00000076417	cdkn1b F1<br>cdkn1b R1	ACGGGAATCACGACTGTAGG<br>CACGATGAGTCGAGACAGGA	qPCR
**jag1a**	Ensembl ID:ENSDART00000137172	jag1a F3<br>jag1a R3	ATTGGTGGATACTTCTGCGAGT<br>CCATTCACCAGATCCTTACACA	qPCR
		jag1a kozak F<br>jag1a V5 R	GCCACCATGATTCTCAGACCGAGCGC<br>CTACGTAGAATCGAGACCGAGGAGAGGGTTAGGGATAGGCTTACCTACGATATACTCCATTTTCTGCAAG	amplify jag1a+C-terminal V5 tag
**gata2**	Accession:NM_131233	gata2 F3<br>gata2 R3	GGACGAAAAGGAGTCCATCA<br>GCACTCATAGCCAAGCTTCC	qPCR
**dll4**	Ensembl ID:ENSDARG00000070425	dll4 F4<br>dll4 R4	ACGCATACAACCCTAACATGC<br>CTCTGTCTGCTTCCCACTTTG	qPCR
**tnfr2**	(Espin-Palazon et al., 2014)	tnfr2 F<br>tnfr2 R	CACACAAGAGATCCGAAGCA<br>GGCATCTGTGATGGGAACTT	qPCR

*primers used for qPCR experiment shown in Figure S5K.

Sequence of the NanoString Probes Used in this Study.

Pathway	gene name	accession number	target sequence
**VegfA signalling**	vegfaa	NM_131408.3	TATTTCTCGCGGCTCTCCTCCATCTGTCTGCTGTAAAGGCTGCCCACATACCCAAAGAAGGGGGAAAGAGCAAAAATGATGTGATTCCCTTCATGGATGT
	vegfab	NM_001044855.2	ATGGACTAAAAGTACGCATGGATATCGCCTTCTCTCTTCAGCGCCTTTGTGTTCACTGATATCTCTGTATACTCCGCTAATAACACAGTGTCGGCAATAC
	VegfC	NM_205734.1	TCAGCAAGACGTTGTTTGAAATCACAGTTCCAGTCAAGCAAGGGACCAAACCGGTCACCATAAGCTTCGCCAACCACACTTCCTGCAGCTGTTTGTCAAA
	kdrl (flk1)	NM_131472.1	AACATACCCAAACCAAAACGTTATCCTTGAGACGCAGATGAATCCTATGGCAGATGATGTTAAAAGAGGGGTACAGTGGGATCCAAAAAAAGGTTTCACG
	kdr	NM_001024653.2	GCCCTATTGAAGGACAGGATGTTATAATGCGTTGTGTAGCAGACCGGCTGCTGTACTATAACTTGCGCTGGTATCGGGTAGCAAATGTAGCCAACCATGA
	mflt1	NM_001014829.2	GAAGCACTGGTTTCTGGCATCTATCGCTGTGTCACATCCAACATATTAGGCAGAGATGAACTAGACATTCCTTTCTATGTCACAGATGTCAAAGAAGGCC
	sflt1	NM_001257153.1	CAGCTTCCCAGCAGCGTGATTGTCCCTCCGTCTTCAAACGTGGCACAGTCCGCTTCCTCAGGAGCTTGATCTATTCTTGAGAGTCCCTGATCCCACACAC
	flt4	NM_130945.1	TCCTGACCTAAAAGTCACTCTCTTCTCGTTAGTGCCGTATCCAGAGCCTGTGGATGGCAGTGTGGTCACCTGGAATAATAAAAAGGGTTGGTCGATTCCC
	plcg1	NM_194407.1	TTTTACGCAGTGGATCGTAACCGAGAGGACAGAATTTCCTGTAAGGATCTGAAATGTATGTTGTGTCAGGTCAACTACAGAGTGCCCAACATGAAGTTCC
	tel1	NM_001044968.1	CCTGGTGGAACTAGTGGACTATTTCCGAAAGAAGCCGCTGTATCGAAAAACAAAACTGCGCTATCCTGTTACACCAGAACTAGTAGAGCGCTTCAGTTCG
	cbfa2t3	ENSDART00000021009.2	GCAACACTGCACGTTACTGTGGCTCTTTCTGTCAGCACAAGGACTGGGAGAAACACCATCATGTATGCGGCCAGGGTCTGCCTAGCAGTAGCGAGAGCAC
**Notch signalling**	notch1a	NM_131441.1	CAACTCTGATGATTGTGCATCTCAGCCGTGTCTCAACGGAAAATGCATCGACAAAATCAACTCGTTCCACTGCGAATGCCCTAAAGGGTTTTCTGGGAGT
	notch1b	NM_131302.2	GCCCGAGCAATTCAAGTAGAACAATGAGATTCGCTAAATGAAGGCACATTCCATAAGCCTGCTTATCTGAAGGGTCTTTATATGCCGCTTGTACTAACCT
	notch2	NM_001115094.1	CCATTCGGGATTCCTGTTTACAGCCATCTGCGATCTGAGGCAATGTTACCATTGAACGATCACACTGCCAAAAGTAAGGAGTCTCATTTTGGGGGTACTG
	notch3	NM_131549.2	TCAAACAGCTTGGACTGCATACAGTTGCCCAATGATTACCAATGTGTGTGCAAACCTGGCTTTACAGGACGAGGGTGTCAAAGTAGATTCAGCGTGTGCG
	dll4	NM_001079835.1	TCACCTTACTCGGATCTACCTGTAGACGCATACAACCCTAACATGCAGATAAGCTTTTTTGCCATCCAAAGAAAGTTGGAAGTAGGGGCTGAATGGTCTC
	dlc	NM_130944.1	GTCAACATTCACTGGTGGTAACTATTTCCGACATTCCCAAGGCTTGAAAGGACCCTTGGTCCAAACTGATAGCCTCAACATTGTCAAGATTTCCTGGGTT
	dld	NM_130955.2	AAGGACTTGTCCGTAAGCATCATCGGAGCCACGCAAGTGAAAAACATCAACAAGAAAGTGGACTTTCAGAGCGACGGCGACAAAAACGGATTCAAATCGC
	jag1a	NM_131861.1	GATGATTCTCAGACCGAGCGCAACTTTTGCGGCGCTGTCCGCTCACGTGCTGCTGAGGTGCTTGTGGATGCGGGTATGTGAGGCTTCGGGGCATTTCGAG
	jag1b	NM_131863.1	GGCTTGCAGTTTCGGAACTGGATCTACGCCCGTTCTCGGTGGGAATAAATTTTCGACCAAGGGAACGCGCAGTGAAAAGTCAAGGATTGTTTTACCCTTC
	jagged2	NM_131862.1	TGAAGACCCACAATCCCTTCCCTTACCAGTCCTCCTGTTGCTGATCATAAAACCGAGACCTGCCGGTTCATGCTTGTTGCTTGTAAAGATGCTGATATGT
	hey1	NM_212561.1	AACATCAGACTATTTTGAACAGATGCACAGAAACAGACTTGTACAAAGGGAAGACACGAATACAGATCCACTCTACTAACCAGAACGACTGTGCTTGTCC
	hey2 (grl)	NM_131622.2	GGAATGAAGTTTGAGACCTCCATTCGACGGCTCGGGGCGTGTTTTCTATTTTTTTTTTACGGTGGGTGTTCCCGAAGCAGGACGTGGGCGTGAATGTGAG
	efnb2a	NM_131023.1	ACTTTGGAGTTTTAGTGATCGCGTGCAAGGTGAACCTGTCCCGCGCGCTCATCCTGGACTCCATATACTGGAACACCACGAACACCAAGTTTGTGCCGGG
	ephb4a	NM_131414.1	ACCTTGTAAACATTCACAAGTAAAACACTCGTGATTCCGCGATGGAGCTCTTCTCCAGGAATGTGGCCGCGTTTTGGGTTATCCTGCTGGAGTTCCTGCT
**TGFβ signalling**	tgfb1a	NM_182873.1	CTGGGAACTCGCTTTGTCTCCAAGGACTTGTCCAACCGCTGGCTCTCATTTGACGTGAAACAGACAATGATAGAATGGCTGCAGGGTTCAGAAGATGAAG
	tgfb1b	ENSDART00000134907.2	TGCAAGTGTTGCTGAAAGCATCATTTCCGTTTGGTCTTGATGAGCAAAGTTAAGAGAACAACACACTGAGGTGCTTGAGACCCAGCACTTTCTGAGGGGA
	tgfb2	NM_194385.1	AAGTGCACAAGATAGACATGCAGCCCTTTTACCCTTCAGAGAATGTCATCTTATCACAACATTACTACCCATACTTCAGGAGGCTGATGTTTGACGTGAG
	tgfb3	NM_194386.2	CGCCTAAAGTGGAGCAGCTTTCCAACATGATCGTCAAATCCTGCAAGTGCAGCTGAGAGGTCCGTTTTCAACCCATCCACGAGCCAAAAGCTTCTGCGTT
	eng	ENSDART00000125008.2	GTGCGTTGGGTTTTGGAAAATGAGGGACTGTCAAGCAACATCAATGTGCTGGTGCAGTTGTCTGTGAACTCCACGGCAGAGTCTTCGAGTCTGTCTGTCC
	tgfbr3	ENSDART00000109313.2	AGCTCACAGAACGAATTTTACCAGTAAACGGACATCATGCGACTATTTTGTTCAACTGCACCTACCGCAAGAACCAGGACAGTCCATTTGACACCGATGC
	acvrl1	NM_153643.1	ACGAAAACCAGACATGAAGGCTTCAGTGATGCTCGCAGTGCTGTTTGTTTTGGTGTATTCTGGAAGCTATGCAACAACTGCAGATCATCTTATGGAGACT
	tgfbR2	NM_182855.2	CTTCAAGCAAACCGATGTCTACTCTATGGCTCTAGTGCTGTGGGAAATCACGTCCAGATGTAACGCCATCGGAGAGGTTAAAGACTATGAACCTCCATTT
	tgfbr1a	NM_001037683.2	CAAGGACTGCTGTGTTACTGTGAACGATGTGTCAATCGGTCGTGCAACACAACTGGACTGTGTTTTGCCGTCATCGCTAAGTCTTCTGGACAGACCGTGA
	tgfbr1b	NM_001115059.1	CATGAAGCATTTCGAGTCCTTCAAGAGGGCTGATATCTACGCCATGGGCCTGGTGTTCTGGGAGATCGCCAGCCGCTGCTCTATCGGAGGTATTCATGAA
	smad1	NM_131356.1	CCCCGTGCTGGATTGAGATTCATCTTCATGGACCCCTGCAGTGGTTGGATAAAGTCCTCACCCAGATGGGATCTCCTCACAACCCCATTTCTTCAGTGTC
	smad2	NM_131366.2	CTGCAGCCAGTGACTTACTCAGAGCCTGCGTTTTGGTGCTCCATAGCTTACTATGAACTTAACCAGCGGGTCGGAGAAACATTCCACGCCTCTCAGCCTT
	smad3a	NM_131571.2	CCCACAACAATTTAGATCTACAGCCAGTGACATACTGTGAACCAGCATTTTGGTGCTCTATTTCCTACTACGAATTGAACCAGCGAGTAGGAGAAACTTT
	smad3b	NM_175083.2	ACAGCCTCTGGACTCCTCAGAGTGTTACTTTATCAATTACAGATCCCGATATTTGCTGTGGGCAAAAACTGCTGCATACAGTTGAAGCACTTTGAACGGT
	smad4	ENSDART00000035478.2	AGTTTGACACCCCTGCTTTAAAGTAAACATTTAACCGTGTCTCTTTGTGCAGTTTCTCATAACCAAAGCTATTTTCTCCTCAGGGCCTGTTCACAATGAA
	smad5	NM_131368.2	CGGTGTCTGTAAAACAGAGCAAAGGTCGACAGAGTCTACAGCATTTCTTGAAAGGGATGGTGGCTTTTCCGCAGGGAGGTGTTGAGAAGTGATTGAAAAA
	claudin5a	NM_213274.1	TCTACATCCTCAGCGCCATCTTCGTGCTTGTGCCACTGTGCTGGATGGCCAATAATATCATCTCCGACTTCTATAACCCGCAGGTGCTGCCCGCCCAGAA
	claudin5b	NM_001006044.1	TTTCTGCATGTCTGGAGATTGTTGGACTCTGCTTGACCGTCACCGGGACTCTTTTGGTGATGGTAGCCTGTGGGTTGCCCATGTGGAAAGTGTCCGCCTT
	id1	NM_131245.1	GACAACTCAACAAGCCTTTAAAAGGAGTACGAGCACCTCGCTTCAGCTATTCCTCTTTGACTTACTCGCAAATAGCCAAAATGAAAGTTGTGGGACCTAC
	Id2a	NM_201291.1	ATATGTGCAGCATAGTGACCTTGCCTTTTATATCCAGGACTCTGAAGCATGTCACTGACCCTTCAAACTTGCACATTCGCCCAAGGAATGTTCTATTGAA
	Id2b	NM_199541.1	ACAGATGACCTCATAGCCCTGTCTCGTTGAAATGGTGAGTTGATTCATCTTCATGCTTTGACAAGCCAAGTTAAACTTTTGCACGTGGGGCAGTTTGCTG
	id3	NM_152967.1	AAACGAGACAGACACACAAAACACGCCTGACATTTTCCTGTCAATGAAGAACTCAGAGATGAGCCGTAATTTCTCCAAAGAAGATGGAGCTATGTGCCAT
	zeb2a	NM_001135104.1	ATATGATTCTTAAACGTACAAAGTCTTAAAGTTGGCCAGGGGTGGACGGAAAGAGTCTGGGTAACTATTGACGGAGGCTCATTGTATGAGGCAATGTCAG
	zeb2b	NM_001245966.1	ACTGGATCGGAGACGGAAGAAGAAGACAGACTTTTGGTATCGGAGGAGGACGCTTTGCTTAATGGGGCGGGGAGTCCGGCCAGCCTTGTCAATCATGAGT
	twist1a	NM_130984.2	GATGCACGCGTTTGATGCAGCATGATTCTCGGCCTGAGGAGCTGAACTCACTGGAAGGAGCGGCTCAAAACAAGGGCGAAAATAAGGATTATAAAGGGAA
	twist1b	NM_001017820.1	AATCTTGGGGAAAACGGCAAATGTTCCAACAGAGGTCATGGCTGTTACCGAGAGAAGGCCACGGGCAGCGAATTGTCATATGGATTTCCTCGCGAGTCTT
	twist2	NM_001005956.1	ACGCGTCCTGATAATGCCGAACGGACTGTTTACTTCCACTAATTTTGAGGATGCCAAAGGATTATTCGATGAACCTCTAAACCTCAGTGACGTGGCCAAA
	snai1a	NM_131066.1	CCCTGACCGCCACATCTCTGAAAACAGGTGGCGTCTGCTGATTTCTCTAGAAACACACTTGGAGAGAGTCCTTATGGAGCACAGTTGACTGCTTTACTTT
	snai1b	NM_130989.3	AGACCCACTCCGAGGTGAAGAAGTACCAGTGCGGGTCGTGCTCTCGGACCTTCAGCCGCATGTCTCTCCTGCACAAGCACACACTGTCCGGCTGCTGTCC
	cdh5	NM_001003983.1	GGATTATGATTTTATACATGAGTGGGGACCTCGGTTCAGGACCCTGGCTCAGCTTTATGGAGTAGACGGCTCTGATTCGGATAGCTCCTACTGAAGTCCA
**Cell cycle and apoptosis**	cdkn1a	ENSDART00000113620.2	CACAGATAAACTCTTGACCTGCATCTACCTTCATCTGTCTGGAGCTGCATTCGTCTCGTAGCAGCGTTATTGACAGAAAACGACCTTCAGGAACCAGAGA
	cdkn1b	NM_212792.2	GGGGGAAGGGCTCCGATATTCACATCTCCTCCTCTGGGAATAATAACGACAATAACGTGGATGTTAACGGGAATCACGACTGTAGGGTAACGGAGCAAAG
	cdkn1c	NM_001002040.1	GAGCGAGACCAGAGCCGGTGGAATTTTAACTTTGAGACCAACTCGCCTTTGCCTGGAGATTACGAGTGGGAGGCGATTTCAGAGGACACTTTGCCGTTTT
	cyclinD1	NM_131025.3	ACACGGTGCAGAAATTCATGTATAAGTGGCTGCCTGTCTTGTTCTGAACACAGGAGACCTGGCAGGTCCCCACCAAGTCGTTGTCTTAATAAATGTGACG
	tp53	NM_131327.1	TGAGGGGCAGGGAGCGTTATGAAATTTTAAAGAAATTGAACGACAGTCTGGAGTTAAGTGATGTGGTGCCTGCCTCAGATGCTGAAAAGTATCGTCAGAA
	perp	NM_001256207.1	ATGGAAGTCAGTGGGTGCATTTGTAGGTGAGATTTTTGGCGTGAACTGTCCCTCTAACAGGTATACATGCATCTCTCCCATAGTTAATTCTGATAGGTGG
	baxa	NM_131562.2	GTCTCCACTGCAAGGGCTTCCAAATGTCTGTGAGTTGTATTTACAGGTACAAGATGAGCCTCTAAAGTTTATGAGGTGGCGTGGCAGCAGTTGAGCGCAG
	bcl2	NM_001030253.2	TGGAGGTTGGGATGCCTTCGTGGAGATGTACGGTCAGCAGAGAGACTCTGTGTTCCACCCGTTTTCATACCTAACAAAAGTGCTCGGCTTGGCGGCGCTG
	bcl2l1	NM_131807.1	GCTTTGCAGAGATCTTTGGAAAAGATGCAGCGGCGGAAAGCAGGAAATCGCAAGAAAGCTTCAAGAAATGGTTGTTTGCAGGAATGACCTTGCTCACGGG
	pcna	NM_131404.1	TGTGACCCTCTAAAGCCATTACATATCATTGGCAGAATTGCGGGCGCACTTATTAAATGATGGTAGTTTGGGCCTTAGCTTTACCAGGCGCTCCGTGGGC
	bmp4	NM_131342.2	GATTCGTTTTTAATCTCAGCAGCATCCCAGAGGACGAACTCATATCCACCGCAGAGCTTCGCGTCTACAGGCAACAAATAGATGACGCCTTCTCAGACCC

**BMP, Shh and angiopoietin signalling**	bmp2b	NM_131360.1	AAGTTTTCATCACGAAGAGGCTTTCGAGGCACTGTCCAGCCTGAAAGGAAAAACAACGCAGCAGTTTTTCTTCAACCTTACCTCCATTCCTGGCGAGGAG
	bmpr2a	NM_001039817.1	CAGTTCTGCTCTCCTGAAACGTCAGCCTCCCACGGCCCGTTTTATCCTCTCATGAAGATGGTTTCTGAGGTTTCGGGGTCACAAGGATCCAGTAGGCATG
	bmpr2b	NM_001039807.1	CACATGAGCTTGAACTTCGTTCGATAGAAAGAAGAAAGGGGGAACAGAAAATCTAGTTACTGGAACTTTTTTTGGAGCTGAATCCATCCAGAAGCGGCAG
	shha	NM_131063.1	CCACGACGCGACGTGTGTTTTACGTCATAGAAACGCAAGAACCCGTTGAAAAGATCACCCTCACCGCCGCTCACCTCCTTTTTGTCCTCGACAACTCAAC
	shhb	NM_131199.2	GAAACATACAAGAGATTATATGAGATATGAGAAAGCGCAGGGCTGTGCAGTGTGCAGCGCTCTATTTTATCGGATAACCATAAAGGATGGCGCTCAAAAG
	ptch2	NM_130988.1	GCCACGCCGCTTTTGCTTTAAAACAGATTTCTAAGGGGAAAGCTGTGGGACAGAAAGCACCACTGTGGATTCGGGCGAGGTTCCAGGCTTTTCTCTTTTC
	gli1	NM_178296.2	GAACATGATGACATCCCATCATAATCTCCCTCATAACCAGCACACATCTGAACTCATGGCATCAGGAGACGCCTCTTGTTTCTCCACTCCTCGCTCGATG
	gli2a	NM_130967.1	GTATTTTGTCTCATCTAAGCTGTGTGATACTGAAGGACAGCATCTCTGCGCTTCAGCCTGTGTAGGCTTGACGAACCTTGCGTCCCTTTGAGATGTCTTT
	angpt1	NM_131813.1	GCTGACGAGGAGCGAAAGTTTCGGGATTGTGCTGATCTTTATCAAGCAGGCTTCCAGAAAAACGGAGTTTACACCATCAATATTAGCCCACAAGAGACCA
	angpt2	NM_131814.1	TGCAGAGGAACACCAGGCTTATTCTCAGTATGACACTTTCTACATTGACGGAGAAGATAAGAAGTATAGTCTCCATGCCAGGGGTTTCAGTGGTACTGCT
	tie1	ENSDART00000003701.2	GGGATAAAAATGAGTGCTGCAATTAAAATGCTCAAGGAGTTCGCCTCAGAGAATGACCATCGAGACTTTGCTGGAGAATTGGAAGTGCTGTGCAAATTGG
	tie2	NM_131461.1	GCGGCTCGAAACGTCCTGGTGGGAGAGAACTTTGTGGCGAAGATCGCAGACTTCGGGCTGTCCAGAGGTCAGGAGGTGTACGTCAAGAAGACCATGGGTC
**Haematopoietic/vascular genes**	fli1a	NM_131348.2	ACTTCCTGAGACTCACCAGCGTTTATAACACCGAGGTCCTTCTCTCACATCTCAATTACCTCAGGGAAAGTAGCTCATCGATATCATACAACACGCCATC
	fli1b	NM_001008780.1	GTAATTTCTTCACGCCTCAATCCACCTACTGGAACTCCGCAACCAGTGTGGTTTATCCCAGTTCACCGATGCCACGACATCCCAGCACTCACACTCACTT
	aplnra	NM_001075105.1	TCAGCTTCCAGTGAGATAAACTTTACCACAGACTCTTGTATCAGACAGAAGAATCCAAACATTTTCAGTGAACTCTATAAAAACTGCTGCCAGAGATTGG
	nr2f2	NM_131183.1	GTATTACAGACGCTTACCTAAGGTTGCGCCCAAGCGAATTTTCGCTCAAGTGTTTAGCCTCAGTAAACTGTATTTGTGAACGTGGGGAAATGATGGGTCA
	dab2	NM_205757.2	GGCCAGATTTCAGGGTGATGGAGTGAGATATAAAGCCAAACTTATTGGTGTGGATGATGTTCAAGATGCAAGAGGGGATAAAATGTGTCAGGATTCCATG
	scl alpha	NM_213237.1	TAGCAATCGAGTCAAGCGCAGACCTGCACCTTATGAGGTTGAAATCAACGATGGTTCGCAGCCCAAAATTGTGCGACGGATTTTCACGAACAGTCGCGAG
	lmo2	NM_131111.1	GCGTACACAATGTGTGCTGGATGTTTCTGACCTTTGATACACTTGCTAAGACAGCAGAACAGGTGCATCTCTGAAGCGTTTTGTGCGGCAGATGGTCTTT
	lmo4a	NM_177984.1	ACATCTTCCCTCCAATCTCTCCTCGTGAATGTCCACCTTCACCCGAGACCTTCTGCCCACTTCTGCGCATCCAGCCACCTTAAAAGAGTTCAGAACTACG
	lmo4b	NM_212689.1	TCTCAAGTGTTTCACATGTTCTACCTGCCGGAACCGGCTTGTCCCTGGTGACAGGTTTCACTACATCAACGGCAGCTTGTTTTGTGAACACGACAGACCC
	ldb2a	NM_131314.1	TGTCCAACCCTCCGCACGACCCCTTCTACTCGTCCCCTTTCGGACCCTTTTACCGGAGACATTCACCGTACATGGTGCAGCCCGAGTACAGAATCTACGA
	tbx20	NM_131506.1	GTTTCACGAACTCGGCACTGAAATGATTATCACAAAGTCTGGAAGACGAATGTTTCCGACAATTCGTGTGTCATTTTCCGGAGTGGACCCAGACGCCAAA
	hhex	NM_130934.1	GAGCATCAGTTCACCCTATGATAAGCCCTGCTCTTACTGTAAATACTGCACCATACTGTACATTCCGCCATACCTGACCACTGACTTAATGGATGTGTAT
	gata2a	NM_131233.1	ATTTACTGAGTCACTTTGGTACTGAAAGAGCGGACGCAGAATCACTGTGTGGTAGTCAAAACGGCCACCTCAAAACTCTCATAAAGGACTCGCTTTGAGC
	gata2b	NM_001002689.1	CCCTATACCTTCATATCCCGATTACTCAGTAGCCGGAGCGCACGAGTATCCCGCCAGTGTGTTTCACTCCAGAAATCTGCTCGGAAACATGACGACAAAA
	etv2	NM_001037375.1	CTTTGGCAGTTTCTGCTAGAACTCCTGCTGGATTCTGCTTGCCACACTTTTATAAGTTGGACTGGTGATGGCTGGGAGTTTAAAATGTCAGATCCCGCTG
	gata1	NM_131234.1	ACAGACTCTGGTTTACTGCCACCCGTTGATGTAGATGAACCTTTCTACTCAAGCTCTGAGACTGACCTACTGCCATCGTATTATTCCACCAGCGTCCAGA
	tif1g	NM_001002871.1	ACTCGAATCGTGAACGTCATGCGTTGTACAGTGTGCCATCAGGACTACAAACAGATTGACATGGTGGACAATTACTTCGTCAAGGACACTTCAGAGGCCA
	alas2	NM_131682.2	GCGAGTGGGAGAAGTCTTATTTTGGTAACATGGAGCCAAGATACATCACTGTGGCTGCACAATGAGTCCATTTTAACAGCTCAAGATGTAGATCTTCCAT
	gfi1b	XM_001922262.1	AAAGAGCTTCGGCCATGCAGTCAGTCTAGAGCAACACATGAACGTCCACTCACAGGAAAGAAGCTTTGAGTGCAAAATGTGCGGTAAAACCTTCAAGCGC
	spi1	NM_198062.1	TCTTGGGATTATAACACGCATCACATTCATCCAGTGGACTTCGAGAATCTGCCGGAGAGCCATTTTACAGAGCTACAAAGCGTGCAGTCACTACATGCGG
	mpx	NM_212779.1	GAGAGAGGCTGTTTGCCTTCACATCCCACATAGCTTTGGATCTGGCTTCCCTCAACATGCAAAGAGGGCGTGACCATGCTATACCAGGTTATAATGCATG
	mpeg1	NM_212737.1	TAAAACCAGTTCGTCTTCCACCGTTCACAAGACCACCAGTGGTCGGCATGATTGCGACAAACACAGTAGCCGTAATGACAGAAGGTGAGCGTTCTTGGGT
	csf1ra	NM_131672.1	TGTACTGAAGGAGTGAAAGATCAGTCTATATTTCCTCTGAGACGCTGAAGACTGCTGAATGGTTAAGCAGGAGCAGCTCTGAGATGTTCTTCGCGCTCTT
	cebp1	NM_131837.1	AGCGCTCAGACTCCGATGGACTCGGCCCTGTATACTCAGACCATCAGCTTTACTAAGAGTCCCGAGGTGATGATGGGTTATTTACCGTACTCCTCCTGTC
	lcp1	NM_131320.1	ATGAGGAGGATCTAGAAGCGGATTCCATTCCACTGGTCGTCTCCTATACTCCATTATACGCCTGAAACAGCTCAGAGTACGCCACGCTGAACTGTGGACA
	cmyb	NM_131266.1	TGAAGACTGGAAAGTCATCGCCAGCTTTCTACCGAATCGAACAGATGTTCAATGCCAGCATCGTTGGCAGAAAGTCCTCAACCCTGAACTCATTAAAGGA
	ikzf1	NM_130986.1	GAGAGAATCGTCTGTCAGAGCTATCTTTCGAGAGTGGCTCAGGTGAGCTGATGCAGCCCCATGTGATTGATCAGGCCATCAACAGTGCAATTAGCTATCT
	runx1	NM_131603.2	AAAAGCAGACACTTATGCTCTAAAATGTCGTCACTAATGACTAAATCACTGATGAATATCCACAAGATAAAGGTAAACATCGCTGAAGTGTAGCTCTGAC
	cbfbeta	NM_199209.1	TCCCGGATCAGAGGAGCAAGTTCGAGAACGAGGAGTTCTTCAGGAAACTCAGCCGCGAGTGCGAGATTAAATACACCGGTTTCCGCGATCGGCCGCACGA
	itga2b	NM_001003857.1	TGCTGTCGCTTCCCTGGAGATGCGTGGAGTCTCTTCTCCTGTTGACTGTGTCCTGCCTATTTCTAAATGGGAGTCGAAGGATTATCCGGAGGACTTGGAT
	dnmt4	NM_001025450.1	TGTAGCACTCTGATGCCTTAATGTGTGGTGCCTTTTGAAGAGAACAGATACAGCCTGCCATACGGGGGTTGCGAAGAAACTTTCTGCCTTTGTTTGCTGT
	gfi1.1	NM_001020776.1	ACAGGCTAGATTGGAGAGCTGTGAGTGATTGGTGACCCTGAAGCTGATAACGGAGAGTAAACATGCCGAGGTCATTTTTGGTGAAGAGCAAACGGGCGCA
	gfi1	NM_201338.1	AGCGGCCTACTCCAACCTATTACAATGAGCGCGTGACCGAGCCCTCTATTTTCACCGAACGAGGATCTGGCGCAAGAATTTACAACAGCTATGGCTCCAC
	hbbe1.1	NM_131759.1	GCTGATTGCTTGACCATCGTTGTTGCTGCTCAGATGGGTGCTGGATTCACACCTGAAGTTCAGGCCGCTTTCCAGAAATTCATCGCTGTTGCGGTGTCCG
	urod	NM_131347.1	TGGGTCACGGCCTTTACCCTGATATGGACCCAGAAAATGTGGGCGCATTCGTGGAGGCTGTACATAACCACTCTCGCCAGCTTCTCAAACGCTAATAAAA
	ptprc	ENSDART00000105607.2	ATGCTTCATTCTTAGATGGCTACTGGTGTCAGAAGAGTCTGATCGCAGCACAAGGGCCTTTACCAAACACAACAGCAGAGTTTCTGCTCATGCTGTACCA
	klf2	NM_131856.2	CTGGACCTAAACCTCAAGACGGAACTGGAATCAGACAGTATAATGTGCTTTAAGTTAACATGATGTGCTGCGGACATCACGACAGTGCCTTTTAATATGC
	klf4	NM_131723.1	TTTTAATATGTTTAGCGAACCACTGCGGGCAAATCACCCGGCCATGCCAGGTGTGATGCTGACTCCACCATCCTCACCTCTCCTGGGATTTTTAAGCCCA
**Wnt signalling**	rspo1	NM_001002352.1	TTCATCTTACTGGAGCGAAATGATATCCGTCAGATAGGCATTTGCCTGGCCGCGTGTCCTGTTGGATATTATGGCATTCGAAATCGGGATATGAACAAAT
	wnt2bb	NM_001044344.1	CCCTTGGTGCACGTGTGATCTGTGACAATATTCCTGGGCTGGTGAATAAACAGAGACAGTTGTGTCAGAAATATCCTGACATCATGCAATCGATAGGTGG
	wnt16	NM_001100046.1	CCAAAACAATGTTGACAGACTGCCGCTGTCATGGCGTATCGGGTTCATGTGCGGTAAAGACGTGTTGGCGCACAATGGCTGCATTTGAGCGTGTGGGCGC
	fzd2	NM_131140.1	GAAAGACACTGCACTCTTGGCAGAAGTTCTATGTGCGTTTGACCAGTGCTGGACAAGGAGAAACCACTGTTTGAAACGAGACATTGTGATCCACAATTTC
	fzd7b	NM_170763.1	ACAACGGTGTGAACTGAACTTTGTGGACTGCATGTGAGAAATACAGGTCAATATAGAAAGGAAACGTGAACTGCGATCAGGTCACACATTTGCTGATGGG
	taz	NM_001001814.1	TCTGGGGAGTCCTTAAGTTCCGGCAGTTGTGGAACCTTAACAAGATGAGATGGACACCTGCTGCCTCTGATATCTGTTTCACAAGAGAATTTCACTCCAG
**Housekeeping genes**	actb1	NM_131031.1	GATCTTGCAGGACTTCCCTAGGGTATGTGAATAAGGGATGTCCCTTGAAAATGTAAGCCAGGGTGTCTCTGTACACTGACAAGTCAACCCAAATAAAACG
	actb2	NM_181601.3	CCTGGGCATATTGTAAAAGCTGTGTGGAACGTGGCGGTGCCAGACATTTGGTGGGGCCAACCTGTACACTGACTAATTCAATTCCAATAAAAGTGCACAT
	gapdhs	NM_213094.2	GAGGTTTAGCACCACAATCCACACTCTTGCAATAATGTCTGAGCTTTGTGTTGGAATCAATGGATTTGGCCGTATTGGCCGTTTGGTCCTCAGGGCCTGC
	ubic	NM_001077804.1	AGATGGACGCACTCTGTCCGACTACAACATCCAGAAGGAGTCCACCCTCCACCTGGTGCTGCGTCTTCGTGGCGGCAAATAATCTGCTTATGATTAATCA
	sdha	NM_200910.1	AAGACTATGCAGAGTCACGCAGCTGTGTTCCGTACTGGAGATGTTCTGAAGGAGGGTTGTGTCAAGATGGAGTCCGTCTATAAGTCGATGGATAACATTA
	eef1a1a	NM_200009.1	CTTCGGTGCACTGACAGCTTTATGTTTAGCACTTTGACTGGCGTAGGCTAACCCTGCACAAGCACTTGTTGACTTTAATTGGCATTAGCTACTGCACCTT
**pronephric duct**	chordin	NM_130973.1	CGACTCTGGGAGGAAGGAGGTCGAGTCTCTGTTTGATTTCTTCCAGGAAAAAGATGACGATTTGCACAAATCCTACAACGACAGATCGTACATCAGCTCC
	cdh17	NM_194422.1	GCACTTTTGCTACACTTGACCCTTCTCGTCAGCATTGGTCATGGGATTGATCTAGAGGACAAAAAGGGGCCATTAATAGATACGGTTTTAGATGTGCCAG
	pax2a	NM_131184.2	CCATTCTTGAAGAGAGGGGGACTGAGAGACACTCGCCGTGTCCATGTTTTTTCCTGTCATTCCCGCACGAATCTGACTGTACCCCTCAGACTGTTCAGAC

Supplemental References

Bahary, N., Goishi, K., Stuckenholz, C., Weber, G., Leblanc, J., Schafer, C.A., Berman, S.S., Klagsbrun, M., and Zon, L.I. (2007). Duplicate VegfA genes and orthologues of the KDR receptor tyrosine kinase family mediate vascular development in the zebrafish. Blood *110*, 3627-3636.
Bertrand, J.Y., Kim, A.D., Teng, S., and Traver, D. (2008). CD41+ cmyb+ precursors colonize the zebrafish pronephros by a novel migration route to initiate adult hematopoiesis. Development *135*, 1853-1862.
Cheah, F.S., Winkler, C., Jabs, E.W., and Chong, S.S. (2010). Tgfbeta3 regulation of chondrogenesis and osteogenesis in zebrafish is mediated through formation and survival of a subpopulation of the cranial neural crest. Mech Dev *127*, 329-344.
Chocron, S., Verhoeven, M.C., Rentzsch, F., Hammerschmidt, M., and Bakkers, J. (2007). Zebrafish Bmp4 regulates left-right asymmetry at two distinct developmental time points. Dev Biol *305*, 577-588.
Clements, W.K., Kim, A.D., Ong, K.G., Moore, J.C., Lawson, N.D., and Traver, D. (2011). A somitic Wnt16/Notch pathway specifies haematopoietic stem cells. Nature *474*, 220-224.
Danilova, N., Sakamoto, K.M., and Lin, S. (2011). Ribosomal protein L11 mutation in zebrafish leads to haematopoietic and metabolic defects. Br J Haematol *152*, 217-228.
Espin-Palazon, R., Stachura, D.L., Campbell, C.A., Garcia-Moreno, D., Del Cid, N., Kim, A.D., Candel, S., Meseguer, J., Mulero, V., and Traver, D. (2014). Proinflammatory signaling regulates hematopoietic stem cell emergence. Cell *159*, 1070-1085.
Geiss, G.K., Bumgarner, R.E., Birditt, B., Dahl, T., Dowidar, N., Dunaway, D.L., Fell, H.P., Ferree, S., George, R.D., Grogan, T., *et al.* (2008). Direct multiplexed measurement of gene expression with color-coded probe pairs. Nat Biotechnol *26*, 317-325.
Gering, M., and Patient, R. (2005). Hedgehog signaling is required for adult blood stem cell formation in zebrafish embryos. Dev Cell *8*, 389-400.
Jin, S.W., Beis, D., Mitchell, T., Chen, J.N., and Stainier, D.Y. (2005). Cellular and molecular analyses of vascular tube and lumen formation in zebrafish. Development *132*, 5199-5209.
Kolde, R. (2013). pheatmap: Pretty Heatmaps. R package version 0.7.7. (http://cran.r-project.org/web/packages/pheatmap/index.html).
Link, V., Shevchenko, A., and Heisenberg, C.P. (2006). Proteomics of early zebrafish embryos. BMC Dev Biol *6*, 1.
Livak, K.J., and Schmittgen, T.D. (2001). Analysis of relative gene expression data using real-time quantitative PCR and the 2(-Delta Delta C(T)) Method. Methods *25*, 402-408.
Monteiro, R., Pouget, C., and Patient, R. (2011). The gata1/pu.1 lineage fate paradigm varies between blood populations and is modulated by tif1gamma. EMBO J *30*, 1093-1103.
Simoes, F.C., Peterkin, T., and Patient, R. (2011). Fgf differentially controls cross-antagonism between cardiac and haemangioblast regulators. Development *138*, 3235-3245.
Waggott, D., Chu, K., Yin, S., Wouters, B.G., Liu, F.F., and Boutros, P.C. (2012). NanoStringNorm: an extensible R package for the pre-processing of NanoString mRNA and miRNA data. Bioinformatics *28*, 1546-1548.
Yamamoto, M., Morita, R., Mizoguchi, T., Matsuo, H., Isoda, M., Ishitani, T., Chitnis, A.B., Matsumoto, K., Crump, J.G., Hozumi, K., *et al.* (2010). Mib-Jag1-Notch signalling regulates patterning and structural roles of the notochord by controlling cell-fate decisions. Development *137*, 2527-2537.
